# Olfactory Receptor Responses to Pure Odorants in *Drosophila melanogaster*

**DOI:** 10.1111/ejn.70036

**Published:** 2025-03-10

**Authors:** Alja Lüdke, Ajayrama Kumaraswamy, C. Giovanni Galizia

**Affiliations:** ^1^ University of Konstanz Constance Germany

**Keywords:** calcium imaging, *Drosophila melanogaster*, odor‐response profiles, olfactory impurities, purified odorants

## Abstract

Olfactory coding relies on primary information from olfactory receptor cells that respond to volatile airborne substances. Despite extensive efforts, our understanding of odor‐response profiles across receptors is still poor, because of the vast number of possible ligands (odorants), the high sensitivity even to trace compounds (creating false positive responses), and the diversity of olfactory receptors. Here, we linked chemical purification with a gas chromatograph to single‐receptor type recording with transgenic flies using calcium imaging to record olfactory responses to a large panel of chemicals in seven *Drosophila* ORs: Or10a, Or13a, Or22a, Or42b, Or47a, Or56a, and Or92a. We analyze the data using linear–nonlinear modeling and reveal that most receptors have “simple” response types (mostly positive: Or10a, Or13a, Or22a, Or47a, and Or56a). However, two receptors (Or42b and Or92a) have, in addition to “simple” responses, “complex” response types to some ligands, either positive with a negative second phase or negative with a positive second phase, suggesting the presence of multiple binding sites and/or transduction cascades. We show that some ligands reported in the literature are false positives, because of contaminations in the stimulus. We recorded all stimuli across concentrations, showing that at different concentrations, different substances appear as best ligands. Our data show that studying combinatorial olfactory coding must consider temporal response properties and odorant concentration and, in addition, is strongly influenced by the presence of trace amounts of ligands (contaminations) in the samples. These observations have important repercussions for our thinking about how animals navigate their olfactory environment.

## Graphical abstract

Odorants were purified by gas chromatography and elicited calcium responses in *D. melanogasters* olfactory sensory neurons, measured on the antenna. Different olfactory receptor types revealed their specific response profile. Calcium responses to different odorant concentrations were recorded to investigate strong ligands of a receptor type. Also, response dynamics were fitted with a linear–nonlinear model.

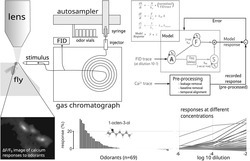

**Abbreviations**
BIC: Bayesian information criterion
DoOR database: a database for olfactory receptor responses, https://neuro.uni‐konstanz.de/DoOR/
FID: flame ionization detector
GC: gas chromatograph
Or10a, Or13a, Or22a, Or42b, Or47a, Or56a, Or92a: specific olfactory receptor genes/proteins in *Drosophila*
Odorants are abbreviated with a four‐letter code: See Table S1.

## 1 Introduction

There is a potentially illimited number of possible odorous substances in our environment: almost all organic (and some inorganic) substances with any volatility. However, animals can only express a finite number of different receptors for detecting odorants. These receptors interact with their ligand, activating a transduction cascade: some are narrowly tuned to few chemicals; others are broadly tuned. Many narrowly tuned receptors have been described in insects (Haverkamp, Hansson, and Knaden 2018). For example, in *Drosophila melanogaster* , activity in the receptor Or56a signals the presence of the repellent geosmin (Stensmyr et al. 2012), and Or67d the pheromone 11‐cis‐vaccenyl‐acetate (Benton, Vannice, and Vosshall 2007; Kurtovic, Widmer, and Dickson 2007). Other receptors respond to a broad range of substances, such as Or22a and Or10a (Münch and Galizia 2016). Combinatorial coding allows for recognizing many more odors than there are receptors (Malnic et al. 1999; Galizia 2014; Grabe and Sachse 2018). However, in order to understand the combinatorial code for any particular odorant, it is necessary to know the response profiles of each sensory cell in a species.

Across species, not many receptors have been characterized with respect to their ligands. The first receptor to be “deorphaned” was odr‐10, the diacetyl receptor in *C. elegans* (Sengupta, Chou, and Bargmann 1996), followed by the rat OR‐I7 to several *n‐*aldehydes (Zhao et al. 1998). The human receptor IR10G4 responds to guaiacol (Mainland et al. 2014). The species with the best description of receptor responses is the fruit fly *D. melanogaster* , with a first comprehensive odor‐response data matrix of 100 chemicals for 24 receptors (Hallem and Carlson 2006) and a large number of characterized receptors across several labs (Stensmyr et al. 2003; Kreher, Kwon, and Carlson 2005; Kreher et al. 2008; Yao, Ignell, and Carlson 2005; Pelz et al. 2006; Marshall, Warr, and Bruyne 2010; Montague, Mathew, and Carlson 2011; Silbering et al. 2011). Still, several receptors remain “orphans,” either because no ligand is known or because the best known response is so weak that we assume that a better ligand must exist (Münch and Galizia 2016). Furthermore, even if a receptor has been deorphaned (i.e., a very good ligand is known), in order to understand the combinatorial code of the olfactory system, weak responses to other ligands need to be quantified.

Given the big‐data nature of olfactory coding, with many receptors and many ligands, the scientific community is increasingly working on creating databases to collect odor‐response profiles. In *Drosophila*, the DoOR database has reached its second version (Galizia et al. 2010; Münch and Galizia 2016). In other species, odor‐response collections are also important data repositories (Skoufos et al. 2000; Nagarathnam et al. 2014; Sharma et al. 2022; Lalis et al. 2024). These large data collections may be used to understand the logic of odor‐binding or to create artificial olfactory sensors. However, so far, binding properties often do not yet take into account stimulus concentration nor the temporal properties of the responses.

Responses to odorants are not on/off but rather have a temporal complexity. For example, in *Drosophila*, responses to single substances can create sequences of excitation and inhibition (Münch and Galizia 2017). To understand the time‐course of odorant responses, it is useful to adopt a linear–nonlinear model, where the time course of the stimulus is used to estimate the time course of the response in a cascade of mechanisms, involving a linear and a nonlinear transfer function (Dayan and Abbott 2005; Martelli, Carlson, and Emonet 2013; Kato et al. 2014). This approach is useful to understand odorant responses also to temporally complex stimuli.

Odor‐response spectra can easily contain false positives: chemicals that are assumed to be ligands for a receptor, but indeed are not (Paoli et al. 2017). The reason is that even the purest substances commercially available have a (limited) purity of 95%–99.9%—leaving at least 0.1% to other chemicals. Recently, we have shown that an olfactory response to an odorant in our laboratory was entirely due to an undetected impurity of 0.0006% ethyl acetate, an impurity that had not been detected in our own chemical analysis of the substances (other impurities were much stronger) or in the chemical analysis of the manufacturer (Paoli et al. 2017). Under these circumstances, the only way to obtain reliable odor‐response data is to purify the stimuli on the spot, that is, to record responses to gas chromatographic eluates. Applying odorant stimuli via a gas chromatograph is common in chemical ecology studies, where key compounds for single receptors, or for ecological situations, are investigated. In these experiments, the eluate from a gas chromatograph is directed at the receptors, and their responses are quantified via single sensillum recordings, electroantennograms, or calcium imaging (Stensmyr et al. 2003; Røstelien et al. 2005; Burger et al. 2021).

Here, we combined gas chromatography with receptor‐specific calcium imaging, to record odor‐response profiles across a large panel of chemicals and measure dose–response curves. We report a large dataset for seven different *D. melanogaster* odorant receptors in situ, that is, within the living animal and with all accessory mechanisms intact. The measured lines were Or10a, Or13a, Or22a, Or42b, Or47a, Or56a, and Or92a, which include the highly selective Or56a, the broadly responding Or22a, and two receptors with many negative responses (Or42b and Or92a). Responses to these lines had previously been analyzed with respect to their temporal response profiles (Münch and Galizia 2017), prompting us to verify how temporally complex their responses are. We apply linear–nonlinear modeling to characterize the temporal response properties of these receptors. We show that most responses can be modeled with a single function, indicating a single transduction mechanism, but some necessitate two functions. Furthermore, best ligands induce long response tails at high concentrations. We characterize chiral sensitivity in the response to 2,3‐butanediol in Or92a. We show that apparent responses to chemicals are sometimes due to contaminations and report a few false positive ligands in the DoOR database. These data contribute to our understanding of odor–receptor interaction and to olfactory coding in general.

## 2 Methods

### 2.1 Odorants

All odorants used were of the highest purity available and bought from one of the following: Sigma‐Aldrich, Fluka, Merck, SAFC, and Santa Cruz Biotech; suppliers are listed in Table S1. Odorants were diluted in mineral oil (MOL, Acros Organics, CAS 8042‐47‐5) at 10^−2^, 10^−4^, and 10^−6^. In some experiments, further dilution steps were included to characterize very good ligands, or higher concentrations were used to characterize elution time of the chemical in the gas chromatograph.

Five milliliters of the diluted odorant in MOL was placed in 20‐mL glass vials (Schmidlin Labor + Service GmbH & Co. KG, Dettingen, Germany) in nitrogen gas to avoid oxidation and sealed with Teflon‐coated caps (Schmidlin Labor + Service GmbH & Co. KG, Dettingen, Germany). Preliminary experiments showed that headspaces were saturated after 10 min; generally, headspace concentration was allowed to stabilize for at least 30 min before every measurement. For the following measurements, the odorant‐loaded headspace of these 20‐mL vials was used and injected into a gas chromatograph. In most experiments, we mixed six to eight odorants into one vial, all at the same dilution. Fresh vials were prepared frequently (every 10–20 measurements).

### 2.2 Stimulation

One‐milliliter headspace was injected using a computer‐controlled multisampler (Combi PAL, CTC Analytics AG, Zwingen, Switzerland) into a gas chromatograph (GC, TRACE GC Ultra, Thermo Fisher Scientific, USA) equipped with a flame ionization detector (FID) and coupled to the imaging set‐up (see below). To avoid oxidizing the substances with air‐borne oxygen, headspace was obtained by first injecting 1 mL of nitrogen into the vial, creating pressure in the vial, and then removing that headspace. The headspace was injected into the GC at split mode with the injector temperature set to 200°C, the split flow to 15 mL min^−1^, and the split ratio to 10. The GC was equipped with a nonpolar GC column (Optima 5 MS 30 m × 0.25 mm × 0.25 μm column, Macherey‐Nagel, Germany) or a polar column (see below).

The flow of the carrier gas (helium was used for most experiments, and in later experiments, hydrogen was used as the carrier gas) was set to 1.5 mL min^−1^. The oven was held at 60°C for 1 min, then the temperature was increased to 200°C at 20°C min^−1^, and the final temperature was again held for 1 or 4 min, depending on the elution time of the last substance. The nonpolar column ended in a four‐arm flow splitter (OSS‐2, SGE, UK) that split the column at the end into two pieces of deactivated capillary (length 70 cm, ID 0.32 mm) leading to the detector (FID) and to the fly via a heated (temperature 200°C) transfer line (ODP3, Gerstel, Germany). Makeup gas (nitrogen, 2.5 mL min^−1^) was introduced through the fourth arm of the splitter. The transfer line ended in a glass tube directed to the *Drosophila* antennae for calcium imaging (inner diameter 7 mm, ending with a 2 mm opening, air stream 1 mL s^−1^, with the exit positioned ~2 mm from the antenna). Between successive stimuli, the headspace syringe was flushed with clean air and regularly washed with hexane.

In some Or lines, measurements were also performed with an additionally added polar column (POL) (Durabond: DB‐WAX, Length: 30 m, Diameter: 0.25 mm, Film: 0.25 μm; Agilent Technologies, Agilent J&W GC Columns, Part Number: 122‐7032, Temp. Limits: 20°C–250°C [260°C], Serial Number: USE464741H). In these experiments, the GC was equipped with two injectors and two columns (nonpolar and polar). Only one injector and one column were used for every single run (either nonpolar or polar). All parameters for the GC‐run were kept as above. However, the nonused column was set to a flow of 0.7 mL min^−1^ helium or H_2_ as makeup gas. Both columns ended in a three‐arm‐Y‐splitter, which was then connected to the above‐mentioned four‐arm flow splitter. FID data were recorded using Xcalibur software (Thermo Fisher Scientific, Massachusetts, USA).

### 2.3 Fly Lines

All recordings were performed on *D. melanogaster* fruit flies. For calcium imaging, we used the genetically encoded calcium sensor GcaMP6m (Chen et al. 2013), which was expressed under the control of UAS, using specific Or‐Gal4 lines. UAS‐GcaMP6m (either on the II or III chromosome) was kindly provided by André Fiala (Georg‐August‐University, Göttingen, Germany) or obtained from Bloomington Drosophila Stock Center (Indiana University Bloomington, USA). The specific Or‐Gal4 lines (Or10a, Or13a, Or22a, Or42b, Or47a, Or56a, and Or92a) were kindly provided by Silke Sachse and Veit Grabe (MPI for Chemical Ecology, Jena, Germany) or Leslie Vosshall (Rockefeller University, USA) or obtained from Bloomington Drosophila Stock Center. The crosses for obtaining the stable Gal4‐UAS lines were performed in our lab. Flies were kept at 25°C in a 12/12 light/dark cycle at 60%–70% RH. Animals were reared on standard medium (100 mL contain: 2.2 g yeast, 11.8 g of sugar beet syrup, 0.9 g of agar, 5.5 g of cornmeal, 1 g of coarse cornmeal, and 0.5 mL of propanoic acid).

### 2.4 Calcium Imaging

For calcium imaging, flies were mounted in custom‐made holders. The head was fixed to the holder with a drop of low‐melting wax. The third antennal segment was stabilized by gluing the arista to the holder. A half electron‐microscopy grid was placed on top of the head, stabilizing the antenna by touching the second, but not the third antennal segment (Münch and Galizia 2016).

Calcium imaging of dendrites and somata of olfactory sensory neurons on the third antennal segment was performed on a setup consisting of a fluorescence microscope (BX51WI, Olympus, Japan) equipped with a 50× air lens without cover slip correction (Olympus LM Plan FI 50X/0.5). Images were recorded with a CCD camera (either SensiCam, PCO, Germany, with 8 × 8 on‐chip binning, resulting in 80 × 60 pixel‐sized images, or Andor Technology, UK, resulting in 173 × 130 pixel‐sized images). Imaging was performed at 1.66 Hz for 9–12 min, depending on the GC protocol. Excitation light of 470 nm was either from a monochromator (Polychrome V, TILL Photonics, Germany) or from an LED source (CoolLED, 470 nm, UK) and was directed onto the antenna via a 500 nm low‐pass filter and a 495 nm dichroic mirror. Emission light was filtered through a 505 nm high‐pass emission filter. All recordings were performed between April 2017 and May 2022.

For each preparation, a reference odorant was delivered, and if the preparation gave a calcium signal, an automated protocol for a dose–response curve was run, ending with a repeated reference odorant stimulation to test that the preparation was still viable. Only blocks with equal response to initial and final reference odorant were used for analysis.

### 2.5 Data Analysis

Custom‐made Python scripts were used for data analysis. The Python‐based pyVIEW package was used for visualizing calcium imaging data (Kumaraswamy, Raiser, and Galizia 2023). Baseline drift in raw fluorescence signal caused by dye bleaching was corrected by fitting an exponential decay function of the form A * e ^−*x*/*B*^ + *C* (Galizia and Vetter 2004). Relative fluorescence change was calculated as *∆F*/*F* = (*F*_*i*_ − *F*_0_)/*F*_0_ with *F*_*i*_ being the fluorescence at frame *i* and *F*_*0*_ being the mean fluorescence of frames 10–100 (6–60s after measurement start).

An odorant whose response was neither too low nor too high was chosen as the reference odor for each Or line. Test stimuli were always applied between two reference odorant applications for all animals for control. For a given animal, the spatial pattern of fluorescence at peak response to the first reference odor application was thresholded to remove all values below the 60th percentile and normalized to the range 0–1 to form a weighted response mask. This mask was used to calculate response traces from *∆F*/*F* movies for all stimuli of that animal.

FID and calcium imaging data were recorded on separate computers and hence needed to be time‐synchronized by adjusting the response peak to the reference odorant in the FID and in the calcium trace. The necessary shift was then applied to all measurements of this animal.

Dedicated analysis pipelines were written in Python, building on the pyVIEW code. Specifically, each odorant was assigned an elution time stamp (different for the polar and the nonpolar column, different for carrier gas Helium or H_2_, different after column replacement). In addition to time stamps for odorants, we introduced time stamps for contaminations, that is, positions where calcium responses were occurring consistently, but no substance had been included in the odorant mix (note that for these substances, the dilution factor is unknown), and often, no FID signal was detectable at the respective time point. For each time stamp, a “chunk” was extracted to model the linear–nonlinear response. Model simulation and parameter estimation were done using custom code from pyView that uses the Python package “gekko” (Beal et al. 2018). Since some stimuli elicit responses with long decaying response tails, the decay was modeled and subtracted from the following trace. Small temporal jitter was adjusted by shifting the curve to the peak response time. Responses were classified as significant if their magnitude was 2.5 times higher than noise, as defined by random fluctuations in the calcium trace before stimulation.

## 3 Results

**FIGURE 1:**
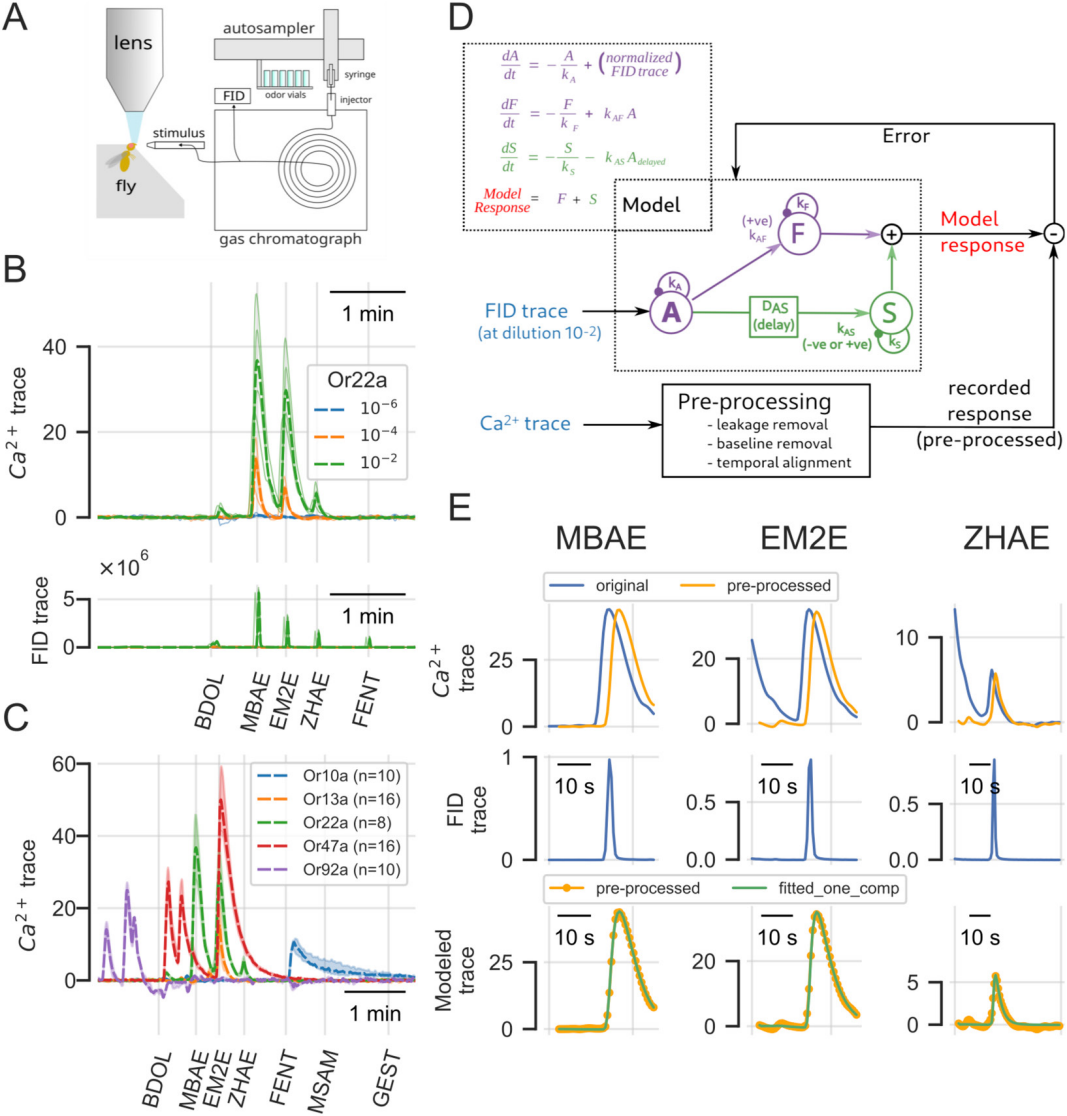
Experimental flow adopted in this paper. (A) Schematic of the setup: headspace samples are injected into a gas chromatograph using an autosampler. The eluates are split and recorded chemically with a flame ionization detector (FID) and biologically using calcium imaging on transgenic fruit flies. (B) Sample data (thin single traces and bold broken median). A mixture of chemicals was injected into the GC. These eluted at different times (FID trace), creating separate chemically clean stimuli. Early eluates generally have larger peaks in a nonpolar column, because the vapor pressure is higher, and thus, more molecules are present in the sample. A receptor (here: Or22a) responds to some of the stimuli, but not to others (Ca^2+^‐trace). Responses increase with stimulus concentration. (C) Different Or lines respond to different substances in the eluate (here all given at 10^−2^ dilution; median response traces are shown). (D) Schematic of the computational approach in our linear–nonlinear mapping. The FID trace (measured at 10^−2^ dilution) is the driving stimulus, a response (“Model response”) is created with the parameters *k*_*A*_, *k*_*AF*_, and *k*_*F*_ (for “simple” responses), and in addition, a delayed “slow” component controlled by the delay *D*_*AS*_ and the parameters *k*_*AS*_ and *k*_*S*_. The resulting calculated response is compared with the actual recorded response (preprocessed for leakage, baseline, and temporal shift), and the error signal is fed into the next iteration of a gradient descent algorithm. (E) Example of three “chunks” from a single‐animal recording in B, showing (from top to bottom) the original and the preprocessed (note how the calcium decay of the previous stimulus has been removed) Ca^2+^ trace around the response peak (the “chunk”), (middle) the corresponding FID trace, (bottom) the preprocessed response trace, and the modeled trace (fitted‐one‐comp: only the fast response parameters were necessary here). BDOL, 2,3‐butanediol; MBAE, 2‐methylbutyl acetate; EM2E, ethyl tiglate; ZHAE, Z3‐hexenyl acetate; FENT, (1R)‐(−)‐fenchone; MSAM, methyl salicylate; GEST, geranyl acetate.

In this work, we recorded calcium signals from transgenic flies that expressed a calcium sensor in all receptor cells expressing one of the following receptors: Or10a (*n* = 107 flies), Or13a (*n* = 66), Or22a (*n* = 102), Or42b (*n* = 32), Or47a (*n* = 97), Or56a (*n* = 50), and Or92a (*n* = 73). Flies were stimulated by odorants eluting from a gas chromatograph column (Figure 1A). In order to increase throughput, in most cases, we mixed five to eight odorants that eluted with a relative interval of 30 s or more. Odorants were solved in mineral oil at a dilution (concentration in liquid, v/v) of 10^−6^, 10^−4^, or 10^−2^ for each component, and concentrations were presented in ascending order. We injected 1 mL of headspace into the GC column. Highly volatile compounds have more molecules in the headspace than molecules with low volatility, therefore, the different stimuli differ in stimulus concentration even for the same dilution, and FID peaks differ in size (Figure 1B). The situation mimics the ecological situation of a fly hovering above a substrate, for example, a plant or a fruit: substances at equal concentration in the substrate will still have different molecule densities in the airborne state.

### 3.1 GC‐Purified Odorants Elicit Calcium Responses in Olfactory Receptor Neurons

Olfactory receptor neurons reacted with calcium concentration increases of varying size, depending on the molecule eluting from the GC column. For example, in Figure 1B, Or22a gave a strong response to MBAE (2‐methylbutyl acetate), EM2E (ethyl tiglate), and ZHAE (Z3‐hexenyl acetate), but no response to, for example, FENT ((1R)‐(−)‐Fenchone) or MSAM (methyl salicylate; Figure 1C). With decreasing concentration of the odor stimuli (increasing dilution, 10^−4^ and 10^−6^), responses diminished, and FID traces did not show any signal any more (Figure 1B, lower chart). Strong responses had a prolonged calcium concentration decrease tail (e.g., response to 2‐methylbutyl acetate at 10^−2^ in Figure 1B), which sometimes persisted at the occurrence of the next stimulus. Thus, for example, the response to ethyl tiglate was riding on top of the decay to 2‐methylbutyl acetate.

To the same odor mix, the other Or lines showed different response spectra (Figure 1C). Examples of full runs are given in Figures S1–S2 and S4–S6.

### 3.2 Response Modeling of Odorant–Ligand Interactions

In order to quantify the calcium responses, and to model the underlying calcium influx mechanisms, we created a linear–nonlinear model for the calcium responses, adopted from Kato et al. (2014), as shown in Figure 1D. This model considers the time course of the FID eluate and models the response as a sequence of differential equations. Briefly, *k*_*A*_ influences the initial upstroke speed of the response, k_AF_ is the main factor controlling the response size, and k_F_ governs the decay time constant of the calcium signal. These three (*k*_*A*_, *k*_*AF*_, *k*_*F*_) could correspond to the action of a single olfactory receptor complex, which opens for calcium upon binding the olfactory ligand or which activates a single second messenger cascade that opens a calcium channel: the initial, “fast” response F. In preliminary runs, we found that not all responses can be modeled with this simple mechanism. Therefore, we added a second component, *k*_*AS*_ with its own decay time constant controlled by *k*_*A*_, and with the possibility of a delay *D*_*AS*_ for its onset: the delayed “slow” response S (Figure 1D). The model gives rise to a set of differential equations that we fitted in a cascade model, using quadratic approximation (see Section 2 for details).

**FIGURE 2:**
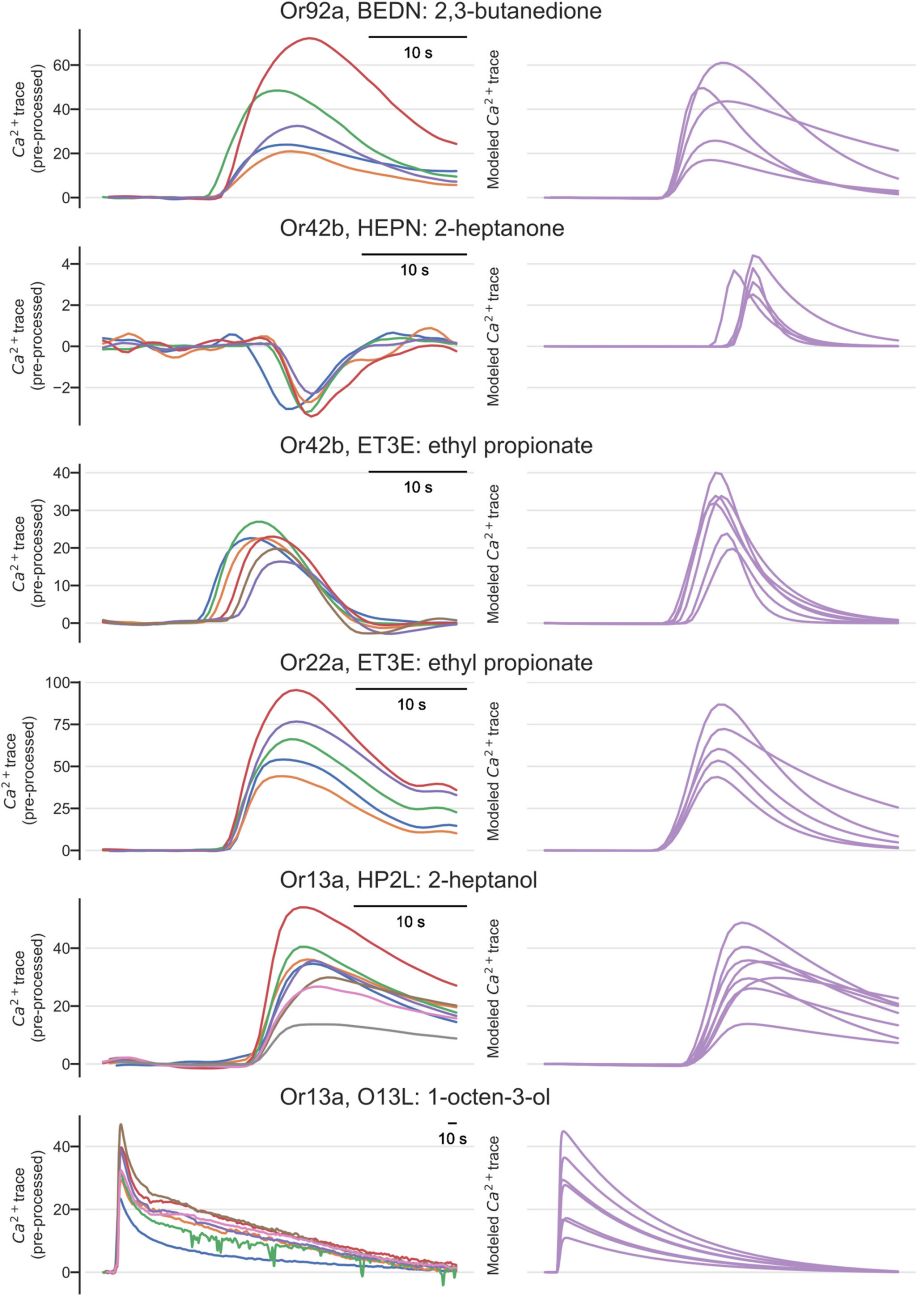
Example traces for “simple” responses. The left column shows single‐animal calcium response traces to the indicated odorant in the respective Or line, preprocessed to remove residual decaying calcium from previous responses (see Figure 1). The right column shows the corresponding modeled response. Negative responses (here to 2‐heptanone in Or42b) are first inverted and then modeled as positive responses. Here, we show the (negative) original response and the (positive) mathematical fitting.

Since sometimes responses were riding “on top of” previous responses, we used the model results to subtract the fitted decay function (“leakage removal” in Figure 1E). We then shifted the baseline (“baseline removal”) and adjusted the peaks of the responses (“temporal alignment”). The best parameters were evaluated by gradient descent. Whether the S response was necessary for explaining the response was evaluated by quantifying a Bayesian information criterion (BIC) (Schwarz 1978). Figure 1E shows three examples of the original calcium response trace, the corresponding FID trace, the preprocessed calcium trace (yellow line), and the modeled response (green trace). More examples, showing variability across animals, are given in Figure 2. Note that for modeling negative responses, like the response to 2‐heptanone in Or42b, we inverted the time course first and then applied the same algorithm (Figure 2).

### 3.3 Few Odorant‐Responses Need Two Components for Modeling

For all responses in Or10a, Or13a, Or22a, Or47a, and Or56a, fitting the single F response gave a better value for the BIC, indicating that adding the S response does not increase fitting quality. Exceptions included a few cases where the delayed slow response had the same polarity as the initial fast response and was necessary to model a slow return to calcium baseline at high concentrations. However, for Or42b and Or92a, both S and F were needed in several (but not all) responses, with opposing polarity, indicating temporally complex responses (see below).

### 3.4 Chiral Properties Can Influence Responses

**FIGURE 3:**
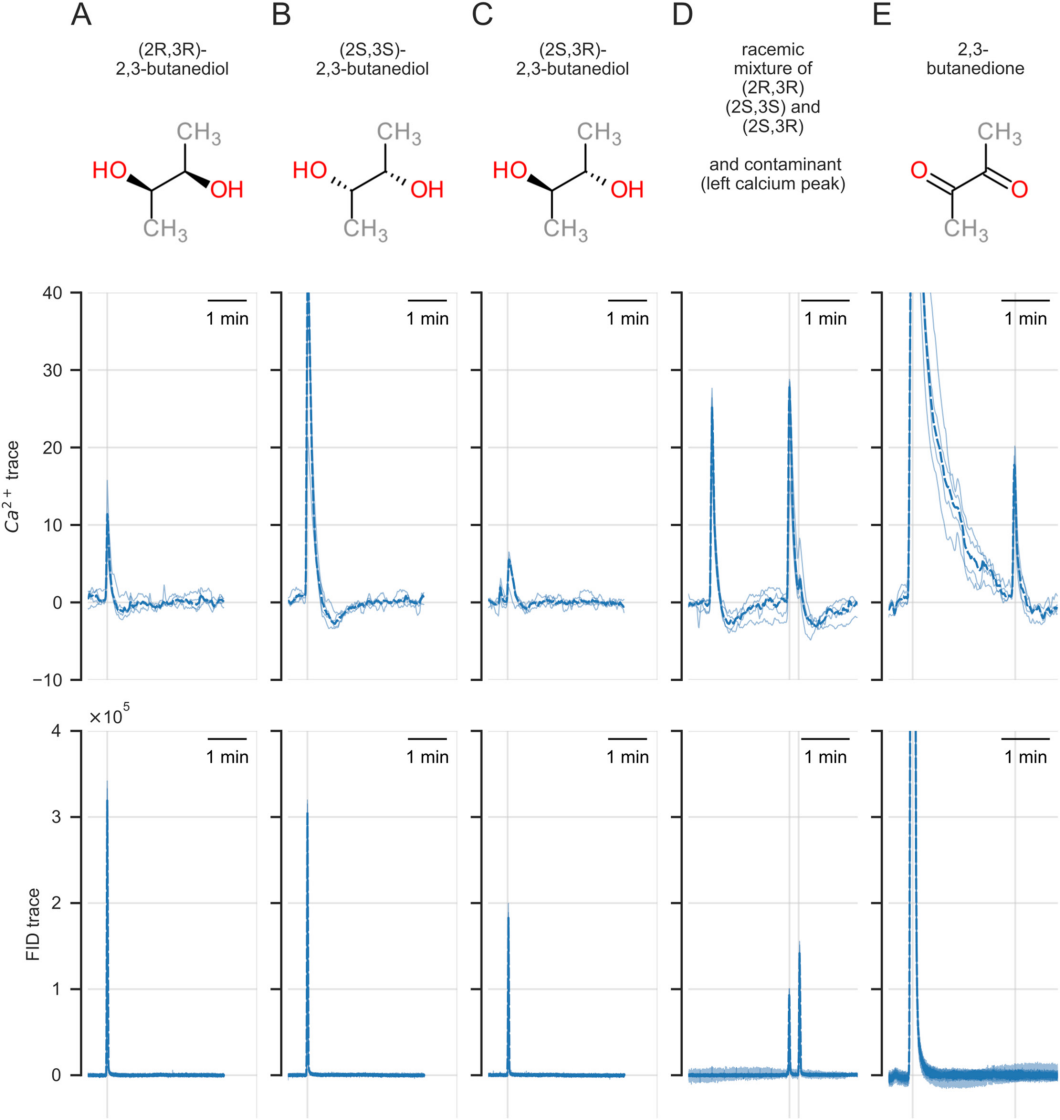
Or92a responds differently to 2,3‐butanediol isomers. Top row, chemical images of the chiral arrangements; middle row, calcium response traces; bottom row, FID response traces. Note that the different chiral substances have different concentrations in the FID trace and that the response magnitudes differ. All responses are to the eluates of the polar column; the time axis is shifted in panels D,E. (A) 2R,3R led to a response of approx. 15% (*n* = 4). (B) 2S,3S led to a response above 40%, even though the stimulus was slightly weaker (lower peak in the FID trace as compared with A) (*n* = 4). (C) 2S,3R (the meso isomer, identical to 2R,3S) gave a weak response only, preceded by a response to an unknown contamination which may be 2R,3R and/or 2S,3S, since the elution time matches (*n* = 4). (D) Response to the racemic mixture of the three isomers of butanediol. The polar column did not separate 2R,3R from 2S,3S: together, they form the first peak in the FID trace. The second FID peak is 2S,3R (meso‐isomer). Note that the calcium response is stronger to the first (smaller) FID peak, and weaker to the second (larger) FID peak, indicating that the meso form has weaker potency. The response at the beginning of the calcium trace is a response to an unknown contaminant probably not related to butanediol and not visible in the FID trace (*n* = 5). We cannot exclude the possibility that this contamination could be acetoin (3‐hydroxybutan‐2‐diol), a molecule somewhat between butanediol and butanedione. (E) Response to the best‐known ligand for this receptor, butanedione (first peak), with the response to a contaminant in the tail, which is the same contaminant as in D (*n* = 4), only apparently shifted here because another time window is shown. The four animals measured in A were also measured in B, C, and D. Compare with Figure S3.

The best ligand for Or92a was diacetyl (2,3‐butanedione). However, we also noted a strong response to 2,3‐butanediol. This molecule is sterically somewhat similar to diacetyl: four aliphatic carbon atoms, however with alcohol groups at Atoms 2 and 3 instead of a ketone (Figure 3). Unlike diacetyl, 2,3‐butanediol can take three different steric conformations: the chiral pair 2S,3S and 2R,3R or the meso‐isomer 2S,3R (which is equivalent to 3S,2R). All three forms are produced by a variety of microorganisms (Ji, Huang, and Ouyang 2011). We found that all three isomers elicited strong biphasic responses in Or92a: first a calcium increase, then a decrease. In all cases, the model gave a significant contribution of the S response component (Figure S3). The meso form gave only weak responses, and among the two chiral forms, 2S,3S gave the stronger response (Figures 3 and S3). We hypothesize that the 2S,3S form has the highest steric compatibility with the binding pocket optimized for diacetyl.

### 3.5 Odor‐Response Patterns Differ Across Receptor Lines

**FIGURE 4:**
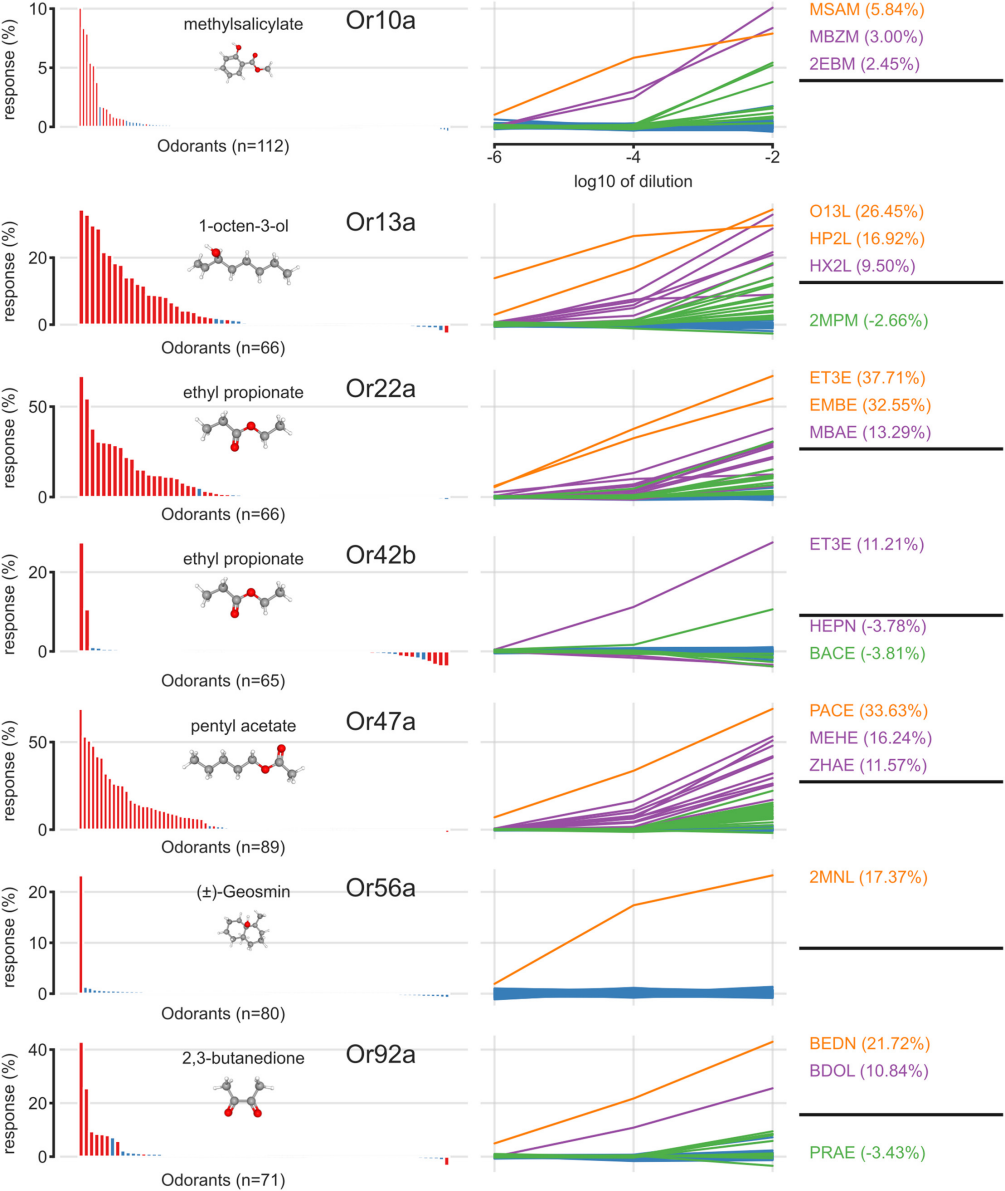
Response patterns across odorants for the seven Or lines considered. Left column: histogram of response magnitude (ordinate) at a dilution of 10^−2^. The abscissa represents the different odorants tested in each line (*n* refers to the number of odorants tested); each bar of the histogram is the median modeled response across all measurements. Odorants are sorted within each Or line: strongest responses to the left, weakest responses, and negative responses to the right (see Table S2 for odorants and response values). Red columns: responses that are statistically significantly different from preceding noise level. The best ligand at 10^−4^ dilution is shown as chemical sketch for each receptor. Right column: response magnitudes (ordinate) to the full dose–response curve at dilutions 10^−6^, 10^−4^, and 10^−2^ (abscissa is odorant dilution). Colors indicate if a substance has a statistically significant response at all three dilutions (orange), only at the top two (purple), only at 10^−2^ (green), or at none (blue). Above the black line, we name the three best ligands, below up to the two strongest negative ligands (if present). 2EBM, ethyl benzoate; 2MNL, (±)‐geosmin; 2MPM, 2‐methylphenol; BACE, butyl acetate; BDOL, 2,3‐butanediol; BEDN, 2,3‐butanedione; EMBE, ethyl 2‐methylbutanoate; ET3E, ethyl propionate; HEPN, 2‐heptanone; HP2L, 2‐heptanol; HX2L, (±)‐2‐hexanol (rac); MBAE, 2‐methylbutyl acetate; MBZM, methyl benzoate; MEHE, methyl hexanoate; MSAM, methyl salicylate; O13L, 1‐octen‐3‐ol; PACE, pentyl acetate; PRAE, propyl acetate; ZHAE, Z3‐hexenyl acetate.

We found both significant calcium increases and calcium decreases. Responses were defined as significant within a recording if their values were significantly above 2.5 SD of the preceding noise in calcium fluctuations (one‐sided paired *t*‐test, *p* < 0.01). The relative proportion of significant responses differed consistently across receptors, reflecting the differences in response kurtosis (Münch and Galizia 2016). Or56a, with only one responsive ligand (geosmin), was the most selective profile in our sample, whereas Or13a and Or22a were the most “promiscuous” receptors (Figure 4, left column). Significantly negative responses were rare: most were recorded in Or42b, and some in Or92a. Overall, negative responses always had a small magnitude, reflecting the fact that baseline calcium concentrations in the cells are already low and cannot be decreased much more by closing an olfactory receptor upon ligand binding.

Next, we analyzed response strengths across odorant concentrations. Generally, at 10^−6^ dilution, only a few substances elicited significant responses: methyl salicylate in Or10a; 2‐heptanol, 1‐octen‐3‐ol in Or13a; ethyl propionate and ethyl‐2‐methylbutanoate in Or22a; pentyl acetate in Or47a; geosmin in Or56a; and 2,3‐butanedione in Or92a. With increasing ligand density (concentration), the number of effective substances increased (Figure 4, right column); in several cases, the ligand that responded at very low concentrations already saturated at 10^−2^, with the effect that another ligand elicited the strongest response. In other words, the sequence of best to intermediate ligand at a concentration of 10^−2^ was different from the sequence of best to intermediate ligand at a concentration of 10^−4^. For example, 1‐octen‐3‐ol was clearly the best ligand for Or13a at low and very low concentrations (see below), but at 10^−2^, this substance saturated the receptor, leading to diminished responses, whereas 2‐heptanol at 10^−2^ gave stronger responses.

#### 3.5.1 Odor‐Response Patterns in Individual Lines: Or10a

This receptor showed a fairly selective response pattern: out of 112 tested odorants, only 14 substances gave a significant response at the highest concentration of 10^−2^ dilution (category number > 0 in Table S2; *n* = 107 animals measured). The best ligand was methyl salicylate, and good ligands were methyl benzoate and ethyl benzoate (Figure 4). The other substances giving significant responses were butyl acetate, isobutyl acetate, acetophenone (1‐phenylethanone), isoamyl acetate, ethyl tiglate, benzonitrile, benzaldehyde, anisole (methoxybenzene), trans‐2‐hexen‐1‐ol, ethyl acetate, and 2‐hexanone. Most were esters (not acetophenone, benzonitrile, benzaldehyde, anisole, trans‐2‐hexen‐1‐ol, and 2‐hexanone), seven were aromatic molecules with a benzene ring (methyl salicylate, methyl benzoate, ethyl benzoate, acetophenone, benzonitrile, benzaldehyde, and anisole), whereas seven were aliphatic molecules (butyl acetate, isobutyl acetate, ethyl tiglate, isoamyl acetate, trans‐2‐hexen‐1‐ol, ethyl acetate, and 2‐hexanone). Most have a fruity smell to the human nose. Molecular weight ranged between 88 and 152 g/mol, with four to nine carbon atoms. We did not find any negative responses, and all positive responses with the exception of the strongest response to methyl salicylate could be modeled with a single function (response “F”), suggesting a single transduction mechanism for all odorants tested. The response to the strongest concentration of methyl salicylate was more complex, with a strong initial upstroke and a fast initial decay, followed by a slower decay, indicating a fast initial adaptation in the response: in our 2‐function model of F and S response, a long‐lasting S response was necessary to fit the shape, and the initial short and strong response was not covered entirely in the modeled response. As a result, methyl salicylate appears less strong a ligand at 10^−2^ than ethyl benzoate (Figure 4).

#### 3.5.2 Odor‐Response Patterns in Individual Lines: Or13a

This receptor was characterized for 66 odorants (Figure 4) and measured in *n* = 66 animals. Or13a showed very strong responses to 1‐octen‐3‐ol, with long‐tailed calcium influx (see Sections 3.8 and 3.9), and strong signs of saturation at high concentration. Significant responses down to a dilution of 10^−6^ were also present to 2‐heptanol, which as compared with 1‐octen‐3‐ol differs in that it misses one double‐bond carbon atom at the short side of the aliphatic molecule. Medium responses (i.e., significant to dilution of 10^−2^ and 10^−4^, category no. 2 in Table S2) were recorded to seven substances: 2‐hexanol (racemic mixture), 1‐hexen‐3‐ol, ethyl(S)‐(+)‐3‐hydroxybutyrate, 1‐hexanol, Z3‐hexen‐1‐ol, hexyl acetate, and 3‐octanol (Figure 4). All molecules, with two exceptions (ethyl(S)‐(+)‐hydroxybutyrate and hexyl acetate, which are both esters), were alcohols, all aliphatic hydrocarbons with six to eight carbons, and molecular weight between 100 and 144 g/mol. To the human nose, the best ligands smell “green,” or like cut grass, and also, the medium ligands were typical green plant components with a fresh smell to humans. Several (16) of the tested substances gave weak responses (significant only to the highest concentration, dilution 10^−2^), whereas 40 substances gave no response. We found one substance that gave a negative response (calcium decrease): 2‐methylphenol (2MPM). In total, 26 out of 66 tested substances (39%) gave a response at the highest concentration (10^−2^), showing a fairly broad odor‐response profile (Figure 4). All responses could be modeled with a single function (response “F”), suggesting a single transduction mechanism for all odorants tested. Exceptions were strong responses to 1‐octen‐3‐ol, where the slow component was needed to model a slow decay in the response.

#### 3.5.3 Odor‐Response Patterns in Individual Lines: Or22a

In Or22a, we tested 66 substances in a total of *n* = 102 animals. Two odorants (ethyl propionate and ethyl‐2‐methylbutanoate) gave significant responses already at 10^−6^ dilution, 10 odorants at 10^−4^ and higher (2‐methylbutylacetate, ethyl tiglate, butyl acetate, ß‐butyrolactone, heptanal, methyl‐3‐hydroxy hexanoate, 3‐hexanol, ethyl hexanoate, 3‐octanone, and 2‐heptanol), and 14 more gave responses at 10^−2^ only. Forty substances gave no significant response across the tested dilutions. Most ligands were esters, with five to eight carbon atoms, and molecular weight in the range of 100–130 g/mol, with some outliers beyond this range. To the human nose, most of these substances smell fruity and are reminiscent of berries or other fruit or fruity fermentation products. Overall, this is a receptor with a broad response profile. Isoamyl acetate, which is often reported as a good ligand, gave a strong response at 10^−2^ (fourth best ligand), but weaker responses at lower concentrations (ranked 7th best at 10^−4^ and 65th at 10^−6^). All responses could be modeled with a single function (response “F”), suggesting a single transduction mechanism for all odorants tested. No negative responses were found for any tested odorant. There was one special case that we observed but did not investigate further in this study: propanoic acid consistently elicited an “off” response, that is, a positive calcium increase at the end of the stimulus, without an inhibitory “on” response (see Figure S5). More experiments will be needed to characterize this specific observation.

#### 3.5.4 Odor‐Response Patterns in Individual Lines: Or42b

In Or42b, we tested 65 substances in a total of *n* = 32 animals. We found strong responses to ethyl propionate (positive), 2‐heptanone (negative), and 2‐heptanole (negative), which were significant at dilutions of 10^−4^ and 10^−2^. Positive responses were also found to ethyl (S)‐(+)‐3‐hydroxybutyrate (significant only at 10^−2^). Negative responses significant at 10^−2^ were found to butyl acetate, ethyl tiglate, isoamyl acetate, methyl‐3‐hydroxyhexanoate, 2‐octanone, and ß‐ionone. All molecules were aliphatic hydrocarbons ranging from a C5 to a C8 backbone and molecular weight between 102 and 146 g/mol (except ß‐ionone C13, 192 g/mol). All inhibitory substances were also aliphatic compounds of similar size as the excitatory ligands, suggesting some fine‐scaled mechanism in the binding pocket. This hypothesis is corroborated by the observation that several substances led to biphasic responses: for example, ethyl propionate led to a strong positive response, followed by a calcium decrease below baseline, as did ethyl‐(S)‐(+)‐3‐hydroxybutyrate. Conversely, propanoic acid led to a negative response, followed by a positive rebound above baseline. In all these cases, the linear–nonlinear model needed both the fast and the delayed slow component to model the odorant responses. We note, however, that the responses recorded with our stimulus sample never reached significant magnitude at 10^−6^ dilution, suggesting that we are possibly still ignorant about a stronger or ecologically more important ligand. This observation is strengthened by our observation that within the GC‐run of ethyl propionate, we could consistently see a strong response to a contamination: ethyl propionate (ET3E) eluted at minute 4.51, whereas the contamination (ET3EXX in Figure S6) eluted at 4.11. The response to the contamination was about half the response to ethyl propionate, but totally invisible in the FID trace, indicating that at this location, a very strong ligand had eluted. Future experiments will be needed to identify this substance.

#### 3.5.5 Odor‐Response Patterns in Individual Lines: Or47a

In Or47a, we tested 89 substances in a total of *n* = 97 animals. We found strong responses to pentyl acetate (significant to very low concentrations, see below) and medium responses (i.e., significant to dilutions of 10^−4^ and 10^−2^, but not to 10^−6^) to the following 11 substances: methyl hexanoate, propyl acetate, Z3‐hexenyl acetate, butyl acetate, isobutyl acetate, methyl‐3‐hydroxyhexanoate, ethyl‐3‐methyl‐sulfanylpropanoate, hexyl acetate, 2‐methyl butyl acetate, 2‐heptanone, and isoamyl acetate. Twenty‐one additional substances gave responses at high concentrations. The resulting odor‐response profile was fairly broad (Figure 4). Many of the best ligands smell to the human nose somewhat fruity and are present in pears, apples, and similar fruits. Almost all were esters, with five to eight carbon atoms and molecular weight between 102 and 148 g/mol. Ethyl‐3‐methyl‐sulfanylpropanoate is a sulfur‐containing substance, but it should be noted that very few such molecules were in our test battery; therefore, we cannot extrapolate to other sulfur‐containing molecules. All responses in Or47a were positive and could be fitted with a single “F” response, with the exception of responses to pentyl acetate at 10^−2^ and 10^−4^ and to 3‐octanol at 10^−2^. Here, the fitting procedure added a positive slow component, in order to model a slower decay, that is, to maintain the calcium concentration for a longer period. No negative responses were measured in this line.

#### 3.5.6 Odor‐Response Patterns in Individual Lines: Or56a

In Or56a, we tested 80 substances in a total of *n* = 50 animals. We found strong responses to geosmin and none to any of the other substances. Responses to geosmin were strong down to 10^−6^; however, at further dilutions, the responses were not dose‐dependent any more, suggesting that they were due to contamination with geosmin in our syringe. All responses were positive, with a strong response upstroke and a long‐lasting decay, without any temporal complexity in the response. All responses could be modeled with a single function (response “F”), suggesting a single transduction mechanism for this highly selective odorant receptor.

#### 3.5.7 Odor‐Response Patterns in Individual Lines: Or92a

In Or92a, we tested 71 substances in a total of *n* = 73 animals. We found the strongest responses to 2,3‐butanedione (diacetyl): a single upstroke and decay positive response, with no temporal complexity. The next best odorant was 2,3‐butanediol, where the two ketone groups of diacetyl are replaced by two alcohol groups. The temporally complex responses to these chiral substances are reported above (Figure 3).

Several substances elicited biphasic signals, with calcium increase followed by a decrease below baseline: 3‐hexanol (H3XL), 3‐hexanone (3HXN), 2,3‐butanediol (BDOL), ethyl (R)‐(−)‐3‐hydroxybutanoate (ERHE), and ethyl (S)‐(+)‐3‐hydroxybutyrate (ESHE). Negative responses were elicited by propyl acetate (PRAE), eliciting a strong calcium decrease upon stimulation, with a calcium influx as a rebound response after stimulus offset. Modeling of this response required both the “F” and the “S” components. “Complex” negative responses were also found to FURL (furfural). “Simple” negative responses without rebound were found to ethyl 4‐oxoperitanoate (EOPE), methyl tiglate (MTIE), 3‐hexanol (H3XL), isoamyl acetate (ISOE), and 2‐heptanone (HEPN). “Simple” positive responses were found to ET3E. Thus, Or92a is a complex olfactory receptor, responding to many substances (broad response profile), with temporally complex responses (some first positive, then with a negative late phase, some first negative, then with a positive late phase), but also with uniform responses that were not biphasic (such as to the best ligand diacetyl).

It should be noted, though, that not all of the above‐mentioned ligands are visible in Figure 4: the reported qualitative response spectrum is in part the result of visual inspection of the response traces, and not of our linear–nonlinear modeling. The reason for this is that Or92a was a receptor line with a high level of background calcium activity, that is, of background noise—and since our analysis pipeline excludes signals below a certain noise threshold, responses in Or92a were often excluded from the standardized analysis. It may well be that elevated background activity correlates with elevated intracellular calcium levels, allowing for more visible negative responses, as compared with receptor cells with low baseline calcium concentration. Nevertheless, rather than adjusting the parameters, we opted for a visual inspection of the traces and a qualitative report as done here. Furthermore, this line often gave responses to unknown substances (contaminations): this receptor needs more experiments to understand its binding properties beyond the strongest ligands diacetyl (2,3‐butanedione) and 2,3‐butanediol.

### 3.6 Strong Ligands Often Elicit Long‐Tailed Responses

**FIGURE 5:**
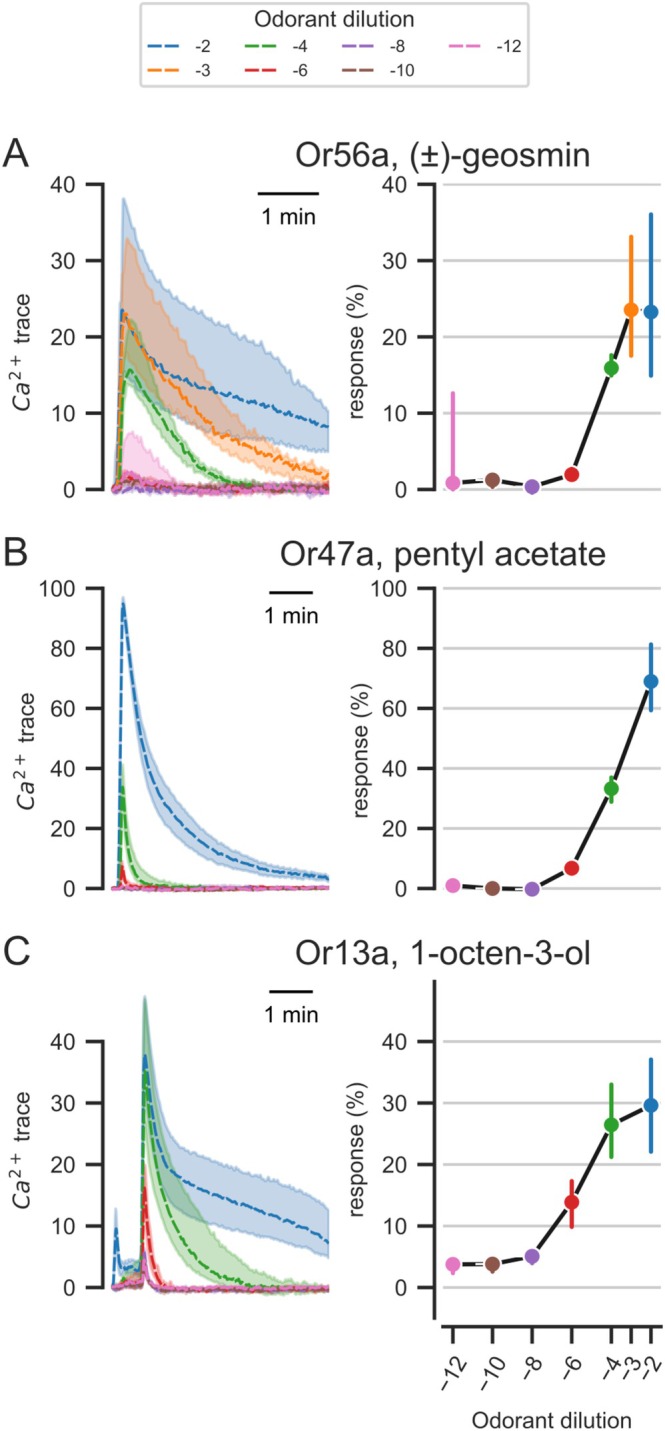
Calcium response traces to extended dose–response samples for best ligands. Left column: calcium response traces, median, and interquartile range, across concentrations. Right column: response magnitude (peak of the median response trace) across concentrations. (A) Responses to (±)‐geosmin in Or56a have an increasingly long tail as the concentration increases, but the peak response does not increase further (peak response is equal for dilution 10^−2^ and 10^−3^, right chart; *n* = 9–17). (B) Responses to pentyl acetate in Or47a do not change their temporal response properties with increasing concentration, the longer tail appears like a scaling effect. The dose–response curve (right chart) does not show signs of saturation at high concentrations. *N* = 2–4. (C) Responses to 1‐octen‐3‐ol in Or13a have an increasingly long tail as the concentration increases, but the peak response reaches saturation (peak response is approximately equal for dilution 10^−2^ and 10^−4^, right chart). *N* = 10.

Next, we analyzed the responses at different concentrations in more detail, focusing on the best ligands (Figure 5). In particular, since in the main screen, the best ligands already gave responses at a dilution of 10^−6^; in this experiment, we expanded the range down to concentrations of 10^−12^. For geosmin in Or56a, significant responses started with a dilution of 10^−6^, and increasing odorant concentration led to a steep dose–response curve, reaching close to saturation at 10^−2^. At concentrations lower than 10^−6^, we consistently found small, concentration‐independent, responses at the elution time of geosmin, indicating that this substance had contaminated the syringe (Figure 5A). Pentyl acetate in Or47a also did not give any responses below 10^−6^ but did not show any sign of saturation up to 10^−2^ (Figure 5B). The situation was similar for 1‐octen‐3‐ol in Or13a. Again, in this ligand‐receptor pair, we could not get a lower limit: although with 10^−6^, we reached the lowest response in the dose–response curve, with all further dilutions, we still saw responses that were not dose‐dependent (Figure 5C), indicating a systematic contamination in the syringe.

At low concentrations, all responses could be modeled using the single F component, whereas at high concentrations, the model became better if a second positive component was added, accounting for a prolonged response. As a consequence, even with a peak response already saturated (e.g., for 1‐octen‐3‐ol in Or13a at 10^−4^ and 10^−2^), the integral (“area‐under‐the‐curve” measure) still increased with increasing concentrations: if the central nervous system were to evaluate the integral as readout, saturation of odor–receptor responses would occur at a higher concentration as compared with peak calcium response.

### 3.7 Long‐Tailed Responses Are Both Biological and Experimental

**FIGURE 6:**
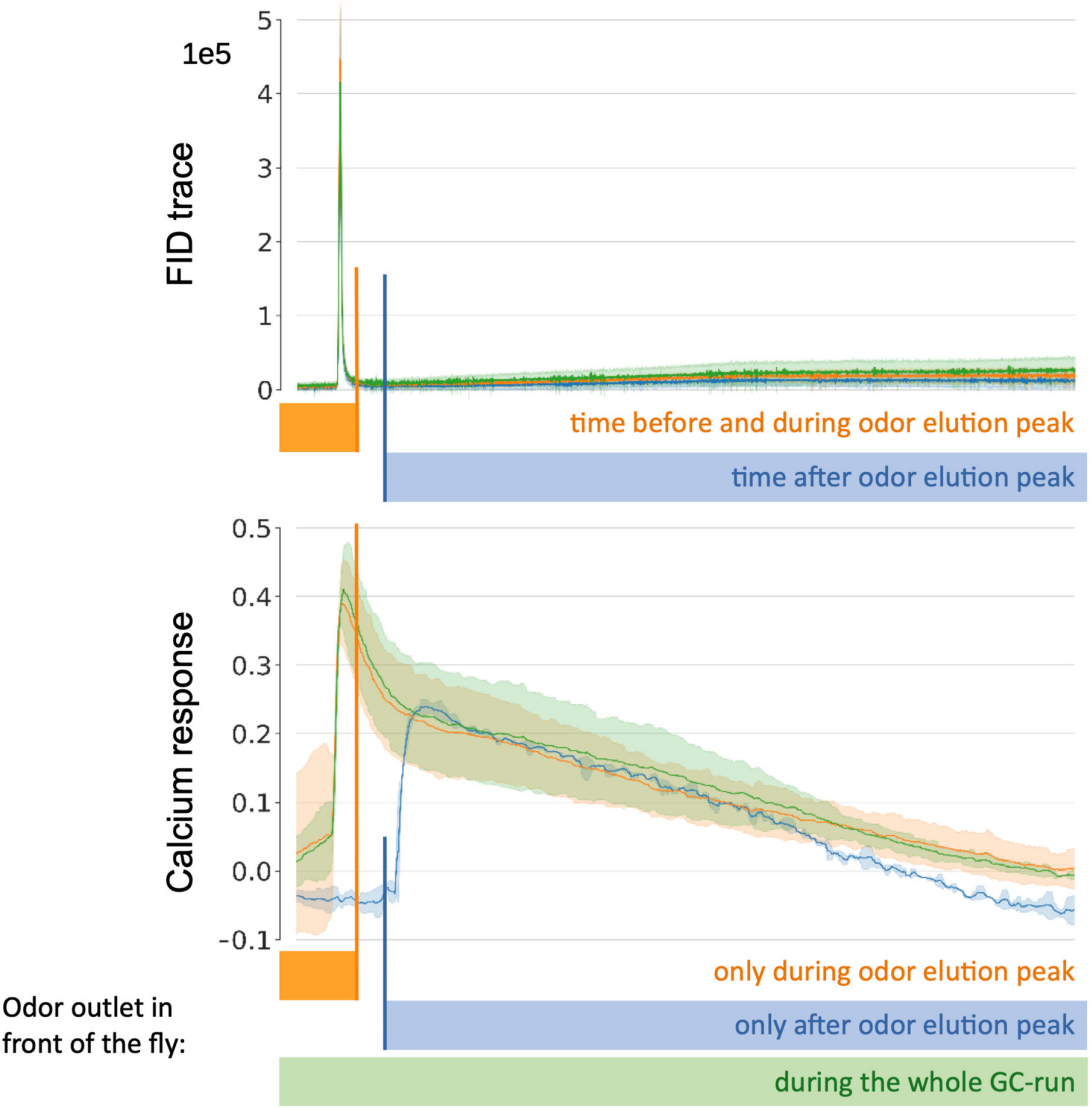
Long‐lasting tail responses are due to both biological processes and GC eluate smear. 1‐octen‐3‐ol at 10^−2^ dilution was injected into the GC, yielding a strong FID peak (top trace: median and SD). The calcium response during the whole GC‐run is given by the green trace (bottom trace: median and SD, *n* = 3). In some experiments, we shielded the antenna from the eluate right after the peak elution was over: orange trace. The response was still long‐tailed, indicating that 1‐octen‐3‐ol during the initial phase of the stimulus was sufficient to yield the full long‐tailed response. In other experiments, however, we shielded the antenna before and during the main elution peak (blue trace). No calcium response was seen at this time, indicating that the shielding was complete. However, when the shielding was removed, we saw a strong long‐tailed response, indicating that the GC column was delivering 1‐octen‐3‐ol over prolonged times after the first peak.

The long‐lasting responses to good ligands at high concentrations could, in principle, be caused by either of two independent phenomena: either the ligand binds the receptor and the receptor (or the post‐ligand‐binding mechanism) remains active for an extended period (biological reason) or the gas chromatograph column peak is not as focal as it appears to be from the FID trace but smears over time and releases molecules in a long tail (experimental artifact). In order to investigate this point, we performed a dedicated experiment: we compared responses in Or13a to three stimulus situations (Figure 6): (1) 1‐octen‐3‐ol was eluted from the GC column, as in the experiments above, and the calcium increase was measured in Or13a antennae (Figure 6, green trace); (2) the GC column outlet was shielded right after the peak release, preventing the arrival of additional molecules eluting from the GC column to the olfactory receptors (Figure 6, orange trace); or (3) the GC column was shielded at the beginning and during peak release, and only the tail elute from the GC was presented to the animal (Figure 6, blue trace). When shielding the GC elute right after the peak (orange trace), the calcium concentration in the receptor neurons remained high and decreased as if the column had not been shielded (green calcium trace). Thus, lingering calcium, or on‐going receptor machinery activity, or “sticky” ligands at the receptor lead to a long‐tailed response, irrespective of the presence of additional odorant molecules in the air at the antenna. However, when presenting the tail eluate only (blue trace), the receptors responded just as in the tail alone. This indicated that the GC column released 1‐octen‐3‐ol molecules for a long time after the peak, and these molecules were sufficient to generate a response that was comparable, in its initial part, to the full response. The “tail‐only” stimulus, however, decayed faster than the corresponding full response, and also faster than the response to the peak with later shielding (blue trace in Figure 6), as would be expected from a response at lower concentration. These results suggest that both smearing in the GC column and “sticky” receptor properties contribute to long‐tail responses at high odor concentrations.

### 3.8 Some Responses Were due to Contaminants in the Samples

**FIGURE 7:**
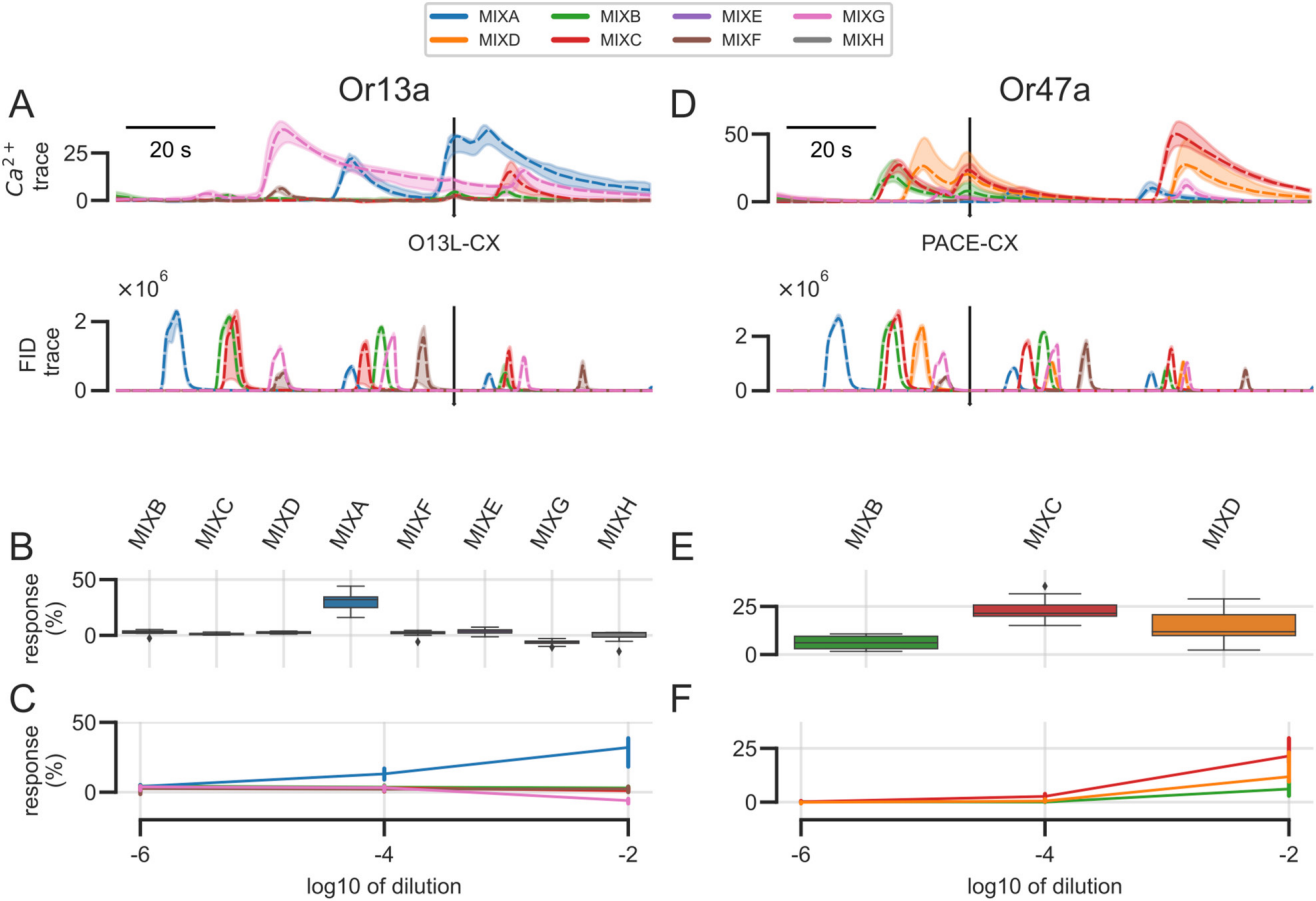
Strong ligands can elicit calcium responses, even as minute contaminations which are not detectable with the GC‐FID. (A) A strong ligand of Or13a elicited strong and weak calcium responses in different odor mixes at the elution time of 1‐octen‐3‐ol (vertical bar, O13L‐CX, top traces), revealing the presence of 1‐octen‐3‐ol, although it was not added to these mixes. No FID signal was detectable at this time point (bottom traces), indicating that it was present at very low concentration. (B) The calcium response to the 1‐octen‐3‐ol contamination was strongest in MixA, whereas the responses in most other mixes were small (the only mix with no visible response was MixG, likely because of lingering calcium response to the preceding stimulus, see the pink trace in A). (C) The response to the 1‐octen‐3‐ol contamination in MixA (blue line) was concentration dependent, indicating that the contamination originated from one of the mix components. The response in the other mixes was not concentration dependent, indicating a contamination in our experimental laboratory. (D) A strong ligand of Or47a elicited calcium responses in different odor mixes at the elution time of pentyl acetate (vertical bar, PACE‐CX) (top traces), although pentyl acetate was not added to these mixes. No FID signal was detectable at this time point (bottom traces). (E) The calcium response to the PACE contamination was strong in MixB, MixC, and MixD. (F) The response to the PACE contamination showed a concentration dependency in MixC (red line), MixD (orange line), and MixB (green line). This reveals that the contamination originated from another odorant, added to these mixes, rather than from an overall contamination within our setup.

1‐Octen‐3‐ol gave very strong responses in receptor Or13a: in the dose–response analysis, there were responses even at the lowest concentration (see above, Figure 5), suggesting a systematic contamination with 1‐octen‐3‐ol in our system. We therefore analyzed responses at the elution time of 1‐octen‐3‐ol in all mixes and indeed found a contamination in our mixture A, yielding a strong response (Figure 7A), that was concentration dependent: with increasing concentration in mixture A, the response at elution time 1‐octen‐3‐ol increased, suggesting a contamination of mixture A with traces of this substances (Figure 7C). Note that no signal could be detected in the chemical analysis, that is, in the FID trace, at that time point: the concentration was below the FID detection limit. However, we also found (small) responses at the elution time of 1‐octen‐3‐ol in most other mixes (Figure 7B). Interestingly, these responses were not concentration dependent, suggesting that the contamination was not introduced by the stimulus substances but was present either in the solvent or in the air or attached to the syringe (Figure 7C). It should be noted that 1‐octen‐3‐ol is a common substance in human skin and many cosmetic products and is ubiquitous in a laboratory environment at subthreshold levels. In our hands, the amount of 1‐octen‐3‐ol in mixture A, and also in the contamination of the other mixtures or the lab environment, was below the detection threshold for our GC‐FID but clearly above the threshold for Or13a on the fly antenna.

When responses to a contaminant increase with concentration, it shows that the contaminant was present in one of the substances used for that odorant mix. Or47a responded to a contaminant in Mixtures B and C in a concentration‐dependent manner (Figure 7D–F). We found that this response was at the elution time of pentyl acetate and was masked by the addition of pentyl acetate, suggesting that traces of pentyl acetate must be in (at least) one of the components of these mixtures.

Responses to mixture P in Or47a also contained a double peak of responses, in a concentration‐dependent manner, at a location in the elute where none of the substances that we had added was eluting (data not shown). We tested each component of the mixture individually and found that these contaminations came from the commercially available 3‐methylthio‐1‐propanol (MTPL, CAS 505‐10‐2). In our hands, the amount of the contaminant was below the detection threshold for our GC‐FID and also for the GC–MS but clearly above the threshold for Or47a on the fly antenna. As a first guess, we tested several closely related substances (3‐methyl‐1‐propanol [MOPL, CAS 1589‐49‐7], 3‐methylamino‐1‐propanol [MAPL, CAS 42055‐15‐2], 2‐ethylmercapto‐ethanol [EMEL, CAS 110‐77‐0], 3‐ethylsulfanyl‐1‐propanol [ESPL, CAS 18721‐61‐4], and 4‐methylthio‐1‐butanol [MTBL, CAS 20582‐85‐8]) and could show that none of these eluted at the same time as the contaminant giving the best response. Thus, we did as yet not find the identity of the contaminant, which may well be a chemical that is not closely related to 3‐methylthio‐1‐propanol. More experiments will be needed.

### 3.9 Comparison With the DoOR Database

**FIGURE 8:**
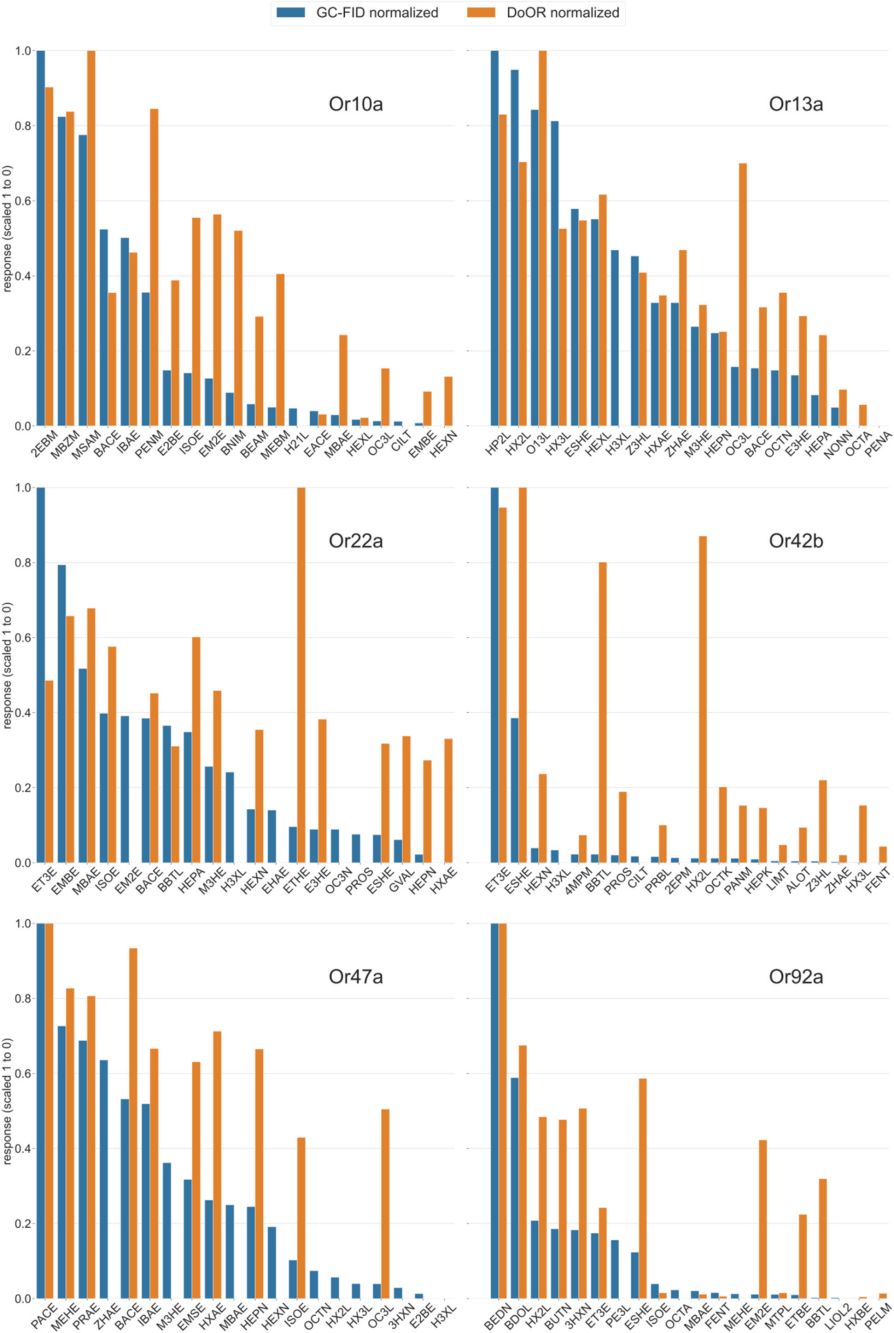
Comparison of our responses with the DoOR database. The 20 strongest responses in our screen were selected and scaled from 1 to 0, for each Or line independently (blue bars). For the same odorants, the responses in DoOR were downloaded from https://neuro.uni‐konstanz.de/door and scaled in the same way (orange bars). Some ligands were not present in the DoOR database. Note that there is good agreement between the two datasets for strong responses, but for weaker ligands, DoOR generally overestimated the response, indicating false positives in the database. Notable examples are 3‐octanol (OC3L) in Or13a, ethyl hexanoate (ETHE) in Or22a, or ethyl tiglate (EM2E) in Or92a. Or56 is not shown since it does not have a response spectrum, given that only a single ligand is known. See Table S1 for legend of all odorant abbreviations. Top three for each line are Or10a: 2EBM (ethyl benzoate), MBZM (methyl benzoate), MSAM (methyl salicylate); Or13a: HP2L (2‐heptanol), HX2L (2‐hexanol), O13L (1‐octen‐3‐ol); Or22a: ET3E (ethyl propionate), EMBE (ethyl 2‐methylbutanoate), MBAE (2‐methylbutyl acetate); Or42b: ET3E (ethyl propionate), ESHE (ethyl (S)‐(+)‐3‐hydroxybutyrate), HEXN (2‐hexanone); Or47a: PACE (pentyl acetate), MEHE (methyl hexanoate), PRAE (propyl acetate); Or92a: BEDN (2,3‐butanedione), BDOL (2,3‐butanediol), HX2L (2‐hexanol). Or56a not shown (only ligand: 2MNL, geosmin).

The DoOR database has collected odor‐response profiles in *Drosophila* from the literature and mapped these responses onto a standardized scale irrespective of the technique used to measure the responses (Münch and Galizia 2016). We have compared the odor‐response profiles as reported here to the DoOR response profiles (Figure 8). Since the DoOR database does not contain information from dose–response curves, we used the responses to 10^−2^ dilution. We selected the 20 best‐responding substances in our panel and scaled them from 1 to 0. Similarly, we downloaded the responses to these substances from the DoOR database and scaled them from 1 to 0. The inspection of the resulting two histograms is informative: in Figure 8, the orange bars represent the DoOR responses. It is apparent that for strong ligands, there is a good match, in most cases, between our new data reported here and the DoOR responses. With decreasing response strengths, however, DoOR responses often appear to be larger. This suggests that, when odorants are purified with a GC right before stimulation, false positives may be reduced. Apart from the innocuous general trend (several substances giving stronger responses), we found a few prominent outliers that need special mentioning: the response to 3‐octanol (OC3L) in Or13a is highly overestimated in the DoOR database. Indeed, in our data, we found that 3‐octanol was systematically contaminated with 1‐octen‐3‐ol, creating false‐positive recordings (Figure 7). We found the response to ethyl 3‐hydroxyhexanoate (ETHE) in Or22a to be highly overestimated in the DoOR database. β‐Butyrolactone (BBTL) and (±)‐2‐Hexanol (rac) (HX2L) were highly overestimated for Or42b in the DoOR database: our purified data would suggest that this must be due to a contamination. Similarly, 3‐(methylthio)‐1‐propanol (MTPL) is not a strong ligand for Or47a (ranked 23rd in Table S2; not shown in Figure 8), even though the DoOR database lists it as one: as noted above, we found that commercial 3‐(methylthio)‐1‐propanol contains two contaminants with high potency for Or47a. Finally, we did not find strong responses to ethyl (S)‐(+)‐3‐hydroxybutyrate (ESHE) in Or92a, which is reported as a good ligand in the DoOR database.

We confirm that Or56a is a geosmin receptor: however, in the DoOR database that ligand did not appear, due to the mathematical formula used in DoOR to create consensus response profiles: with a single ligand, and no other significant responses, the algorithm behind the DoOR database is not capable of creating a response profile.

In this study, we recorded responses to odorants that have been purified on the spot by passing a GC column. As a consequence, we have identified a few false positives in the DoOR database. Interestingly, however, the effect is relevant for individual substances, but not for the overall picture. For example, Or47a remains a receptor that responds to many substances, even though it is highly sensitive to pentyl acetate, and also, Or13a remains a receptor with a broad response profile, even though 1‐octen‐3‐ol (O13L) is a highly efficient ligand.

## 4 Discussion

We characterized odor‐response profiles for seven *Drosophila* olfactory receptor cell types, using pure monomolecular substances by stimulating with the eluate of a gas chromatograph. We found that basic properties of olfactory coding vary in significant ways across receptors, including in terms of selectivity (responding to many or few substances), response polarity (responding with calcium increases only, or also with calcium decreases), and temporal properties (responding with complex time courses or “simple” time courses). Specifically, Or56 is a very selective receptor for geosmin, with a positive response without any temporal complexity and long‐lasting responses at higher concentrations. Similarly, Or10a is fairly selective (few ligands) with only positive responses, long‐lasting at higher concentrations. Or47a and Or22a are broadly responding receptors (many ligands) with always positive, temporally simple responses. Or13a shows positive responses (calcium increases) and one negative response, always with a simple time course, and long‐lasting responses for high‐concentration stimuli. Or42b and Or92a are quite different: both have broad response spectra (many ligands), positive responses to some and negative responses to other ligands, and complex time courses for some ligands, generally of a biphasic nature: a positive response would end (after stimulus termination) with a calcium decrease below baseline, or a negative response would end with a calcium increase rebound. We describe a few new hitherto not yet known ligands, identify false positives in the current literature, and report on ligands that we have seen as contaminations and that remain to be identified.

We measured the calcium response in the intact cells along the antennae of the flies. Therefore, the signal represents the responses of olfactory receptor proteins (say, Or92a) in situ and in vivo, including the effects of sensillar lymph, olfactory binding proteins, and any additional molecular mechanism in the receptor complex and along the transduction cascade, including, possibly, additional receptors coexpressed in the same cells (Leal 2013; Rihani, Ferveur, and Briand 2021; Task et al. 2022). Therefore, the complex time courses observed in Or42b and Or92a could reflect a complexity of these receptor proteins (e.g., different binding sites) or could reflect a complexity in the receptor cells (e.g., additional receptors and/or transduction cascades), a question that remains to be investigated. It should be noted that we measured calcium concentration in receptor cells in the periphery, that is, calcium within the sensory dendrites and within the cell bodies, but not in the axons. We assume that the calcium response is highly correlated with the action potential frequency response that is relayed to the antennal lobe and thus to the fly's brain, but it may be that some modulation occurs during the transformation of the cellular response in the periphery and the action potential train along the axon. We also cannot exclude that the time course of calcium influx may differ between the sensory dendrites and the somata, two compartments that we cannot separate at our resolution.

### 4.1 Limitations of the Current Study

No odorant ligand screening is complete, given the vast universe of possible ligands. Here, we focused on mostly aliphatic and aromatic compounds in the range of molecular weights between 70 and 200 g/mol, based on the already known ligands of the receptors investigated. The aim was to increase the local resolution, characterize temporal complexity, understand concentration dependency within that olfactory space, and—for the receptors with a broader response profile—explore the boundaries of the response patterns. A necessary step for the future would be to expand the ligand library to as yet largely uncharted areas, such as sulfurous compounds or amines. Alternative approaches include using the gas chromatograph to disentangle natural substances that are in the flies' environments, for example, fruits, insects, and plants, to find the ligands that were likely driving forces in the evolution of the fly's olfactory system: this approach has been very successful in finding strong ligands (Stensmyr et al. 2003) but may be less useful to model the binding pocket of the receptor. However, modeling the binding pocket of the receptor would ideally be based on a more precise control of stimulus concentration. Here, we used headspace injections into the gas chromatograph. As a result, fewer molecules reached the antennae for substances with a low vapor pressure as compared with those with a high vapor pressure. In previous studies, we have shown that the insect olfactory system compensates its sensitivity to the vapor pressure of ligands (Sachse, Rappert, and Galizia 1999; Sachse and Galizia 2003), probably because the natural situation of an insect is to smell substances that are released into the air from a solid/liquid substrate, with exactly that property: low volatility leads to fewer airborne molecules for equal concentration in the substrate. Thus, our approach to use liquid dilution for defining concentrations reflects a solid ecological situation but may be less satisfactory for a quantitative analysis of receptor‐ligand binding properties.

It should also be noted that the magnitude of an FID response depends to a large degree on the number of carbon atoms in the elute, and also on other factors, such as degree of saturation and number of oxygen atoms. This contributes to our reluctance in interpreting our “dilutions” or “FID peak sizes” as if they were “stimulus concentrations.”

Some ligands appeared as contaminations within the commercially available odorants and gave strong responses. These could not yet be chemically characterized and will be the topic of a future study. The presence of these trace substances (i.e., with no visible response in the chemical FID trace, but with a strong calcium response in the receptor neuron) suggests that the current best ligand is—from the point of view of the receptor—an intermediate ligand. These as yet uncharacterized contaminations were found among others in Or42b with an unknown highly effective ligand that contaminates ethyl propionate. Other contaminations could be identified: we assume that commercial 3‐octanol is consistently contaminated with 1‐octen‐3‐ol, the best ligand for Or13a, leading to a false positive response to 3‐octanol in the literature (Figure 8). We also found a “contaminant” in Or47a but could identify it based on the elution time as pentyl acetate (Figure 7).

### 4.2 Some Receptors Have Responses With Complex Time Courses, Others Do Not

We used a mathematical linear–nonlinear approach to estimate the response parameters of olfactory receptors (Kato et al. 2014). This is important because most insect olfactory receptors have phasic response properties. Furthermore, rather than reflecting odorant concentration, their responses may reflect molecular flux in the sensillum (Kaissling 2013). Therefore, a response is not a yes/no state (even when the stimulus is given as a square pulse), but rather a temporal course. In the case of the GC eluate, that is even more important, since the substances leaving the column form a stimulus with a gradual concentration increase and a gradual concentration decrease. This temporal property of the stimulus was taken into account when fitting the receptor response time course with our model. We estimated the magnitude of three parameters, reflecting—in a simplified wording—response upstroke time constant (*k*_*A*_), response strength (*k*_*AF*_), and response decay time constant (*k*_*F*_). These three parameters created a temporally simple response: increase (or decrease) and return to baseline. We added a second component, adding a parameter for delay (*D*_*AS*_) and its own strength and time constants (*k*_*AS*_ and *k*_*S*_) (see Figure 1). We found that these two functions gave a very good fit for (almost) all our responses: the second component (*k*_*AS*_ and *k*_*S*_) allowed us to model biphasic responses and, in some cases, was used to generate longer tails in the response, suggesting that calcium decay was generated by two independent mechanisms: one fast and immediate and one slower and longer‐lasting. Visual inspection revealed a few instances where our assumptions were not sufficient: responses to high concentrations of 3‐octanol in Or47a, for example, had a fast upstroke and an immediate initial decay, followed by a slower long‐lasting decay, that could not be fitted with our assumptions. Maybe, in this particular case, there is a third mechanism happening at the molecular level, for example, some kind of very fast partial initial adaptation.

Across the vast majority of our samples, however, a single function was sufficient to model the response time course perfectly (i.e., the “S” component was not necessary): Or10a, Or13a, Or22a, Or47a, and Or56a all had (almost) exclusively calcium increases with no temporal complexity, suggesting a single transduction mechanism. This suggests that negative responses may be caused by interaction with the same binding pocket as the positive responses, with the ligands probably acting as competitive antagonists. This would be consistent with the single binding pocket apparent in structural analyses (Del Mármol, Yedlin, and Ruta 2021; Zhao et al. 2024). However, Or42b and Or92a had many biphasic responses (Figure S3), and their responses could only be modeled by assuming two independent processes, “F” and the delayed “S,” in our model, suggesting that also at a molecular level, there is either an interaction with different binding sites on the olfactory receptor protein or an interaction within the cell with different transduction cascades from an additional receptor coexpressed in the same cell. Here, we only used purified samples, directly eluting from a gas chromatograph column. Future experiments, for example, with dedicated odorant mixtures, will be needed to investigate the existence of multiple binding sites on the receptor, or multiple receptors in the cell. Such biphasic responses, however, are likely to introduce temporal complexity in more natural situations, when stimuli are not pure but mixtures of various substances and themselves temporally complex due to turbulences (Szyszka et al. 2014; Gorur‐Shandilya et al. 2017).

Temporal complexity to stimulation with different odorants is known in *Drosophila* (Getahun et al. 2012) and also from those receptors that we find to be temporally simple here (Or10a, Or22a, Or47a, and Or56a) (Münch and Galizia 2017). How can that be explained? We propose that the complexity in that study may be a consequence of the stimuli that were not purified with a gas chromatograph. Therefore, even though there was a dominant substance in the “monomolecular” stimulus, there might have been unknown contaminations that might have introduced mixture competition for the binding pocket, introducing an overlap of different time constants in their response share, leading to complex temporal time courses. Therefore, the investigation of odor‐response properties to odorant mixtures remains an open field for future studies: even “pure monomolecular” stimuli turn out to be mixtures.

### 4.3 Responses to Chiral Substances

Chiral substances can have a different odor: a common example for humans is carvone, which smells as caraway or as spearmint depending on its chirality (Bentley 2006). We disentangled responses to chiral substances by using a polar column that has different elution times for chiral substances. Thus, we could confirm different responses for chiral substances, as already reported in other olfactory studies (Pelz et al. 2006). These included different responses to steric conformations of 2,3‐butanediol in Or92a (see Figure 3). 2,3‐Butanediol for Or92a is an interesting case: the receptor is highly responsive to 2,3‐butanedion (diacetyl), which does not have any steric variants. Thus, the selectivity for some chiral conformations to the lesser ligand 2,3‐butanediol may reflect their geometric 3D similarity to diacetyl.

### 4.4 Long‐Lasting Responses at High Concentrations

We found that some responses, in particular at high concentrations, were long lasting, with decay times of several seconds, up to minutes. This could mean that these receptors are effectively blinded by such stimuli. Given that in other situations, the olfactory system has the capacity to smell in the temporal realm of milliseconds (Stierle, Galizia, and Szyszka 2013), this finding is astounding. How would an olfactory system compensate for that? More research would be needed here. However, it is possible that in a natural situation, the fruit fly would rarely encounter these substances at such a high concentration, and the long‐lasting response remains an important laboratory observation (important to estimate detachment times between ligand and receptor), but an irrelevant phenomenon in the natural environment. Alternatively, it may be that the olfactory system cannot use temporal complexity to disentangle odor sources when they mix at high concentrations. The latter property is used, for example, in mating disruption campaigns for crop protection (Carde 1990).

### 4.5 Responses to Weak Ligands

Sometimes, receptors are labelled for their best ligand: “the 1‐octen‐3‐ol receptor” could be a good name for Or13a. Weak responses to other ligands might be irrelevant for the olfactory system at large. Assuming that these weak responses are close to background or stochastic activity and that other receptors might give strong responses to these odorants, the brain would not need the weak responses to identify the odor. However, the situation might differ in the case of odorant mixtures (which constitute most of the olfactory stimuli in nature): here, adding a weak ligand to a strong ligand may reduce its response due to competitive interaction at the binding site of the receptor. Such mixture effect cannot be studied with gas chromatograph purified stimulation but rather needs dedicated experimental designs.

### 4.6 Screening Efforts for Olfactory Receptors

We believe that this study, along with similar efforts including the *Drosophila* larva (Mathew et al. 2013; Si et al. 2019), is an important step for characterizing the basis of olfactory coding. How does an olfactory system encode odorants? And how are receptor response profiles related to the statistics of molecule concentrations found in a natural environment, which have been shown to follow a hyperbolic geometry (Zhou, Smith, and Sharpee 2018; Zhou et al. 2024; Ghaninia et al. 2022)? Understanding the combinatorial nature ideally necessitates understanding the odor‐response profiles of each and every receptor within an olfactory system. Note that also highly selective receptors rely on the combinatorial code: activity of Or56a reliably reports the presence of geosmin in the environment (highly selective receptor), but the combinatorial code in other receptors gives the olfactory system information about the context in which this substance is found (e.g., for geosmin, which is a mold signature, it is relevant for the fly to smell whether the source is in a patch of green grass, or in a patch of fresh fruit, or of slightly rotten fruit). Here, we report a (partial) screen on seven ORs out of approx. 60 ORs: there remains a lot of work to do. Many research groups contribute to the knowledge of response profiles, and efforts to collect such data have been established for different species (Skoufos et al. 2000; Nagarathnam et al. 2014; Münch and Galizia 2016; Sharma et al. 2022; Lalis et al. 2024). However, our result that only five out of our sample of seven ORs could be modeled with a simple temporal function, whereas the other two (Or42b and Or92a) necessitate at least two distinct temporal responses creates the necessity to amend these databases: the response of a receptor to a ligand cannot be reported as a single number (“response strength” or “binding strength”) anymore but needs more complex information. Similarly, databases need to consider concentration‐response relationships. Understanding how the antennal lobe and other brain networks downstream process the resulting complex temporal and combinatorial patterns will be a fascinating challenge.

## Author Contributions

**Alja Lüdke:** data curation, formal analysis, investigation, validation, visualization, writing – review and editing. **Ajayrama Kumaraswamy:** formal analysis, investigation, methodology, software, visualization, writing – review and editing. **C. Giovanni Galizia:** conceptualization, data curation, formal analysis, funding acquisition, investigation, methodology, project administration, resources, software, supervision, validation, visualization, writing – original draft, writing – review and editing.

## Ethics Statement

All experiments were done with fruit flies, *D. melanogaster* , bred specifically for the purpose of these experiments.

## Conflicts of Interest

The authors declare no conflicts of interest.

### Peer Review

The peer review history for this article is available at https://www.webofscience.com/api/gateway/wos/peer‐review/10.1111/ejn.70036.

## Supporting information


**Table S1** Odorants used in this study. The abbreviated letter code, chemical name, CAS, and InChiKey codes are provided. For some odorants, we introduced more than one entry, with different letter codes, e.g. when these odorants elicited more than one FID or calcium peak. Linalool oxide (LIOL) elicited two FID peaks on the nonpolar and polar column and these peaks were always listed as LIOL1 and LIOL2, although they came from the same commercially bought vial of Linalool oxide; they may be the chiral forms. MCHL showed one FID peak using the nonpolar column, but two peaks using the polar column. Thus, for the analysis of the polar column measurements we replaced the MCHL elution time with two elution times (MCHL1 and MCHL2). 2,3‐Butanediol (rac) (BDOL) is a racemic mixture of the three isomers: the two chiral isomers (2R,3R)‐(−)‐2,3‐Butanediol (RBDL), (2S,3S)‐(+)‐2,3‐Butanediol (SBDL) and the meso‐isomer meso‐2,3‐Butanediol (MBDL). All three isomers were also bought and measured as pure isomers separately (SBDL, RBDL, and MBDL). However, when measuring BDOL (rac), it gave two clearly separated FID peaks and calcium responses in the polar column. The earlier peak was at the elution time of the two chiral forms (SBDL and RBDL, eluting at the same time) and the later peak was at the elution time of MBDL. Since BDOL was applied to the polar column from one vial but gave separated peaks, we named these peaks according to their elution time BDOL_SR and BDOL_M. Using the nonpolar column, BDOL (rac) also gave two peaks, but these were strongly overlapping and could not be separated. Here we used the elution time of the earlier peak, corresponding to the SBDL and RBDL elution time, and just stuck to the letter code: BDOL. A second table in Appendix Table 1 lists all suppliers.

**Appendix Table 1** Suppliers.

**Table S2** Relates to Figure 4. Modelled response values for all Or lines, single file for each line.

**Appendix Table 2** Or13a.

**Appendix Table 2** Or22a.

**Appendix Table 2** Or42b.

**Appendix Table 2** Or47a.

**Appendix Table 2** Or56a.

**Appendix Table 2** Or92a.

**Fig. S1** The summary plot for Or92a shows the time traces of the GC‐FID and of the calcium recording to a mixture of odorants (here: MixC at a dilution of 10^−2^). In this experiment, the nonpolar column was used (for comparison: the same MixC, separated by the polar column is shown in Figure S2). Time (minutes) is shown from bottom to top, the FID trace is shown on the left side and the aligned calcium traces on the right side. Each trace is the calcium recording of a single fly (here *n* = 5, fly names top right). The odorants added to MixC are shown in the middle (BDOL, MBAE, EM2E, ZHAE, FENT, MSAM, GEST) and for all odorants an FID signal is visible. Note that two strong calcium responses, without a corresponding FID signal, or known odorant application were observed here and marked as BEDNXX and EM2EXX (these contaminations were also observed in the single odorant measurements of BEDN and EM2E, hence the code). BDOL elicited the overlapping FID peaks (the first peak comes from the chiral pair SBDL and RBDL, and the second peak from the meso‐isomer MBDL). Since the peaks could not be separated for analysis, we stuck to the name of BDOL for the first peak and neglected the second (unlike for the polar column, see Figure S2).

**Fig. S2** The summary plot for Or92a shows the time traces of the GC‐FID and of the calcium recording to MixC at a dilution of 10^−2^. In this experiment, the polar column was used (for comparison: MixC, separated by the nonpolar column, is shown in Figure S1). Time (minutes) is shown from bottom to top, the FID trace is shown on the left side and the aligned calcium traces on the right side. Each trace is the calcium recording of a single fly (here *n* = 4). The odorants added to MixC are shown in the middle (GEST, MBAE, EM2E, ZHAE, FENT, BDOL_SR, BDOL_M, MSAM). Note, that the order of the eluting odorants and the elution times are different from the nonpolar column (Figure S1). The two strong calcium responses to contaminations, named BEDNXX and EM2EXX, which appear without a corresponding FID signal or known odorant application in the mix, were also observed here and marked (these contaminations were also observed in the single odorant measurements of BEDN and EM2E). The racemic mixture BDOL elicited two clearly separated FID peaks on the polar column, corresponding to the elution times of SBDL/RBDL (first peak) and MBDL (second peak). Since the peaks could be separated for analysis, we labeled the two BDOL peaks as BDOL_SR and BDOL_M, respectively.

**Fig. S3** Responses to different chiral forms of 2,3‐butanediol in Or92a (left column), and corresponding linear‐non‐linear modeling (right column). All responses were biphasic: first positive, and then with an undershoot below baseline. Weaker responses (meso form) look graphically more noisy traces due to their scaling. The bottom trace is shorter because the following response to another substance must not be included in the modeling.

**Fig. S4** The summary plot of Or13a shows the only observed negative response in this Or line to the odorant 2‐methylphenol (2MPM). The negative response was dose dependent (not shown). Time (minutes) is shown from bottom to top, the FID trace is shown on the left side and the aligned calcium traces on the right side. Each trace is the calcium recording of a single fly (here *n* = 5). Interestingly, in four flies the response was clearly negative, while one fly gave a positive response. This behavior correlated with the weaker (and non‐significant) responses to 4MPM approx. one minute later. CXXX is a contamination with 1‐octen‐3‐ol, the best‐known ligand for Or13a.

**Fig. S5** The summary plot of Or22a shows the calcium response to propanoic acid (PROS) in MixE (dilution 10^−2^). PROS consistently elicited an off response, i.e. a positive calcium response after stimulus offset, without a previous inhibitory “on”‐response during odor stimulation. This was the only stimulus to show this behavior in our dataset.

**Fig. S6** The summary plot of Or42b reveals a consistently strong calcium response to a contamination, occurring before ethyl propionate (ET3E). While ET3E eluted at 4:51 min (from the nonpolar column), the contamination (named ET3EXX) eluted at 4:11 min. The response to the contamination was about half the response to ET3E, but totally invisible in the FID trace, indicating that at this location a very strong ligand had eluted. Future experiments will be needed to identify this substance.

## Data Availability

All code is stored on Git and available upon request. Data will be uploaded to a repository upon publication.
