# Supplementary material for: Olfactory Receptor Responses to Pure Odorants in Drosophila melanogaster
Source: Eur J Neurosci. 2025 Mar 10;61(5):e70036. doi: 10.1111/ejn.70036 (PMC11891828; doi:10.1111/ejn.70036)
Supplement: Supplementary file 1 — Table S1 Odorants used in this study. The abbreviated letter code, chemical name, CAS, and InChiKey codes are provided. For some odorants, we introduced more than one entry, with different letter codes, e.g. when these odorants elicited more than one FID or calcium peak. Linalool oxide (LIOL) elicited two FID peaks on the nonpolar and polar column and these peaks were always listed as LIOL1 and LIOL2, although they came from the same commercially bought vial of Linalool oxide; they may be the chiral forms. MCHL showed one FID peak using the nonpolar column, but two peaks using the polar column. Thus, for the analysis of the polar column measurements we replaced the MCHL elution time with two elution times (MCHL1 and MCHL2). 2,3‐Butanediol (rac) (BDOL) is a racemic mixture of the three isomers: the two chiral isomers (2R,3R)‐(−)‐2,3‐Butanediol (RBDL), (2S,3S)‐(+)‐2,3‐Butanediol (SBDL) and the meso‐isomer meso‐2,3‐Butanediol (MBDL). All three isomers were also bought and measured as pure isomers separately (SBDL, RBDL, and MBDL). However, when measuring BDOL (rac), it gave two clearly separated FID peaks and calcium responses in the polar column. The earlier peak was at the elution time of the two chiral forms (SBDL and RBDL, eluting at the same time) and the later peak was at the elution time of MBDL. Since BDOL was applied to the polar column from one vial but gave separated peaks, we named these peaks according to their elution time BDOL_SR and BDOL_M. Using the nonpolar column, BDOL (rac) also gave two peaks, but these were strongly overlapping and could not be separated. Here we used the elution time of the earlier peak, corresponding to the SBDL and RBDL elution time, and just stuck to the letter code: BDOL. A second table in Appendix Table 1 lists all suppliers. [file EJN-61-0-s015.pdf]

Appendix_Table1

odor	Name	CAS	InChiKey
**2ACM**	2-phenethyl acetate	103-45-7	MDHYEMXUFSJLGV-UHFFFAOYSA-N
**2EBM**	ethyl benzoate	93-89-0	MTZQAGJQAFMTAQ-UHFFFAOYSA-N
**2EPM**	2-ethylphenol	90-00-6	IXQGCWUGDFDQMF-UHFFFAOYSA-N
**2MNL**	(±)-geosmin	16423-19-1	JLPUXFOGCDVKGO-GRYCIOLGSA-N
**2MPM**	2-methylphenol	95-48-7	QWVGKYWNOKOFNN-UHFFFAOYSA-N
**2PPM**	2-propylphenol	644-35-9	LCHYEKKJCUJAKN-UHFFFAOYSA-N
**2RHL**	(R)-(-)-2-hexanol	26549-24-6	QNVRIHYSUZMSGM-ZCFIWIBFSA-N
**3CAT**	3-carene	13466-78-9	BQOFWKZOCNGFEC-UHFFFAOYSA-N
**3HXN**	3-hexanone	589-38-8	PFCHFHIRKBAQGU-UHFFFAOYSA-N
**3MPM**	3-methylphenol	108-39-4	RLSSMJSEOOYNOY-UHFFFAOYSA-N
**4MPM**	4-methylphenol	106-44-5	IWDCLRJOBJJRNH-UHFFFAOYSA-N
**ACEA**	acetaldehyde	75-07-0	IKHGUXGNUITLKF-UHFFFAOYSA-N
**ALOT**	α-ionone	127-41-3	UZFLPKAIBPNNCA-BQYQJAHWSA-N
**APNT**	α-pinene	80-56-8	GRWFGVWFFZKLTI-UHFFFAOYSA-N
**BACE**	butyl acetate	123-86-4	DKPFZGUDAPQIHT-UHFFFAOYSA-N
**BBTL**	β-butyrolactone	3068-88-0	GSCLMSFRWBPUSK-UHFFFAOYSA-N
**BDOL**	2,3-butanediol (rac)	513-85-9	OWBTYPJTUOEWEK-UHFFFAOYSA-N
**BDOL_M**	2,3-butanediol (meso)	513-85-9	OWBTYPJTUOEWEK-UHFFFAOYSA-N
**BDOL_SR**	2,3-butanediol (rac of S and R)	513-85-9	OWBTYPJTUOEWEK-UHFFFAOYSA-N
**BEAM**	benzaldehyde	100-52-7	HUMNYLRZRPPJDN-UHFFFAOYSA-N
**BEDN**	2,3-butanedione	431-03-8	QSJXEFYPDANLFS-UHFFFAOYSA-N
**BIST**	α-bisabolol	23089-26-1	RGZSQWQPBWRIAQ-CABCVRRESA-N
**BJOT**	β-ionone	79-77-6	PSQYTAPXSHCGMF-BQYQJAHWSA-N
**BMYT**	myrcene	123-35-3	UAHWPYUMFXYFJY-UHFFFAOYSA-N
**BNIM**	benzonitrile	100-47-0	JFDZBHWFFUWGJE-UHFFFAOYSA-N
**BOLM**	benzyl alcohol	100-51-6	WVDDGKGOMKODPV-UHFFFAOYSA-N
**BU3E**	butyl propionate	590-01-2	BTMVHUNTONAYDX-UHFFFAOYSA-N
**BUBE**	butyl butanoate	109-21-7	XUPYJHCZDLZNFP-UHFFFAOYSA-N
**BUTL**	1-butanol	71-36-3	LRHPLDYGYMQRHN-UHFFFAOYSA-N
**BUTN**	2-butanone	78-93-3	ZWEHNKRNPOVVGH-UHFFFAOYSA-N
**CAPT**	β-caryophyllene	87-44-5	NPNUFJAVOOONJE-GFUGXAQUSA-N
**CART**	(R)-(-)-carvone	6485-40-1	ULDHMXUKGWMISQ-SECBINFHSA-N
**CAST**	(S)-(+)-carvone	2244-16-8	ULDHMXUKGWMISQ-VIFPVBQESA-N
**CILT**	β-citronellol	106-22-9	QMVPMAAFGQKVCJ-UHFFFAOYSA-N

**CINT**	1,8-cineole	470-82-6	WEEGYLXZBRQIMU-UHFFFAOYSA-N
**CITT**	citral	5392-40-5	WTEVQBCEXWBHNA-JXMROGBWSA-N
**CXXX**	1-octen-3-ol contamination	3391-86-4	VSMOENVRRABVKN-UHFFFAOYSA-N
**CYHL**	cyclohexanol	108-93-0	HPXRVTGHNJAIIH-UHFFFAOYSA-N
**DAPK**	cadaverine	462-94-2	VHRGRCVQAFMJIZ-UHFFFAOYSA-N
**DDEL**	δ-decalactone	705-86-2	GHBSPIPJMLAMEP-UHFFFAOYSA-N
**DECA**	decanal	112-31-2	KSMVZQYAVGTKIV-UHFFFAOYSA-N
**DECL**	1-decanol	112-30-1	MWKFXSUHUHTGQN-UHFFFAOYSA-N
**DESE**	diethyl succinate	123-25-1	DKMROQRQHGEIOW-UHFFFAOYSA-N
**DMBM**	4-allyl-1,2-dimethoxybenzene	93-15-2	ZYEMGPIYFIJGTP-UHFFFAOYSA-N
**DMSK**	dimethyl sulfide	75-18-3	QMMFVYPAHWMCMS-UHFFFAOYSA-N
**E2BE**	ethyl trans-2-butenoate	623-70-1	ZFDIRQKJPRINOQ-HWKANZROSA-N
**E3HE**	ethyl 3-hydroxyhexanoate	2305-25-1	LYRIITRHDCNUHV-UHFFFAOYSA-N
**EACE**	ethyl acetate	141-78-6	XEKOWRVHYACXOJ-UHFFFAOYSA-N
**EHAE**	E2-hexenyl acetate	2497-18-9	HRHOWZHRCRZVCU-AATRIKPKSA-N
**EHBE**	ethyl 3-hydroxybutanoate (rac)	5405-41-4	OMSUIQOIVADKIM-UHFFFAOYSA-N
**EM2E**	ethyl tiglate	5837-78-5	OAPHLAAOJMTMLY-GQCTYLIASA-N
**EMBE**	ethyl 2-methylbutanoate	7452-79-1	HCRBXQFHJMCTLF-UHFFFAOYSA-N
**EMSE**	ethyl 3-methylsulfanylpropanoate	13327-56-5	YSNWHRKJEKWJNY-UHFFFAOYSA-N
**EMTE**	ethyl methanoate	109-94-4	WBJINCZRORDGAQ-UHFFFAOYSA-N
**EOPE**	ethyl 4-oxoperitanoate	539-88-8	GMEONFUTDYJSNV-UHFFFAOYSA-N
**ERHE**	ethyl (R)-(-)-3-hydroxybutanoate	24915-95-5	OMSUIQOIVADKIM-RXMQYKEDSA-N
**ESHE**	ethyl (S)-(+)-3-hydroxybutyrate	56816-01-4	OMSUIQOIVADKIM-YFKPBYRVSA-N
**ET3E**	ethyl propionate	105-37-3	FKRCODPIKNYEAC-UHFFFAOYSA-N
**ETAS**	ethanoic acid	64-19-7	QTBSBXVTEAMEQO-UHFFFAOYSA-N
**ETBE**	ethyl butyrate	105-54-4	OBNCKNCVKJNDBV-UHFFFAOYSA-N
**ETDE**	ethyl decanoate	110-38-3	RGXWDWUGBIJHDO-UHFFFAOYSA-N
**ETHE**	ethyl hexanoate	123-66-0	SHZIWNPUGXLXDT-UHFFFAOYSA-N
**ETHS**	2-ethylhexanoic acid	149-57-5	OBETXYAYXDNJHR-UHFFFAOYSA-N
**ETOE**	ethyloctanoate	106-32-1	YYZUSRORWSJGET-UHFFFAOYSA-N
**EUGM**	eugenol	97-53-0	RRAFCDWBNXTKKO-UHFFFAOYSA-N
**FENT**	(1R)-(-)-fenchone	7787-20-4	LHXDLQBQYFFVNW-OIBJUYFYSA-N
**FURL**	furfural	98-01-1	HYBBIBNJHNGZAN-UHFFFAOYSA-N
**GDEL**	γ-decalactone	706-14-9	IFYYFLINQYPWGJ-UHFFFAOYSA-N
**GEST**	geranyl acetate	105-87-3	HIGQPQRQIQDZMP-DHZHZOJOSA-N
**GHXL**	γ-hexalactone	695-06-7	JBFHTYHTHYHCDJ-UHFFFAOYSA-N

**GVAL**	γ-valerolactone	108-29-2	GAEKPEKOJKCEMS-UHFFFAOYSA-N
**H21L**	trans-2-hexen-1-ol	928-95-0	ZCHHRLHTBGRGOT-SNAWJCMRSA-N
**H31L**	trans-3-hexen-1-ol	928-97-2	UFLHIIWVXFIJGU-ONEGZZNKSA-N
**H3XL**	3-hexanol	623-37-0	ZOCHHNOQQHDWHG-UHFFFAOYSA-N
**HEPA**	heptanal	111-71-7	FXHGMKSSBGDXIY-UHFFFAOYSA-N
**HEPK**	heptane	142-82-5	IMNFDUFMRHMDMM-UHFFFAOYSA-N
**HEPN**	2-heptanone	110-43-0	CATSNJVOTSVZJV-UHFFFAOYSA-N
**HEXA**	hexanal	66-25-1	JARKCYVAAOWBJS-UHFFFAOYSA-N
**HEXL**	1-hexanol	111-27-3	ZSIAUFGUXNUGDI-UHFFFAOYSA-N
**HEXN**	2-hexanone	591-78-6	QQZOPKMRPOGIEB-UHFFFAOYSA-N
**HEXS**	hexanoic acid	142-62-1	FUZZWVXGSFPDMH-UHFFFAOYSA-N
**HP2L**	2-heptanol	543-49-7	CETWDUZRCINIHU-UHFFFAOYSA-N
**HPAE**	heptyl acetate	112-06-1	ZCZSIDMEHXZRLG-UHFFFAOYSA-N
**HX2A**	trans-2-hexenal	6728-26-3	MBDOYVRWFFCFHM-SNAWJCMRSA-N
**HX2L**	(±)-2-hexanol (rac)	626-93-7	QNVRIHYSUZMSGM-UHFFFAOYSA-N
**HX3L**	1-hexen-3-ol	4798-44-1	BVOSSZSHBZQJOI-UHFFFAOYSA-N
**HXAE**	hexyl acetate	142-92-7	AOGQPLXWSUTHQB-UHFFFAOYSA-N
**HXBE**	hexyl butanoate	2639-63-6	XAPCMTMQBXLDBB-UHFFFAOYSA-N
**HXHE**	hexyl hexanoate	6378-65-0	NCDCLPBOMHPFCV-UHFFFAOYSA-N
**IATE**	isoamyl tiglate	41519-18-0	ZARFDQHJMNVNLE-WEVVVXLNSA-N
**IBAE**	isobutyl acetate	110-19-0	GJRQTCIYDGXPES-UHFFFAOYSA-N
**IPBM**	4-isopropylbenzaldehyde	122-03-2	WTWBUQJHJGUZCY-UHFFFAOYSA-N
**IPES**	isopentanoic acid	503-74-2	GWYFCOCPABKNJV-UHFFFAOYSA-N
**ISOE**	isoamyl acetate	123-92-2	MLFHJEHSLIIPHL-UHFFFAOYSA-N
**LIMT**	(R)-(+)-limonene	5989-27-5	XMGQYMWWDOXHJM-JTQLQIEISA-N
**LINT**	linalool	78-70-6	CDOSHBSSFJOMGT-UHFFFAOYSA-N
**LIOL1**	linalool oxide (peak1)	60047-17-8	BRHDDEIRQPDPMG-UHFFFAOYSA-N
**LIOL2**	linalool oxide (peak2)	60047-17-8	BRHDDEIRQPDPMG-UHFFFAOYSA-N
**M3HE**	methyl 3-hydroxyhexanoate	21188-58-9	ACCRBMDJCPPJDX-UHFFFAOYSA-N
**MBAE**	2-methylbutyl acetate	624-41-9	XHIUFYZDQBSEMF-UHFFFAOYSA-N
**MBAM**	4-methoxybenzaldehyde	123-11-5	ZRSNZINYAWTAHE-UHFFFAOYSA-N
**MBDL**	meso-(2S,3R)-butanediol	5341-95-7	OWBTYPJTUOEWEK-ZXZARUISSA-N
**MBEL**	3-methyl-2-buten-1-ol	556-82-1	ASUAYTHWZCLXAN-UHFFFAOYSA-N
**MBZM**	methyl benzoate	93-58-3	QPJVMBTYPHYUOC-UHFFFAOYSA-N
**MCHL**	4-methylcyclohexanol (rac)	589-91-3	MQWCXKGKQLNYQG-UHFFFAOYSA-N
**MCHL1**	4-methylcyclohexanol (peak1)	589-91-3	MQWCXKGKQLNYQG-UHFFFAOYSA-N

**MCHL2**	4-methylcyclohexanol (peak2)	589-91-3	MQWCXKGKQLNYQG-UHFFFAOYSA-N
**MEBE**	methyl butyrate	623-42-7	UUIQMZJEGPQKFD-UHFFFAOYSA-N
**MEBM**	methoxybenzene	100-66-3	RDOXTESZEPMUJZ-UHFFFAOYSA-N
**MEHE**	methyl hexanoate	106-70-7	NUKZAGXMHTUAFE-UHFFFAOYSA-N
**MEOE**	methyl octanoate	111-11-5	JGHZJRVDZXSNKQ-UHFFFAOYSA-N
**MJSM**	methyljasmonate	39924-52-2	GEWDNTWNSAZUDX-SNAWJCMRSA-N
**MOL**	mineral oil	8042-47-5	
**MOL2**	mineral oil	8042-47-5	
**MPYM**	2,3-dimethylpyrazine	5910-89-4	OXQOBQJCDNLAPO-UHFFFAOYSA-N
**MSAM**	methylsalicylate	119-36-8	OSWPMRLSEDHDFF-UHFFFAOYSA-N
**MTIE**	methyl tiglate	6622-76-0	YYJWBYNQJLBIGS-SNAWJCMRSA-N
**MTPL**	3-(methylthio)-1-propanol	505-10-2	CZUGFKJYCPYHHV-UHFFFAOYSA-N
**NERL**	nerol	106-25-2	GLZPCOQZEFWAFX-YFHOEESVSA-N
**NONK**	n-nonane	111-84-2	BKIMMITUMNQMOS-UHFFFAOYSA-N
**NONN**	2-nonanone	821-55-6	VKCYHJWLYTUGCC-UHFFFAOYSA-N
**O13L**	1-octen-3-ol	3391-86-4	VSMOENVRRABVKN-UHFFFAOYSA-N
**OC3L**	3-octanol	589-98-0	NMRPBPVERJPACX-UHFFFAOYSA-N
**OC3N**	3-octanone	106-68-3	RHLVCLIPMVJYKS-UHFFFAOYSA-N
**OCAE**	octyl acetate	112-14-1	YLYBTZIQSIBWLI-UHFFFAOYSA-N
**OCTA**	octanal	124-13-0	NUJGJRNETVAIRJ-UHFFFAOYSA-N
**OCTK**	n-octane	111-65-9	TVMXDCGIABBOFY-UHFFFAOYSA-N
**OCTN**	2-octanone	111-13-7	ZPVFWPFBNIEHGJ-UHFFFAOYSA-N
**P2ON**	3-penten-2-one	625-33-2	LABTWGUMFABVFG-ONEGZZNKSA-N
**PACE**	pentyl acetate	628-63-7	PGMYKACGEOXYJE-UHFFFAOYSA-N
**PANM**	trans-p-propenylanisol	4180-23-8	RUVINXPYWBROJD-ONEGZZNKSA-N
**PARA**	paraldehyd	123-63-7	SQYNKIJPMDEDEG-UHFFFAOYSA-N
**PCYM**	p-cymene	99-87-6	HFPZCAJZSCWRBC-UHFFFAOYSA-N
**PE3L**	1-penten-3-ol	616-25-1	VHVMXWZXFBOANQ-UHFFFAOYSA-N
**PELM**	2-phenylethanol	60-12-8	WRMNZCZEMHIOCP-UHFFFAOYSA-N
**PENA**	pentanal	110-62-3	HGBOYTHUEUWSSQ-UHFFFAOYSA-N
**PENL**	1-pentanol	71-41-0	AMQJEAYHLZJPGS-UHFFFAOYSA-N
**PENM**	1-phenylethanone	98-86-2	KWOLFJPFCHCOCG-UHFFFAOYSA-N
**PENN**	2-pentanone	107-87-9	XNLICIUVMPYHGG-UHFFFAOYSA-N
**PENS**	pentanoic acid	109-52-4	NQPDZGIKBAWPEJ-UHFFFAOYSA-N
**PINT**	(+)-α-pinene	7785-70-8	GRWFGVWFFZKLTI-RKDXNWHRSA-N
**PR2A**	2-propenal	107-02-8	HGINCPLSRVDWNT-UHFFFAOYSA-N

**PRAE**	propyl acetate	109-60-4	YKYONYBAUNKHLG-UHFFFAOYSA-N
**PRBL**	γ-propyl-γ-butyrolactone	105-21-5	VLSVVMPLPMNWBH-UHFFFAOYSA-N
**PROA**	propanal	123-38-6	NBBJYMSMWIIQGU-UHFFFAOYSA-N
**PROS**	propanoic acid	79-09-4	XBDQKXXYIPTUBI-UHFFFAOYSA-N
**PXX3**	pentyl acetate contamination	628-63-7	PGMYKACGEOXYJE-UHFFFAOYSA-N
**PYRS**	pyruvic acid	127-17-3	LCTONWCANYUPML-UHFFFAOYSA-N
**RBDL**	(2R,3R)-(-)-2,3-butanediol	24347-58-8	OWBTYPJTUOEWEK-QWWZWVQMSA-N
**SBDL**	(2S,3S)-(+)-2,3-butanediol	19132-06-0	OWBTYPJTUOEWEK-IMJSIDKUSA-N
**TERT**	a-terpineole	10482-56-1	WUOACPNHFRMFPN-SECBINFHSA-N
**THUT**	(–)-α-thujone	546-80-5	USMNOWBWPHYOEA-MRTMQBJTSA-N
**Z2HL**	Z2-hexanol	928-94-9	ZCHHRLHTBGRGOT-PLNGDYQASA-N
**Z3HL**	Z3-hexen-1-ol	928-96-1	UFLHIIWVXFIJGU-ARJAWSKDSA-N
**ZHAE**	Z3-hexenyl acetate	3681-71-8	NPFVOOAXDOBMCE-PLNGDYQASA-N
