# Supplementary material for: Olfactory Receptor Responses to Pure Odorants in Drosophila melanogaster
Source: Eur J Neurosci. 2025 Mar 10;61(5):e70036. doi: 10.1111/ejn.70036 (PMC11891828; doi:10.1111/ejn.70036)
Supplement: Supplementary file 2 — Appendix Table 1 Suppliers. [file EJN-61-0-s001.pdf]

Appendix_Table1_suppliers

class	code	chem. name	CAS	synonyms	product no.	purity	company	InChI	InChIKey
**arom**	2ACM	phenethyl acetate	103-45-7	2-phenethyl acetate, 2-phenylethyl acetate	73747-5ML	≥97.0% (GC)	Sigma Aldrich	InChI=1S/C10H12O2/c1-9(11)12-8-7-10-5-3-2-4-6-10/h2-6H,7-8H2,1H3	MDHYEMXUFSJLGV-UHFFFAOYSA-N
**arom**	2EBM	ethyl benzoate	93-89-0		E12907-5G	> 99 %	Aldrich	InChI=1S/C9H10O2/c1-2-11-9(10)8-6-4-3-5-7-8/h3-7H,2H2,1H3	MTZQAGJQAFMTAQ-UHFFFAOYSA-N
**arom**	2EPM	2-ethyl phenol	90-00-6	phlorol	E44000-5G	99 %	Aldrich	InChI=1S/C8H10O/c1-2-7-5-3-4-6-8(7)9/h3-6,9H,2H2,1H3	IXQGCWUGDFDQMF-UHFFFAOYSA-N
**alcohol**	2MNL	(±)-geosmin	16423-19-1	2β,6α-dimethyl bicyclo[4.4.0]decan-1β-ol	UC18-5MG	> 97 %	Sigma		
**arom**	2MPM	2-methylphenol	95-48-7	o-cresol	C85700-5G	> 99 %	Aldrich	InChI=1S/C7H8O/c1-6-4-2-3-5-7(6)8/h2-5,8H,1H3	QWVGKYWNOKOFNN-UHFFFAOYSA-N
**arom**	2PPM	2-propyl phenol	644-35-9	o-propylphenol	P53608-25G	98 %	Aldrich	InChI=1S/C9H12O/c1-2-5-8-6-3-4-7-9(8)10/h3-4,6-7,10H,2,5H2,1H3	LCHYEKKJCUJAKN-UHFFFAOYSA-N
**alcohol**	2RHL	(R)-(-)-2-hexanol	26549-24-6		sc-253370		Santa Cruz Biotechnologies		
**terpene**	3CAT	3-carene	13466-78-9	δ3-carene, 3,7,7-trimethyl bicyclo[4.1.0]hept-3-ene	94415-1ML	≥97.0%	Sigma		BQOFWKZOCNGFEC-UHFFFAOYSA-N
**ketone**	3HXN	3-hexanone	589-38-8	ethyl propyl ketone	103020-10G	98 %	Aldrich	InChI=1S/C6H12O/c1-3-5-6(7)4-2/h3-5H2,1-2H3	PFCHFHIRKBAQGU-UHFFFAOYSA-N
**arom**	3MPM	3-methylphenol	108-39-4	m-cresol	C85727-5G	99 %	Aldrich	InChI=1S/C7H8O/c1-6-3-2-4-7(8)5-6/h2-5,8H,1H3	RLSSMJSEOOYNOY-UHFFFAOYSA-N
**arom**	4MPM	4-methylphenol	106-44-5	p-cresol	42429-5G-F	99.70 %	Fluka	InChI=1S/C7H8O/c1-6-2-4-7(8)5-3-6/h2-5,8H,1H3	IWDCLRJOBJJRNH-UHFFFAOYSA-N
**aldehyde**	ACEA	acetaldehyde	75-07-0	ethanal	402788-5ML	≥99.5%	Sigma		IKHGUXGNUITLKF-UHFFFAOYSA-N
**terpene**	ALOT	α-ionone	127-41-3	4-(2,6,6-Trimethyl-2-cyclohexen-1-yl)-3-buten-2-one	W259403-SAMPLE-K	> 90 %	Aldrich		
**terpene**	APNT	α-pinene	80-56-8	(±)-2-pinene, 2,6,6-trimethylbicyclo[3.1.1]hept-2-ene, alpha-Pinene	147524-5ML	98 %	Sigma		GRWFGVWFFZKLTI-UHFFFAOYSA-N
**ester**	BACE	butyl acetate	123-86-4		73285-1ML	> 99,7 %	Fluka		
**O rings**	BBTL	β-butyrolactone	3068-88-0	β-methyl-β-propiolactone, methyl-2-oxetanone	219126-10G	98 %	Aldrich	InChI=1S/C4H6O2/c1-3-2-4(5)6-3/h3H,2H2,1H3	GSCLMSFRWBPUSK-UHFFFAOYSA-N
**alcohol**	BDOL	2,3-butanediol	513-85-9	2,3-butylene glycol	B84904-100G	98 %	Aldrich		
**arom**	BEAM	benzaldehyde	100-52-7	bitter almond	09143-5ML-F	99.50 %	Fluka	InChI=1S/C7H6O/c8-6-7-4-2-1-3-5-7/h1-6H	HUMNYLRZRPPJDN-UHFFFAOYSA-N
**ketone**	BEDN	2,3-butanedione	431-03-8	biacetyl	11038-1ML-F	>99 %	Sigma	InChI=1S/C4H6O2/c1-3(5)4(2)6/h1-2H3	QSJXEFYPDANLFS-UHFFFAOYSA-N
**terpene**	BIST	α-bisabolol	23089-26-1	levomenol, (–)-6-methyl-2-(4-methyl-3-cyclohexen-1-yl)-5-hepten-2-ol	14462-5ML	> 95 %	Aldrich	InChI=1S/C15H26O/c1-12(2)6-5-11-15(4,16)14-9-7-13(3)8-10-14/h6-7,14,16H,5,8-11H2,1-4H3/t14-,15+/m1/s1	RGZSQWQPBWRIAQ-CABCVRRESA-N
**terpene**	BJOT	β-ionone	79-77-6	4-(2,6,6-Trimethyl-1-cyclohexenyl)-3-buten-2-one	W259500-SAMPLE-K	> 97 %	Aldrich		
**terpene**	BMYT	myrcene	123-35-3	β-myrcene, 7-methyl-3-methylene-1,6-octadiene	64643-100MG-F	≥90.0%	Sigma		UAHWPYUMFXYFJY-UHFFFAOYSA-N
**arom**	BNIM	benzonitrile	100-47-0	phenyl cyanide	270318-100ML	99.90 %	Aldrich	InChI=1S/C7H5N/c8-6-7-4-2-1-3-5-7/h1-5H	JFDZBHWFFUWGJE-UHFFFAOYSA-N
**arom**	BOLM	benzyl alcohol	100-51-6	benzenemethanol	08421-5ML-F	99.80 %	Fluka	InChI=1S/C7H8O/c8-6-7-4-2-1-3-5-7/h1-5,8H,6H2	WVDDGKGOMKODPV-UHFFFAOYSA-N
**ester**	BU3E	butyl propionate	590-01-2	butyl propanoate	W221104-SAMPLE-K	x	Aldrich		
**ester**	BUBE	butyl butanoate	109-21-7	butyl butyrate	67367-1ML	99.70 %	Fluka		
**aldehyde**	BUTA	butanal	123-72-8	butyraldehyde	418102-100ML	>99,5 %	Aldrich	InChI=1S/C4H8O/c1-2-3-4-5/h4H,2-3H2,1H3	ZTQSAGDEMFDKMZ-UHFFFAOYSA-N
**alcohol**	BUTL	1-butanol	71-36-3	butyl alcohol, n-butanol	19422-5ml	99.90 %	Fluka		
**ketone**	BUTN	2-butanone	78-93-3	MEK, Methyl ethyl ketone	34861-100ML	99.70 %	Sigma	InChI=1S/C4H8O/c1-3-4(2)5/h3H2,1-2H3	ZWEHNKRNPOVVGH-UHFFFAOYSA-N
**terpene**	CAPT	β-caryophyllene	87-44-5	(–)-trans-Caryophyllene	22075-1ML-F	> 98,5 %	Sigma	InChI=1S/C15H24/c1-11-6-5-7-12(2)13-10-15(3,4)14(13)9-8-11/h6,13-14H,2,5,7-10H2,1,3-4H3/b11-6+/t13-,14-/m1/s1	NPNUFJAVOOONJE-GFUGXAQUSA-N
**terpene**	CART	(R)-(-)-carvone	6485-40-1		124931-5ML	98 %	Aldrich	InChI=1S/C10H14O/c1-7(2)9-5-4-8(3)10(11)6-9/h4,9H,1,5-6H2,2-3H3/t9-/m1/s1	ULDHMXUKGWMISQ-SECBINFHSA-N
**terpene**	CAST	(S)-(+)-carvone	2244-16-8	(S)-5-isopropenyl-2-methyl-2-cyclohexenone	22070-1ML	99 %	Aldrich	InChI=1S/C10H14O/c1-7(2)9-5-4-8(3)10(11)6-9/h4,9H,1,5-6H2,2-3H3/t9-/m0/s1	ULDHMXUKGWMISQ-VIFPVBQESA-N
**terpene**	CILT	β-citronellol	106-22-9	3,7-Dimethyl-6-octen-1-ol	C83201-5G	95 %	Aldrich	InChI=1S/C10H20O/c1-9(2)5-4-6-10(3)7-8-11/h5,10-11H,4,6-8H2,1-3H3	QMVPMAAFGQKVCJ-UHFFFAOYSA-N
**terpene**	CINT	1,8-cineole	470-82-6	Eucalyptol	29210-1ML	> 99,0 %	Fluka	InChI=1S/C10H18O/c1-9(2)8-4-6-10(3,11-9)7-5-8/h8H,4-7H2,1-3H3	WEEGYLXZBRQIMU-UHFFFAOYSA-N
**terpene**	CITT	citral	5392-40-5	3,7-Dimethyl-2,6-octadienal	W230308-SAMPLE-K	> 96 %	Aldrich	InChI=1S/C10H16O/c1-9(2)5-4-6-10(3)7-8-11/h5,7-8H,4,6H2,1-3H3/b10-7+	WTEVQBCEXWBHNA-JXMROGBWSA-N
**alcohol**	CYHL	cyclohexanol	108-93-0	stensmyr	neu: 44113-1ML, vorher: 105899-25ML	99 %	Aldrich		
**other**	DAPK	cadaverine	462-94-2	1,5-diaminopentane, 1,5-pentanediamine, pentamethylenediamine	52063-1ML	≥96.5%	Sigma		VHRGRCVQAFMJIZ-UHFFFAOYSA-N
**O rings**	DDEL	δ-decalactone	705-86-2	δ-decanolactone, (±)-δ-pentyl-δ-valerolactone, (±)-5-decanolide, (±)-6-pentyltetrahydro-2H-pyran-2-one, 5-hydroxydecanoic acid δ-lactone	W236101-SAMPLE-K	≥98%	Sigma		GHBSPIPJMLAMEP-UHFFFAOYSA-N
**aldehyde**	DECA	decanal	112-31-2	decyl aldehyde, caprinaldehyde	D7384-25G	98 %	Sigma	InChI=1S/C10H20O/c1-2-3-4-5-6-7-8-9-10-11/h10H,2-9H2,1H3	KSMVZQYAVGTKIV-UHFFFAOYSA-N
**alcohol**	DECL	1-decanol	112-30-1		W236500-SAMPLE-K	> 98 %	Aldrich		
**ester**	DESE	diethyl succinate	123-25-1		07429-5ML-F	≥99.5%	Sigma		DKMROQRQHGEIOW-UHFFFAOYSA-N
**arom**	DMBM	4-allyl-1,2-dimethoxybenzene	93-15-2	eugenol methyl ether, Methyl eugenol	284424-5G	99 %	Aldrich	InChI=1S/C11H14O2/c1-4-5-9-6-7-10(12-2)11(8-9)13-3/h4,6-8H,1,5H2,2-3H3	ZYEMGPIYFIJGTP-UHFFFAOYSA-N
**other**	DMSK	dimethyl sulfide	75-18-3	DMS, Methyl sulfide	471577-25ML	≥99%	Sigma		QMMFVYPAHWMCMS-UHFFFAOYSA-N
**ester**	E2BE	ethyl trans-2-butenoate	623-70-1	ethyl crotonate	W348630-SAMPLE-K	≥97%	Sigma		ZFDIRQKJPRINOQ-HWKANZROSA-N
**ester**	E3HE	ethyl 3-hydroxyhexanoate	2305-25-1	tiglic acid	W354503-SAMPLE-K	> 98 %	Aldrich		
**ester**	EACE	ethyl acetate	141-78-6		58958-5ML	> 99,9 %	Fluka		
**ester**	EHAE	trans-2-hexenyl acetate	2497-18-9		W256404-SAMPLE-K	>98 %	Aldrich		
**ester**	EHBE	ethyl 3-hydroxybutanoate (rac)	5405-41-4	Ethyl 3-hydroxybutyrate	W342807-SAMPLE-K	> 97 %	Aldrich		
**ester**	EM2E	ethyl tiglate	5837-78-5	Ethyl-2-methylcrotonat, ethyl 2-methyl-2E-butenoate	W246018-SAMPLE	>98 %	Aldrich		
**ester**	EMBE	ethyl 2-methylbutanoate	7452-79-1	Ethyl 2-methylbutyrate	306886-5ML	99 %	Aldrich		
**ester**	EMSE	ethyl 3-methylsulfanylpropanoate	13327-56-5	Ethyl 3-(methylthio)propionate	W334308-SAMPLE-K	≥99%	Sigma		
**ester**	EMTE	ethyl methanoate	109-94-4	Ethyl formate	88554-1ML-F	≥99.5%	Sigma		WBJINCZRORDGAQ-UHFFFAOYSA-N
**ester**	EOPE	ethyl 4-oxoperitanoate	539-88-8	Ethyl 4-oxopentanoate, Ethyl levulinate	W244220-SAMPLE-K	≥98%	Sigma		
**ester**	ERHE	ethyl (R)-(-)-3-hydroxybutanoate	24915-95-5	Ethyl (R)-(–)-3-hydroxybutyrate	347329-1G	98 %	Aldrich		
**ester**	ESHE	ethyl (S)-(+)-3-hydroxybutyrate	56816-01-4	Ethyl (S)-3-hydroxy butanoate	374709-1G	99 %	Aldrich		
**ester**	ET3E	ethyl propionate	105-37-3	Propionsäureethylester	96727-1ML	>99,7 %	Fluka		
**acid**	ETAS	ethanoic acid	64-19-7	Glacial acetic acid	71251-5ML-F	≥99.8%	Aldrich		QTBSBXVTEAMEQO-UHFFFAOYSA-N
**ester**	ETBE	ethyl butyrate	105-54-4	ethyl butanoate / Buttersäureethylester	75563-5ML	99.50 %	Aldrich		
**ester**	ETDE	ethyl decanoate	110-38-3	Capric acid ethyl ester, Ethyl caprate	00733-1ML	≥99.0%	Sigma		RGXWDWUGBIJHDO-UHFFFAOYSA-N
**ester**	ETHE	ethyl hexanoate	123-66-0	ethyl caproate	148962-5ML	>99%	Aldrich		
**acid**	ETHS	2-ethylhexanoic acid	149-57-5	2-Ethylcaproic acid	02529-1ML-F	≥99.5%	Sigma		OBETXYAYXDNJHR-UHFFFAOYSA-N
**ester**	ETOE	ethyl octanoate	106-32-1	ethyl caprylate,	112321-5G, neu Okt 2018 W244902	99 %	Aldrich		
**arom**	EUGM	eugenol	97-53-0	2-Methoxy-4-(2-propenyl)phenol	E51791-5G	99 %	Aldrich	InChI=1S/C10H12O2/c1-3-4-8-5-6-9(11)10(7-8)12-2/h3,5-7,11H,1,4H2,2H3	RRAFCDWBNXTKKO-UHFFFAOYSA-N
**terpene**	FART	E,E-farnesol	106-28-5	(E,E)-3,7,11-Trimethyl-2,6,10-dodecatrien-1-ol	277541-1G	96 %	Aldrich	InChI=1S/C15H26O/c1-13(2)7-5-8-14(3)9-6-10-15(4)11-12-16/h7,9,11,16H,5-6,8,10,12H2,1-4H3/b14-9+,15-11+	CRDAMVZIKSXKFV-YFVJMOTDSA-N
**terpene**	FENT	(1R)-(-)-fenchone	7787-20-4	(1R)-1,3,3-trimethylbicyclo[2.2.1]heptan-2-one	196436-50G	> 98 %	Aldrich	InChI=1S/C10H16O/c1-9(2)7-4-5-10(3,6-7)8(9)11/h7H,4-6H2,1-3H3/t7-,10+/m0/s1	LHXDLQBQYFFVNW-OIBJUYFYSA-N

**O rings**	FURL	furfural	98-01-1	2-furaldehyde	W248924-SAMPLE-K	98 %	Aldrich	InChI=1S/C5H4O2/c6-4-5-2-1-3-7-5/h1-4H	HYBBIBNJHNGZAN-UHFFFAOYSA-N
**O rings**	GDEL	γ-decalactone	706-14-9	γ-decanolactone, 4-hydroxydecanoic acid γ-lactone	93738-1ML	≥98.0%	Sigma		IFYYFLINQYPWGJ-UHFFFAOYSA-N
**terpene**	GEST	geranyl acetate	105-87-3	trans-3,7-Dimethyl-2,6-octadienyl acetate	173495-25G	98 %	Aldrich		
**O rings**	GHXL	γ-hexalactone	695-06-7	γ-Caprolactone, γ-Ethyl-γ-butyrolactone	68554-1ML	≥99.0%	Sigma		JBFHTYHTHYHCDJ-UHFFFAOYSA-N
**O rings**	GVAL	γ-valerolactone	108-29-2	γ-Methyl-γ-butyrolactone	V403-100G	99 %	Aldrich	InChI=1S/C5H8O2/c1-4-2-3-5(6)7-4/h4H,2-3H2,1H3	GAEKPEKOJKCEMS-UHFFFAOYSA-N
**alcohol**	H21L	trans-2-hexen-1-ol	928-95-0		132667-25G (früher: W256218-SAMPLE-	96 %	Aldrich		ZCHHRLHTBGRGOT-SNAWJCMRSA-N
**alcohol**	H3XL	3-hexanol	623-37-0	Ethyl propyl carbinol	04649-1 ml	> 97 %	Fluka		
**aldehyde**	HEPA	heptanal	111-71-7	Heptaldehyde,1-Heptanal, Enanthaldehyde	H2120-2ML	95 %	Aldrich	InChI=1S/C7H14O/c1-2-3-4-5-6-7-8/h7H,2-6H2,1H3	FXHGMKSSBGDXIY-UHFFFAOYSA-N
**other**	HEPK	heptane	142-82-5	2,4-dimethylpentane	51730-5ML	> 99,8 %	Fluka		
**ketone**	HEPN	2-heptanone	110-43-0	Methyl pentyl ketone	02476-1ML	>99,8 %	Sigma	InChI=1S/C7H14O/c1-3-4-5-6-7(2)8/h3-6H2,1-2H3	CATSNJVOTSVZJV-UHFFFAOYSA-N
**aldehyde**	HEXA	hexanal	66-25-1	Hexyl aldehyde, Caproaldehyde, Aldehyde C-6	115606-2ML	98 %	Aldrich	InChI=1S/C6H12O/c1-2-3-4-5-6-7/h6H,2-5H2,1H3	JARKCYVAAOWBJS-UHFFFAOYSA-N
**alcohol**	HEXL	1-hexanol	111-27-3	hexyl alcohol	73117-1ML-F	>99,9 %	Fluka		
**ketone**	HEXN	2-hexanone	591-78-6	Butyl methyl ketone	02473-5ML	>99,5 %	Fluka	InChI=1S/C6H12O/c1-3-4-5-6(2)7/h3-5H2,1-2H3	QQZOPKMRPOGIEB-UHFFFAOYSA-N
**acid**	HEXS	hexanoic acid	142-62-1	caproic acid	21529-5ML	> 99 %	Fluka		
**alcohol**	HP2L	2-heptanol	543-49-7	Methyl pentyl carbinol	H3003-25g-S	98 %	Aldrich		
**ester**	HPAE	heptyl acetate	112-06-1	Essigsäureheptylester	W254703-SAMPLE-K	>98 %	Aldrich		
**aldehyde**	HX2A	trans-2-hexenal	6728-26-3	trans-2-Hexenal	132659-5G	98 %	Aldrich	InChI=1S/C6H10O/c1-2-3-4-5-6-7/h4-6H,2-3H2,1H3/b5-4+	MBDOYVRWFFCFHM-SNAWJCMRSA-N
**alcohol**	HX2L	(±)-2-hexanol (rac)	626-93-7	Butyl methyl carbinol	W513601-SAMPLE	> 98 %	Aldrich		
**alcohol**	HX3L	1-hexen-3-ol	4798-44-1		W360805-SAMPLE-K	> 98 %	Aldrich		
**ester**	HXAE	hexyl acetate	142-92-7	Capryl acetate	25539-1ML	> 99,7 %	Fluka		
**ester**	HXBE	hexyl butanoate	2639-63-6	Hexyl butyrate	W256803-SAMPLE-K	> 98 %	Aldrich		
**ester**	HXHE	hexyl hexanoate	6378-65-0	Hexyl caproate	18282-1ML	≥98.0%	Sigma		NCDCLPBOMHPFCV-UHFFFAOYSA-N
**ester**	IATE	isoamyl tiglate	41519-18-0		W501018-SAMPL	> 97%	SAFC		
**ester**	IBAE	isobutyl acetate	110-19-0		94823-1ML-F	99.80 %	Fluka		
**arom**	IPBM	4-isopropyl benzaldehyde	122-03-2	cuminaldehyde	W234109-SAMPLE-K	> 98 %	Aldrich	InChI=1S/C10H12O/c1-8(2)10-5-3-9(7-11)4-6-10/h3-8H,1-2H3	WTWBUQJHJGUZCY-UHFFFAOYSA-N
**acid**	IPES	iso-pentanoic acid	503-74-2	iso-valeric acid, 3-Methylbutanoic acid	W310204-SAMPLE	> 99 %	Aldrich		
**ester**	ISOE	isoamyl acetate	123-92-2	3-methylbutyl acetate, Isopentyl acetate	79857-5ML	>99,7 %	Sigma-Aldrich		
**terpene**	LIMT	(R)-(+)-Limonene	5989-27-5	R-1-Methyl-4-(1-methylethenyl)-cyclohexen, Carven; Dipenten	62118-1ML	> 99 %	Fluka	InChI=1S/C10H16/c1-8(2)10-6-4-9(3)5-7-10/h4,10H,1,5-7H2,2-3H3/t10-/m0/s1	XMGQYMWWDOXHJM-JTQLQIEISA-N
**terpene**	LIMT	(R)-(+)-Limonene	5989-27-5	R-1-Methyl-4-(1-methylethenyl)-cyclohexen, Carven; Dipenten	818,407	≥ 94.0 %	Merck		
**terpene**	LINT	linalool	78-70-6	(±)-3,7-Dimethyl-1,6-octadien-3-ol, (±)-3,7-Dimethyl-3-hydroxy-1,6-octadiene	L2602-100G	97 %	Sigma		
**O rings**	LIOL	linalool oxide	60047-17-8	2-(5-Methyl-5-vinyltetrahydro-1-furyl)-2-propanol	62141-25ML	≥97.0%	Sigma		BRHDDEIRQPDPMG-UHFFFAOYSA-N
**ester**	M3HE	methyl 3-hydroxyhexanoate	21188-58-9		W350818-SAMPLE-K	> 97 %	Aldrich		
**ester**	MBAE	2-methylbutyl acetate	624-41-9	sec-amyl acetate	W364401-SAMPLE-K	99 %	Aldrich		
**arom**	MBAM	4-methoxybenzaldehyde	123-11-5	p-Anisaldehyde	97063-1ML-F	99.00 %	Fluka	InChI=1S/C8H8O2/c1-10-8-4-2-7(6-9)3-5-8/h2-6H,1H3	ZRSNZINYAWTAHE-UHFFFAOYSA-N
**alcohol**	MBDL	meso-2,3-butanediol	5341-95-7		361461-10G	99 %	Aldrich		
**alcohol**	MBEL	3-methyl-2-buten-1-ol	556-82-1	3,3-Dimethylallyl alcohol, Prenol	162353-5ML	99 %	Sigma		ASUAYTHWZCLXAN-UHFFFAOYSA-N
**arom**	MBZM	methyl benzoate	93-58-3		18344-1ML-F	≥99.5%	Sigma		QPJVMBTYPHYUOC-UHFFFAOYSA-N
**alcohol**	MCHL	4-methylcyclohexanol (rac)	589-91-3	hexahydro-p-cresol	153095-250ML	98 %	Aldrich		
**ester**	MEBE	methyl butyrate	623-42-7		19358-1ML	>99,5 %	Fluka		
**arom**	MEBM	4-methoxybenzene	100-66-3	anisole	96109-5ML-F	>99,9 %	Fluka	InChI=1S/C7H8O/c1-8-7-5-3-2-4-6-7/h2-6H,1H3	RDOXTESZEPMUJZ-UHFFFAOYSA-N
**ester**	MEHE	methyl hexanoate	106-70-7	methyl caproate	21599-1ML-F	≥ 98.0 %	Merck		
**ester**	MEOE	methyl octanoate	111-11-5	Caprylic acid methyl ester, Methyl caprylate, Octanoic acid methyl ester	21719-5ML-F	≥99.8%	Sigma		JGHZJRVDZXSNKQ-UHFFFAOYSA-N
**acid**	METS	methanoic acid	64-18-6	Formic acid	W248703-SAMPLE-K	≥95%	Sigma		BDAGIHXWWSANSR-UHFFFAOYSA-N
**arom**	MJSM	methyljasmonate	39924-52-2	3-Oxo-2-(2-pentenyl)cyclopentaneacetic acid, methyl este	W341002-SAMPLE-K	> 95 %	Aldrich	InChI=1S/C13H20O3/c1-3-4-5-6-11-10(7-8-12(11)14)9-13(15)16-2/h4-5,10-11H,3,6-9H2,1-2H3/b5-4+	GEWDNTWNSAZUDX-SNAWJCMRSA-N
**oil**	MOL	mineral oil	8042-47-5		124020010		Acros Organics		
**alcohol**	MOPL	3-methoxy-1-propanol	1589-49-7		38457-10ML-F	≥98%	Sigma		JDFDHBSESGTDAL-UHFFFAOYSA-N
**arom**	MPYM	2,3-dimethylpyrazine	5910-89-4		07187-1ML	≥98.5%	Sigma		
**arom**	MSAM	methyl salicylate	119-36-8	methyl-2-hydroxybenzoate, Wintergreen oil	76631-1ML-F	> 99%	Fluka	InChI=1S/C8H8O3/c1-11-8(10)6-4-2-3-5-7(6)9/h2-5,9H,1H3	OSWPMRLSEDHDFF-UHFFFAOYSA-N
**alcohol**	MTBL	4-(methylmercapto)-1-butanol	20582-85-8	4-(Methylthio)-1-Butanol	319694-1G	97 %	Sigma		
**ester**	MTIE	methyl tiglate	6622-76-0	Methyl-2-methylcrotonat	279433		St Cruz		
**alcohol**	MTPL	3-(methylthio)-1-propanol	505-10-2	Methionol	W341509-SAMPLE-K	≥98%	Sigma		CZUGFKJYCPYHHV-UHFFFAOYSA-N
**alcohol**	NERL	nerol	106-25-2	cis-3,7-Dimethyl-2,6-octadien-1-ol, Nerol	50949-1ML	≥97.0%	Sigma		GLZPCOQZEFWAFX-YFHOEESVSA-N
**other**	NONK	n-nonane	111-84-2		74250-50ML	> 99,8 %	Fluka		
**ketone**	NONN	2-nonanone	821-55-6	Heptyl methyl ketone	W278505	>99,5 %	Sigma	InChI=1S/C9H18O/c1-3-4-5-6-7-8-9(2)10/h3-8H2,1-2H3	VKCYHJWLYTUGCC-UHFFFAOYSA-N
**alcohol**	O13L	1-octen-3-ol	3391-86-4	Pentyl vinyl carbinol	W280518-SAMPLE-K	> 98 %	Aldrich		
**alcohol**	OC3L	3-octanol	589-98-0	Ethyl pentyl carbinol	218405-50G	99 %	Aldrich		
**ketone**	OC3N	3-octanone	106-68-3	octan-3-one	46214-5ML	≥ 98.0 %	Sigma	InChI=1S/C8H16O/c1-3-5-6-7-8(9)4-2/h3-7H2,1-2H3	RHLVCLIPMVJYKS-UHFFFAOYSA-N
**ester**	OCAE	octyl acetate	112-14-1	Acetic acid octyl ester	O5500-5G-A	> 99 %	Aldrich		
**aldehyde**	OCTA	octanal	124-13-0	Octyl aldehyde, Caprylic aldehyde	O5608-25ML	99 %	Aldrich	InChI=1S/C8H16O/c1-2-3-4-5-6-7-8-9/h8H,2-7H2,1H3	NUJGJRNETVAIRJ-UHFFFAOYSA-N
**other**	OCTK	n-octane	111-65-9		74820-5ML	>99,7 %	Fluka		
**ketone**	OCTN	2-octanone	111-13-7	Hexyl methyl ketone	04709-25G	>98%	Sigma	InChI=1S/C8H16O/c1-3-4-5-6-7-8(2)9/h3-7H2,1-2H3	ZPVFWPFBNIEHGJ-UHFFFAOYSA-N
**ketone**	P2ON	3-penten-2-one	625-33-2	methyl propenyl ketone	W341703 SAMPLE-K	>70 %	Aldrich	InChI=1S/C5H8O/c1-3-4-5(2)6/h3-4H,1-2H3/b4-3+	LABTWGUMFABVFG-ONEGZZNKSA-N
**ester**	PACE	pentyl acetate	628-63-7	amyl acetate	W504009-SAMPLE	99 %	Aldrich		
**arom**	PANM	trans-p-propenylanisol	4180-23-8	trans-Anethole	10368-1ML	99.50 %	Fluka	InChI=1S/C10H12O/c1-3-4-9-5-7-10(11-2)8-6-9/h3-8H,1-2H3/b4-3+	RUVINXPYWBROJD-ONEGZZNKSA-N
**arom**	PCYM	p-cymene	99-87-6	1-Isopropyl-4-methylbenzene, 4-Isopropyltoluene	30039-5ML	≥99.5%	Sigma		HFPZCAJZSCWRBC-UHFFFAOYSA-N
**alcohol**	PE3L	1-penten-3-ol	616-25-1	Ethyl vinyl carbinol	01984-250MG	≥98.0%	Sigma		VHVMXWZXFBOANQ-UHFFFAOYSA-N
**arom**	PELM	2-phenylethanol	60-12-8	benzyl carbinol, 2-Phenylethyl alcohol	W285811-SAMPLE-K	>99 %	Aldrich	InChI=1S/C8H10O/c9-7-6-8-4-2-1-3-5-8/h1-5,9H,6-7H2	WRMNZCZEMHIOCP-UHFFFAOYSA-N
**aldehyde**	PENA	pentanal	110-62-3	valeraldehyde	W309818-SAMPLE-K	>97 %	Aldrich	InChI=1S/C5H10O/c1-2-3-4-5-6/h5H,2-4H2,1H3	HGBOYTHUEUWSSQ-UHFFFAOYSA-N

**alcohol**	PENL	1-Pentanol	71-41-0	n-Amyl alcohol	77597-1ML-F	99.80 %	Fluka		
**arom**	PENM	1-phenylethanone	98-86-2	acetophenone, Methyl phenyl ketone	42163-1ML-F	> 99,5 %	Fluka	InChI=1S/C8H8O/c1-7(9)8-5-3-2-4-6-8/h2-6H,1H3	KWOLFJPFCHCOCG-UHFFFAOYSA-N
**ketone**	PENN	2-pentanone	107-87-9	Methyl propyl ketone	46211-5ML	analytic.Stand	Fluka	InChI=1S/C5H10O/c1-3-4-5(2)6/h3-4H2,1-2H3	XNLICIUVMPYHGG-UHFFFAOYSA-N
**acid**	PENS	pentanoic acid	109-52-4	valeric acid	75054-1ML	>99,8 %	Fluka		
**terpene**	PINT	(+)-α-pinene	7785-70-8	(1R,5R)-2,6,6-Trimethylbicyclo[3.1.1]hept-2-ene	268070-5G	> 99 %	Aldrich		
**aldehyde**	PR2A	2-propenal	107-02-8	Acrolein	89116-1ML	≥99.0%	Sigma		HGINCPLSRVDWNT-UHFFFAOYSA-N
**ester**	PRAE	propyl acetate	109-60-4	Essigsäure-n-propylester	40858-1ML	99.70 %	Fluka		
**ester**	PRBE	n-propyl-n-butanoate	105-66-8	propyl butyrate	W293407-SAMPLE-K	> 98 %	Aldrich		
**O rings**	PRBL	γ-propyl-γ-butyrolactone	105-21-5	Gamma-Heptalactone, 5-propyloxolan-2-one, 4-heptanolide, Dihydro-5-propyl-2(3H)-furanone	W253901-SAMPLE-K	>98 %	Aldrich	InChI=1S/C7H12O2/c1-2-3-6-4-5-7(8)9-6/h6H,2-5H2,1H3	VLSVVMPLPMNWBH-UHFFFAOYSA-N
**aldehyde**	PROA	propanal	123-38-6	propionaldehyde	64409-1ML	≥98.0%	Merck	InChI=1S/C3H6O/c1-2-3-4/h3H,2H2,1H3	NBBJYMSMWIIQGU-UHFFFAOYSA-N
**acid**	PROS	propanoic acid	79-09-4	propionic acid	94425-1ML-F	> 99,8 %	Fluka		
**acid**	PYRS	pyruvic acid	127-17-3	α-Ketopropionic acid, 2-Oxopropionic acid	W297003-SAMPLE-K	≥97%	Sigma		LCTONWCANYUPML-UHFFFAOYSA-N
**alcohol**	RBDL	(2R,3R)-(-)-2,3-butanediol	24347-58-8		237639-1G	97 %	Aldrich		
**alcohol**	SBDL	(2S,3S)-(+)-2,3-butanediol	19132-06-0		300349-1G	97 %	Aldrich		
**terpene**	TERT	a-terpineole	10482-56-1	(–)-α-Terpineol	W304506-SAMPLE-K	> 96 %	SAFC		
**terpene**	THUT	(–)-α-thujone	546-80-5	(1S,4R)-1-Isopropyl-4-methylbicyclo[3.1.0]hexan-3-one	89231-1ML	> 96 %	Aldrich		
**alcohol**	Z2HL	Z2-hexanol	928-94-9	cis-2-Hexen-1-ol	224707-5G	95 %	Sigma		ZCHHRLHTBGRGOT-PLNGDYQASA-N
**alcohol**	Z3HL	Z3-hexen-1-ol	928-96-1	leaf alcohol, cis-3-Hexen-1-ol	W256307-SAMPLE-K	>98 %	Aldrich		
**ester**	ZHAE	Z3-hexenyl acetate	3681-71-8	3-Hexen-1-ol, (Z)-Hex-3-enylacetat	W317101-SAMPLE-K	>98 %	Aldrich		
