# Supplementary material for: Olfactory Receptor Responses to Pure Odorants in Drosophila melanogaster
Source: Eur J Neurosci. 2025 Mar 10;61(5):e70036. doi: 10.1111/ejn.70036 (PMC11891828; doi:10.1111/ejn.70036)
Supplement: Supplementary file 3 — Table S2 Relates to Figure 4. Modelled response values for all Or lines, single file for each line. [file EJN-61-0-s012.pdf]

Appendix_Table2_Or10a

odor code	num values	category no.	Odorant	response -2	response -4	response -6
**2EBM**	13	2	ethyl benzoate	10.08 ± 1.61	2.45 ± 0.87	-0.00 ± 0.17
**MBZM**	5	2	methyl benzoate	8.36 ± 3.30	3.00 ± 0.20	0.04 ± 0.11
**MSAM**	9	3	methylsalicylate	7.89 ± 2.56	5.84 ± 1.01	1.03 ± 0.79
**BACE**	11	1	butyl acetate	5.43 ± 0.24	0.00 ± 0.27	0.04 ± 0.21
**IBAE**	14	1	isobutyl acetate	5.21 ± 2.03	0.19 ± 0.10	-0.00 ± 0.09
**PENM**	11,4	1	1-phenylethanone	3.79 ± 0.61	0.06 ± 0.20	-0.05 ± 0.05
**E2BE**	6	0	ethyl trans-2-butenoate	1.76 ± 0.27	-0.05 ± 0.04	0.04 ± 0.13
**ISOE**	10	1	isoamyl acetate	1.69 ± 0.37	-0.06 ± 0.07	0.18 ± 0.12
**EM2E**	9	1	ethyl tiglate	1.55 ± 0.33	0.16 ± 0.10	-0.12 ± 0.18
**BNIM**	14	1	benzonitrile	1.18 ± 0.43	0.00 ± 0.10	0.08 ± 0.15
**BEAM**	7	1	benzaldehyde	0.88 ± 0.43	-0.21 ± 0.16	0.03 ± 0.03
**MEBM**	14	1	methoxybenzene	0.80 ± 0.29	-0.06 ± 0.12	-0.09 ± 0.09
**H21L**	14	1	trans-2-hexen-1-ol	0.77 ± 0.35	0.06 ± 0.09	-0.00 ± 0.10
**EACE**	13	1	ethyl acetate	0.70 ± 0.37	0.13 ± 0.16	0.00 ± 0.11
**MBAE**	9	0	2-methylbutyl acetate	0.60 ± 0.13	0.22 ± 0.31	-0.20 ± 0.20
**HEXL**	13	0	1-hexanol	0.48 ± 0.31	0.12 ± 0.20	-0.11 ± 0.11
**OC3L**	5	0	3-octanol	0.44 ± 0.29	0.20 ± 0.18	0.14 ± 0.14
**CILT**	5	0	β-citronellol	0.43 ± 0.69	0.04 ± 0.75	0.63 ± 0.28
**EMBE**	5	0	ethyl 2-methylbutanoate	0.39 ± 0.11	-0.31 ± 0.31	0.06 ± 0.12
**HEXN**	12	1	2-hexanone	0.31 ± 0.09	0.07 ± 0.09	0.00 ± 0.14
**PENA**	13	0	pentanal	0.31 ± 0.11	-0.00 ± 0.16	-0.05 ± 0.17
**ZHAE**	9	0	Z3-hexenyl acetate	0.27 ± 0.14	0.18 ± 0.21	0.10 ± 0.23
**GEST**	9	0	geranyl acetate	0.24 ± 0.24	0.26 ± 0.22	0.00 ± 0.26
**2ACM**	6	0	2-phenethyl acetate	0.22 ± 0.05	0.23 ± 0.32	0.07 ± 0.10
**MCHL**	5	0	4-methylcyclohexanol (rac)	0.21 ± 0.38	0.22 ± 0.14	-0.15 ± 0.08
**EOPE**	6	0	ethyl 4-oxoperitanoate	0.21 ± 0.11	0.13 ± 0.14	-0.07 ± 0.04
**NONN**	10	0	2-nonanone	0.20 ± 0.16	0.01 ± 0.06	0.04 ± 0.11
**FENT**	9	0	(1R)-(-)-fenchone	0.20 ± 0.18	0.22 ± 0.14	0.19 ± 0.19
**OCAE**	14	0	octyl acetate	0.18 ± 0.14	-0.04 ± 0.20	0.13 ± 0.14
**HX2L**	10	0	(±)-2-hexanol (rac)	0.17 ± 0.14	0.08 ± 0.25	-0.00 ± 0.19
**EMSE**	14	0	ethyl 3-methylsulfanylpropanoate	0.16 ± 0.17	0.01 ± 0.06	-0.09 ± 0.06
**3HXN**	6	0	3-hexanone	0.15 ± 0.16	-0.03 ± 0.15	-0.02 ± 0.08
**Z3HL**	14	0	Z3-hexen-1-ol	0.15 ± 0.18	0.17 ± 0.25	0.01 ± 0.09
**CINT**	11	0	1,8-cineole	0.15 ± 0.09	0.00 ± 0.21	0.14 ± 0.23
**2EPM**	12	0	2-ethylphenol	0.15 ± 0.13	0.09 ± 0.11	0.09 ± 0.11
**MCHL2**	5	0	4-methylcyclohexanol (peak2)	0.14 ± 0.14	-0.02 ± 0.24	0.13 ± 0.19
**Z2HL**	5	0	Z2-hexanol	0.13 ± 0.12	-0.03 ± 0.08	0.04 ± 0.04
**BOLM**	12	0	benzyl alcohol	0.13 ± 0.07	0.06 ± 0.06	-0.02 ± 0.13
**DECL**	7	0	1-decanol	0.13 ± 0.11	-0.21 ± 0.13	0.03 ± 0.03
**CART**	11	0	(R)-(-)-carvone	0.12 ± 0.09	-0.09 ± 0.26	0.08 ± 0.17

**HXHE**	14	0	hexyl hexanoate	0.12 ± 0.07	-0.07 ± 0.08	-0.01 ± 0.12
**PROA**	11	0	propanal	0.12 ± 0.07	0.18 ± 0.13	0.20 ± 0.24
**BDOL**	5	0	2,3-butanediol (rac)	0.12 ± 0.24	0.04 ± 0.25	0.12 ± 0.12
**2MPM**	13	0	2-methylphenol	0.11 ± 0.16	0.05 ± 0.12	0.00 ± 0.14
**M3HE**	14	0	methyl 3-hydroxyhexanoate	0.11 ± 0.15	0.09 ± 0.16	0.11 ± 0.13
**HP2L**	12	0	2-heptanol	0.11 ± 0.11	0.10 ± 0.13	-0.10 ± 0.06
**APNT**	7	0	α-pinene	0.11 ± 0.11	0.12 ± 0.13	-0.09 ± 0.18
**HX3L**	7	0	1-hexen-3-ol	0.10 ± 0.32	0.15 ± 0.11	0.17 ± 0.11
**OCTA**	10	0	octanal	0.10 ± 0.06	-0.04 ± 0.21	-0.02 ± 0.36
**PENS**	12	0	pentanoic acid	0.10 ± 0.19	0.18 ± 0.07	-0.01 ± 0.11
**GDEL**	1	0	γ-decalactone	0.09 ± 0.00	0.16 ± 0.00	-0.08 ± 0.00
**DMBM**	10	0	4-allyl-1,2-dimethoxybenzene	0.09 ± 0.23	-0.05 ± 0.21	-0.14 ± 0.15
**MEHE**	6	0	methyl hexanoate	0.08 ± 0.13	-0.06 ± 0.07	-0.06 ± 0.10
**NERL**	7	0	nerol	0.08 ± 0.10	-0.06 ± 0.09	0.06 ± 0.05
**PINT**	10	0	(+)-α-pinene	0.08 ± 0.08	0.07 ± 0.26	0.16 ± 0.18
**MCHL1**	5	0	4-methylcyclohexanol (peak1)	0.07 ± 0.07	0.15 ± 0.18	-0.10 ± 0.11
**PELM**	9	0	2-phenylethanol	0.07 ± 0.14	0.08 ± 0.10	-0.00 ± 0.19
**ALOT**	7	0	α-ionone	0.07 ± 0.26	0.09 ± 0.04	0.00 ± 0.10
**PROS**	14	0	propanoic acid	0.07 ± 0.11	-0.00 ± 0.18	-0.00 ± 0.08
**O13L**	13	0	1-octen-3-ol	0.07 ± 0.15	-0.00 ± 0.09	-0.09 ± 0.27
**IPES**	7	0	isopentanoic acid	0.07 ± 0.08	0.05 ± 0.14	-0.05 ± 0.18
**HPAE**	12	0	heptyl acetate	0.07 ± 0.07	-0.08 ± 0.13	0.00 ± 0.11
**EHAE**	12	0	E2-hexenyl acetate	0.07 ± 0.17	-0.08 ± 0.14	-0.00 ± 0.08
**BBTL**	5	0	β-butyrolactone	0.07 ± 0.12	-0.11 ± 0.31	0.00 ± 0.00
**GVAL**	12	0	γ-valerolactone	0.07 ± 0.10	0.08 ± 0.06	-0.10 ± 0.16
**H3XL**	10	0	3-hexanol	0.07 ± 0.12	0.14 ± 0.16	0.07 ± 0.08
**PARA**	7	0	paraldehyd	0.06 ± 0.02	-0.06 ± 0.14	0.13 ± 0.11
**LIOL2**	6	0	linalool oxide (peak2)	0.05 ± 0.07	-0.02 ± 0.08	-0.05 ± 0.12
**3CAT**	14	0	3-carene	0.05 ± 0.08	-0.04 ± 0.15	-0.05 ± 0.13
**DESE**	6	0	diethyl succinate	0.04 ± 0.09	-0.00 ± 0.15	0.06 ± 0.04
**CAST**	12	0	(S)-(+)-carvone	0.04 ± 0.15	-0.04 ± 0.13	0.07 ± 0.16
**EUGM**	14	0	eugenol	0.04 ± 0.17	0.07 ± 0.14	-0.05 ± 0.17
**LIMT**	7	0	(R)-(+)-limonene	0.03 ± 0.18	-0.05 ± 0.11	0.07 ± 0.07
**E3HE**	14	0	ethyl 3-hydroxyhexanoate	0.03 ± 0.10	0.00 ± 0.10	0.04 ± 0.15
**TERT**	4	0	a-terpineole	0.03 ± 0.16	0.02 ± 0.07	0.08 ± 0.16
**ETAS**	7	0	ethanoic acid	0.03 ± 0.05	0.10 ± 0.10	-0.02 ± 0.02
**OCTK**	12	0	n-octane	0.01 ± 0.10	0.11 ± 0.07	0.12 ± 0.10
**PR2A**	6	0	2-propenal	0.01 ± 0.18	0.01 ± 0.03	-0.01 ± 0.20
**PE3L**	6	0	1-penten-3-ol	0.00 ± 0.07	0.03 ± 0.07	0.03 ± 0.09
**OCTN**	11	0	2-octanone	-0.00 ± 0.29	-0.03 ± 0.07	-0.12 ± 0.14
**ESHE**	5	0	ethyl (S)-(+)-3-hydroxybutyrate	0.00 ± 0.00	-0.22 ± 0.22	0.15 ± 0.15
**ETBE**	9	0	ethyl butyrate	-0.00 ± 0.21	0.04 ± 0.05	-0.00 ± 0.05

**BDOL_SR**	4	0	2,3-butanediol (rac of S and R)	0.00 ± 0.06	0.19 ± 0.07	0.13 ± 0.09
**DECA**	10	0	decanal	0.00 ± 0.15	-0.16 ± 0.23	-0.13 ± 0.17
**HEPK**	5	0	heptane	0.00 ± 0.24	0.30 ± 0.19	-0.15 ± 0.11
**ACEA**	7	0	acetaldehyde	-0.00 ± 0.08	0.13 ± 0.13	0.13 ± 0.05
**MTPL**	6	0	3-(methylthio)-1-propanol	-0.02 ± 0.06	-0.02 ± 0.15	0.08 ± 0.17
**PANM**	10	0	trans-p-propenylanisol	-0.02 ± 0.19	0.10 ± 0.16	0.17 ± 0.17
**HEXA**	4	0	hexanal	-0.02 ± 0.09	-0.17 ± 0.02	-0.10 ± 0.10
**LINT**	10	0	linalool	-0.04 ± 0.18	-0.00 ± 0.10	0.07 ± 0.28
**ETOE**	5	0	ethyloctanoate	-0.04 ± 0.85	-0.15 ± 0.06	0.05 ± 0.08
**THUT**	7	0	(–)-α-thujone	-0.04 ± 0.12	0.11 ± 0.05	0.13 ± 0.19
**BMYT**	6	0	myrcene	-0.06 ± 0.08	-0.09 ± 0.11	0.07 ± 0.10
**2PPM**	7	0	2-propylphenol	-0.06 ± 0.10	-0.07 ± 0.08	0.09 ± 0.16
**2RHL**	5	0	(R)-(-)-2-hexanol	-0.07 ± 0.04	0.07 ± 0.02	-0.01 ± 0.12
**LIOL1**	5	0	linalool oxide (peak1)	-0.08 ± 0.03	-0.00 ± 0.02	0.06 ± 0.07
**IPBM**	12	0	4-isopropylbenzaldehyde	-0.09 ± 0.06	-0.09 ± 0.12	-0.07 ± 0.09
**BDOL_M**	4	0	2,3-butanediol (meso)	-0.09 ± 0.18	-0.05 ± 0.14	0.09 ± 0.12
**ET3E**	10	0	ethyl propionate	-0.10 ± 0.37	0.00 ± 0.24	0.18 ± 0.12
**PCYM**	5	0	p-cymene	-0.10 ± 0.13	-0.07 ± 0.02	0.07 ± 0.03
**EMTE**	5	0	ethyl methanoate	-0.10 ± 0.02	-0.04 ± 0.03	0.06 ± 0.07
**HXAE**	10	0	hexyl acetate	-0.10 ± 0.17	0.02 ± 0.28	-0.12 ± 0.32
**NONK**	11	0	n-nonane	-0.10 ± 0.18	0.14 ± 0.35	0.08 ± 0.07
**HEPA**	7	0	heptanal	-0.13 ± 0.03	0.00 ± 0.07	-0.10 ± 0.10
**BEDN**	9	0	2,3-butanedione	-0.14 ± 0.17	0.03 ± 0.15	0.04 ± 0.12
**PACE**	4	0	pentyl acetate	-0.14 ± 0.04	0.05 ± 0.21	-0.04 ± 0.08
**HEPN**	10	0	2-heptanone	-0.15 ± 0.21	-0.28 ± 0.09	0.25 ± 0.29
**HXBE**	10	0	hexyl butanoate	-0.16 ± 0.17	0.10 ± 0.08	0.06 ± 0.20
**PRAE**	4	0	propyl acetate	-0.17 ± 0.03	-0.21 ± 0.11	-0.01 ± 0.13
**PRBL**	4	0	γ-propyl-γ-butyrolactone	-0.28 ± 0.14	-0.28 ± 0.69	-0.05 ± 0.24
**BUTN**	4	0	2-butanone	-0.31 ± 0.07	-0.04 ± 0.04	-0.00 ± 0.08
**4MPM**	5	0	4-methylphenol	-0.41 ± 0.02	0.28 ± 0.29	0.33 ± 0.07
