# Supplementary material for: Olfactory Receptor Responses to Pure Odorants in Drosophila melanogaster
Source: Eur J Neurosci. 2025 Mar 10;61(5):e70036. doi: 10.1111/ejn.70036 (PMC11891828; doi:10.1111/ejn.70036)
Supplement: Supplementary file 4 — Appendix Table 2 Or13a. [file EJN-61-0-s007.pdf]

Appendix_Table2_Or13a

odor code	num values	category no.	Odorant	response -2	response -4	response -6
**HP2L**	8	3	2-heptanol	34.36 ± 5.59	16.92 ± 2.28	3.00 ± 0.90
**HX2L**	7	2	(±)-2-hexanol (rac)	32.83 ± 6.61	9.50 ± 3.99	-0.32 ± 0.32
**O13L**	17	3	1-octen-3-ol	29.62 ± 7.53	26.45 ± 5.71	13.87 ± 4.01
**HX3L**	9	2	1-hexen-3-ol	28.71 ± 2.77	5.84 ± 2.64	-0.05 ± 0.34
**ESHE**	9	2	ethyl (S)-(+)-3-hydroxybutyrate	21.65 ± 4.43	4.98 ± 1.93	-0.51 ± 0.51
**HEXL**	7	2	1-hexanol	20.82 ± 4.54	6.92 ± 1.36	0.41 ± 0.15
**H3XL**	8	1	3-hexanol	18.34 ± 2.29	0.84 ± 0.59	-0.26 ± 0.25
**Z3HL**	8	2	Z3-hexen-1-ol	17.86 ± 6.80	2.68 ± 1.60	0.47 ± 0.26
**HXAE**	7	2	hexyl acetate	14.11 ± 3.78	1.34 ± 0.44	-0.30 ± 0.72
**ZHAE**	8	1	Z3-hexenyl acetate	14.11 ± 5.06	0.99 ± 1.17	-0.18 ± 0.32
**M3HE**	8	1	methyl 3-hydroxyhexanoate	12.19 ± 4.60	1.29 ± 0.24	-0.54 ± 0.59
**HEPN**	7	1	2-heptanone	11.68 ± 1.72	-0.14 ± 0.66	0.81 ± 0.18
**OC3L**	9	2	3-octanol	8.96 ± 3.87	7.53 ± 1.36	0.70 ± 0.42
**BACE**	7	1	butyl acetate	8.85 ± 1.96	0.37 ± 0.27	0.24 ± 0.42
**OCTN**	7	1	2-octanone	8.67 ± 2.67	0.70 ± 0.19	-0.11 ± 0.57
**E3HE**	8	1	ethyl 3-hydroxyhexanoate	8.28 ± 3.81	0.50 ± 0.88	0.30 ± 0.37
**HEPA**	9	1	heptanal	6.69 ± 2.51	0.44 ± 0.78	-0.30 ± 0.24
**NONN**	8	1	2-nonanone	5.69 ± 1.44	-0.14 ± 0.79	-0.26 ± 0.51
**OCTA**	8	1	octanal	4.22 ± 1.54	-0.13 ± 0.56	0.06 ± 0.50
**PENA**	7	1	pentanal	4.20 ± 1.12	0.00 ± 0.43	0.12 ± 0.21
**EHAE**	8	1	E2-hexenyl acetate	3.76 ± 3.57	0.35 ± 0.92	0.20 ± 0.59
**ISOE**	8	1	isoamyl acetate	2.75 ± 1.19	-0.06 ± 0.31	0.24 ± 0.46
**LINT**	7	1	linalool	2.50 ± 0.40	-0.00 ± 0.47	-0.41 ± 0.40
**GVAL**	8	1	γ-valerolactone	2.19 ± 0.49	0.41 ± 0.40	0.00 ± 0.00
**HEXN**	8	0	2-hexanone	2.02 ± 0.24	-0.21 ± 0.24	-0.26 ± 0.28
**HXBE**	8	0	hexyl butanoate	1.70 ± 0.65	-0.24 ± 0.32	0.01 ± 0.45
**BBTL**	9	1	β-butyrolactone	1.65 ± 0.30	0.42 ± 0.47	-0.21 ± 0.48
**PENS**	7	0	pentanoic acid	1.41 ± 0.88	0.63 ± 0.85	0.47 ± 0.27
**NONK**	7	0	n-nonane	1.25 ± 0.64	0.63 ± 0.32	-0.23 ± 0.44
**OCTK**	7	0	n-octane	0.67 ± 0.76	-0.19 ± 0.35	-0.14 ± 0.56
**HEPK**	9	0	heptane	0.54 ± 0.34	0.17 ± 0.55	0.12 ± 0.55
**BNIM**	8	0	benzonitrile	0.52 ± 0.32	0.62 ± 0.25	0.26 ± 0.48
**EUGM**	5	0	eugenol	0.50 ± 0.23	0.21 ± 0.55	-0.67 ± 0.32
**OCAE**	8	0	octyl acetate	0.50 ± 0.30	0.51 ± 0.47	-0.52 ± 0.75
**THUT**	9	0	(–)-α-thujone	0.47 ± 0.33	-0.55 ± 0.09	-0.05 ± 0.35
**DMBM**	6	0	4-allyl-1,2-dimethoxybenzene	0.34 ± 0.06	-0.13 ± 0.42	0.40 ± 0.59
**PINT**	8	0	(+)-α-pinene	0.32 ± 0.32	0.00 ± 0.12	0.38 ± 0.24
**BDOL**	8	0	2,3-butanediol (rac)	0.32 ± 0.30	-0.15 ± 0.52	-0.32 ± 0.58
**MCHL**	7	0	4-methylcyclohexanol (rac)	0.24 ± 0.24	0.31 ± 0.13	0.00 ± 0.00

**2PPM**	9	0	2-propylphenol	0.20 ± 0.41	-0.32 ± 0.32	-0.38 ± 0.33
**MBAE**	8	0	2-methylbutyl acetate	0.10 ± 0.45	0.39 ± 0.16	0.42 ± 0.42
**EMBE**	9	0	ethyl 2-methylbutanoate	0.10 ± 0.52	0.29 ± 0.41	0.08 ± 0.77
**GEST**	5	0	geranyl acetate	0.00 ± 0.38	0.48 ± 0.40	0.32 ± 0.73
**PROA**	7	0	propanal	0.00 ± 0.25	0.25 ± 0.12	0.25 ± 0.43
**BEAM**	9	0	benzaldehyde	0.00 ± 0.02	0.11 ± 0.40	0.35 ± 0.19
**DECA**	7	0	decanal	-0.00 ± 0.43	-0.00 ± 0.23	-0.17 ± 0.65
**EM2E**	8	0	ethyl tiglate	0.00 ± 1.00	0.11 ± 0.17	0.03 ± 0.09
**LIMT**	9	0	(R)-(+)-limonene	0.00 ± 0.31	-0.86 ± 0.30	-0.51 ± 0.66
**PROS**	8	0	propanoic acid	-0.02 ± 0.31	-0.11 ± 0.38	0.05 ± 0.36
**FENT**	8	0	(1R)-(-)-fenchone	-0.10 ± 0.59	-0.01 ± 0.49	0.14 ± 0.51
**IPBM**	8	0	4-isopropylbenzaldehyde	-0.13 ± 0.74	0.34 ± 0.44	-0.08 ± 0.50
**CAST**	7	0	(S)-(+)-carvone	-0.15 ± 0.37	-0.00 ± 0.26	-0.00 ± 0.20
**CART**	7	0	(R)-(-)-carvone	-0.24 ± 0.20	-0.28 ± 0.59	0.11 ± 0.43
**EACE**	7	0	ethyl acetate	-0.25 ± 0.42	-0.62 ± 0.61	-0.42 ± 0.15
**PANM**	8	0	trans-p-propenylanisol	-0.27 ± 0.52	0.02 ± 0.38	-0.10 ± 0.48
**ALOT**	5	0	α-ionone	-0.39 ± 0.20	0.35 ± 0.03	0.61 ± 0.11
**DECL**	5	0	1-decanol	-0.49 ± 0.40	0.39 ± 0.90	-0.42 ± 0.49
**MSAM**	8	0	methylsalicylate	-0.50 ± 0.11	0.04 ± 0.48	-0.08 ± 0.51
**2EBM**	5	0	ethyl benzoate	-0.53 ± 0.67	0.65 ± 0.15	0.00 ± 0.43
**2EPM**	7	0	2-ethylphenol	-0.70 ± 0.17	-0.32 ± 0.60	-0.08 ± 0.56
**MEBM**	8	0	methoxybenzene	-0.90 ± 0.28	-0.24 ± 0.56	-0.21 ± 0.62
**BOLM**	7	0	benzyl alcohol	-0.91 ± 0.77	-0.96 ± 0.24	-0.41 ± 0.18
**ET3E**	8	0	ethyl propionate	-1.07 ± 0.55	-0.51 ± 0.38	0.32 ± 0.25
**CILT**	4	0	β-citronellol	-1.09 ± 0.59	-0.25 ± 0.57	0.03 ± 0.53
**CINT**	7	0	1,8-cineole	-1.95 ± 0.21	-0.38 ± 0.22	0.03 ± 0.36
**2MPM**	5	1	2-methylphenol	-2.66 ± 0.55	-1.15 ± 0.71	0.61 ± 0.19
