# Supplementary material for: Olfactory Receptor Responses to Pure Odorants in Drosophila melanogaster
Source: Eur J Neurosci. 2025 Mar 10;61(5):e70036. doi: 10.1111/ejn.70036 (PMC11891828; doi:10.1111/ejn.70036)
Supplement: Supplementary file 5 — Appendix Table 2 Or22a. [file EJN-61-0-s014.pdf]

Appendix_Table2_Or22a

odor code	num values	category no.	Odorant	response -2	response -4	response -6
**ET3E**	5	3	ethyl propionate	66.94 ± 12.49	37.71 ± 4.79	5.49 ± 2.17
**EMBE**	4	3	ethyl 2-methylbutanoate	54.52 ± 2.40	32.55 ± 2.05	6.13 ± 0.97
**MBAE**	4	2	2-methylbutyl acetate	37.88 ± 8.84	13.29 ± 2.72	0.50 ± 0.26
**ISOE**	5	1	isoamyl acetate	30.67 ± 4.97	4.63 ± 1.80	-0.75 ± 1.19
**EM2E**	4	2	ethyl tiglate	30.28 ± 5.93	7.04 ± 0.76	-0.16 ± 0.19
**BACE**	7	2	butyl acetate	29.88 ± 3.49	2.95 ± 0.83	-0.00 ± 0.38
**BBTL**	4	2	β-butyrolactone	28.71 ± 2.70	5.96 ± 1.02	-0.00 ± 0.01
**HEPA**	7	2	heptanal	27.68 ± 4.77	3.79 ± 0.42	0.27 ± 0.38
**M3HE**	7	2	methyl 3-hydroxyhexanoate	22.15 ± 3.22	2.97 ± 0.34	0.39 ± 0.43
**H3XL**	5	2	3-hexanol	21.25 ± 2.77	1.79 ± 1.76	-0.33 ± 0.58
**HEXN**	6	1	2-hexanone	15.30 ± 2.68	-0.66 ± 0.43	0.28 ± 0.51
**EHAE**	6	1	E2-hexenyl acetate	15.16 ± 2.38	0.31 ± 0.46	0.05 ± 0.30
**ETHE**	5	2	ethyl hexanoate	12.48 ± 3.24	9.98 ± 2.96	2.69 ± 2.90
**E3HE**	7	1	ethyl 3-hydroxyhexanoate	12.07 ± 0.29	1.10 ± 0.68	0.12 ± 0.27
**OC3N**	20	2	3-octanone	12.07 ± 4.16	1.58 ± 0.75	-0.17 ± 0.43
**PROS**	7	1	propanoic acid	11.27 ± 3.77	-0.17 ± 0.19	0.15 ± 0.33
**ESHE**	4	1	ethyl (S)-(+)-3-hydroxybutyrate	11.20 ± 1.10	0.65 ± 1.47	0.10 ± 0.22
**GVAL**	6	1	γ-valerolactone	10.40 ± 2.87	0.55 ± 0.23	0.40 ± 0.26
**HEPN**	8	1	2-heptanone	8.03 ± 0.32	-0.83 ± 0.24	0.13 ± 0.38
**HXAE**	8	1	hexyl acetate	6.69 ± 0.69	-0.26 ± 0.23	-0.38 ± 0.33
**HP2L**	6	2	2-heptanol	6.11 ± 1.42	-1.43 ± 0.29	-0.44 ± 0.11
**ZHAE**	4	0	Z3-hexenyl acetate	5.11 ± 1.17	0.15 ± 0.17	-0.32 ± 0.21
**HX3L**	7	1	1-hexen-3-ol	3.42 ± 0.72	0.19 ± 1.00	0.31 ± 0.19
**HX2L**	8	1	(±)-2-hexanol (rac)	2.93 ± 0.22	0.25 ± 0.35	0.13 ± 0.41
**BDOL**	4	1	2,3-butanediol (rac)	2.20 ± 0.35	0.16 ± 0.43	0.27 ± 0.03
**OCTN**	7	1	2-octanone	1.71 ± 0.64	-0.00 ± 0.40	0.53 ± 0.12
**Z3HL**	7	1	Z3-hexen-1-ol	1.61 ± 0.18	0.00 ± 0.39	0.34 ± 0.25
**BEAM**	7	0	benzaldehyde	1.52 ± 1.52	0.52 ± 0.52	0.14 ± 0.75
**HEPK**	4	0	heptane	1.33 ± 0.17	-0.66 ± 0.89	-0.18 ± 0.18
**OCTK**	7	0	n-octane	0.76 ± 0.34	0.00 ± 0.45	-0.26 ± 0.54
**LIMT**	7	0	(R)-(+)-limonene	0.52 ± 0.18	0.80 ± 0.43	0.06 ± 0.31
**PROA**	7	0	propanal	0.48 ± 0.31	-0.14 ± 0.25	-0.35 ± 0.14
**HPAE**	6	0	heptyl acetate	0.42 ± 0.58	0.44 ± 0.14	0.22 ± 0.44
**NONN**	5	0	2-nonanone	0.39 ± 0.29	-0.38 ± 0.38	-0.21 ± 0.66
**2EPM**	7	0	2-ethylphenol	0.37 ± 0.60	-0.66 ± 0.45	-0.23 ± 0.43
**FENT**	4	0	(1R)-(-)-fenchone	0.31 ± 0.30	0.13 ± 0.40	0.16 ± 0.18
**PANM**	5	0	trans-p-propenylanisol	0.28 ± 0.28	-0.55 ± 0.14	-0.49 ± 0.83
**THUT**	7	0	(–)-α-thujone	0.22 ± 0.28	-0.02 ± 0.54	-0.14 ± 1.00
**IPBM**	6	0	4-isopropylbenzaldehyde	0.19 ± 0.64	0.28 ± 0.52	-0.19 ± 0.54

**MCHL**	8	0	4-methylcyclohexanol (rac)	0.19 ± 0.14	0.05 ± 0.54	0.35 ± 0.22
**DECL**	7	0	1-decanol	0.16 ± 0.41	-0.12 ± 0.12	-0.18 ± 0.44
**DECA**	8	0	decanal	0.15 ± 0.29	-0.12 ± 0.08	-0.22 ± 0.42
**OCTA**	5	0	octanal	0.12 ± 0.69	-0.34 ± 0.92	0.30 ± 0.56
**BNIM**	7	0	benzonitrile	0.06 ± 0.69	0.37 ± 0.37	0.61 ± 0.25
**PRBL**	4	0	γ-propyl-γ-butyrolactone	0.05 ± 0.70	0.08 ± 0.54	-0.47 ± 0.06
**LINT**	8	0	linalool	0.03 ± 0.33	-0.01 ± 0.27	-0.26 ± 0.36
**MSAM**	4	0	methylsalicylate	0.02 ± 0.19	-0.01 ± 0.20	0.25 ± 0.33
**ALOT**	7	0	α-ionone	0.01 ± 0.67	0.31 ± 0.53	0.25 ± 0.71
**CART**	7	0	(R)-(-)-carvone	0.00 ± 0.22	0.14 ± 0.16	-0.06 ± 0.63
**NONK**	7	0	n-nonane	0.00 ± 0.25	0.35 ± 0.40	-0.00 ± 0.25
**HXBE**	5	0	hexyl butanoate	-0.00 ± 0.71	-0.11 ± 0.17	-0.02 ± 0.25
**OC3L**	4	0	3-octanol	-0.01 ± 2.89	0.05 ± 0.90	0.53 ± 0.51
**GEST**	4	0	geranyl acetate	-0.02 ± 0.42	0.18 ± 0.14	0.02 ± 0.14
**2PPM**	7	0	2-propylphenol	-0.06 ± 0.65	0.13 ± 0.62	-0.91 ± 1.23
**PINT**	5	0	(+)-α-pinene	-0.08 ± 0.53	-0.31 ± 0.60	0.46 ± 1.28
**CINT**	7	0	1,8-cineole	-0.19 ± 0.22	-0.33 ± 0.69	-0.44 ± 0.69
**DMBM**	8	0	4-allyl-1,2-dimethoxybenzene	-0.19 ± 0.26	0.08 ± 0.23	0.16 ± 0.50
**OCAE**	7	0	octyl acetate	-0.28 ± 0.35	-0.00 ± 0.39	-0.50 ± 0.70
**MEBM**	7	0	methoxybenzene	-0.32 ± 0.37	0.27 ± 0.12	-0.16 ± 0.39
**EUGM**	7	0	eugenol	-0.33 ± 0.33	-0.30 ± 0.39	-0.30 ± 0.45
**CAST**	7	0	(S)-(+)-carvone	-0.47 ± 0.39	0.23 ± 0.54	-0.42 ± 0.16
**BOLM**	7	0	benzyl alcohol	-0.53 ± 0.30	-0.00 ± 0.47	-0.42 ± 0.53
**PENS**	7	0	pentanoic acid	-0.72 ± 0.88	-0.00 ± 0.48	0.76 ± 0.59
**CILT**	4	0	β-citronellol	-0.94 ± 0.36	-1.58 ± 0.71	-0.51 ± 0.75
**HEXS**	7	0	hexanoic acid	-1.24 ± 0.69	0.09 ± 1.00	-0.49 ± 0.77
**4MPM**	4	0	4-methylphenol	-1.65 ± 0.74	0.48 ± 0.73	-0.31 ± 0.18
