# Supplementary material for: Olfactory Receptor Responses to Pure Odorants in Drosophila melanogaster
Source: Eur J Neurosci. 2025 Mar 10;61(5):e70036. doi: 10.1111/ejn.70036 (PMC11891828; doi:10.1111/ejn.70036)
Supplement: Supplementary file 6 — Appendix Table 2 Or42b. [file EJN-61-0-s006.pdf]

Appendix_Table2_Or42b

odor code	num values	category no.	Odorant	response -2	response -4	response -6
**ET3E**	21,6	2	ethyl propionate	27.50 ± 3.10	11.21 ± 1.59	0.43 ± 0.95
**ESHE**	5	1	ethyl (S)-(+)-3-hydroxybutyrate	10.62 ± 3.75	1.65 ± 0.68	0.40 ± 0.26
**HEXN**	6	0	2-hexanone	1.09 ± 1.28	-0.02 ± 0.15	-0.26 ± 0.41
**H3XL**	6	0	3-hexanol	0.94 ± 0.74	0.85 ± 0.18	0.39 ± 0.33
**4MPM**	5	0	4-methylphenol	0.63 ± 0.64	0.36 ± 0.69	-0.24 ± 0.24
**BBTL**	5	0	β-butyrolactone	0.63 ± 0.22	-0.19 ± 0.68	0.46 ± 0.24
**PROS**	5	0	propanoic acid	0.57 ± 0.22	-0.07 ± 0.07	-0.16 ± 0.05
**CILT**	5	0	β-citronellol	0.49 ± 0.59	-0.00 ± 0.58	0.20 ± 0.50
**PRBL**	5	0	γ-propyl-γ-butyrolactone	0.46 ± 0.31	-0.24 ± 0.12	-0.41 ± 0.13
**2EPM**	5	0	2-ethylphenol	0.37 ± 0.36	-0.20 ± 0.48	0.38 ± 0.49
**OCTK**	5	0	n-octane	0.35 ± 0.11	-0.24 ± 0.15	-0.50 ± 0.23
**HX2L**	5	0	(±)-2-hexanol (rac)	0.35 ± 0.74	0.07 ± 0.27	-0.42 ± 0.31
**PANM**	6	0	trans-p-propenylanisol	0.34 ± 0.33	0.19 ± 0.34	0.24 ± 0.17
**HEPK**	5	0	heptane	0.27 ± 0.27	0.61 ± 0.09	-0.00 ± 0.38
**LIMT**	6	0	(R)-(+)-limonene	0.15 ± 0.17	-0.12 ± 0.16	0.02 ± 0.30
**ALOT**	6	0	α-ionone	0.13 ± 0.79	-0.31 ± 0.16	-0.18 ± 0.21
**Z3HL**	5	0	Z3-hexen-1-ol	0.12 ± 0.12	0.00 ± 0.34	0.26 ± 0.45
**ZHAE**	4	0	Z3-hexenyl acetate	0.08 ± 0.37	0.33 ± 0.20	-0.14 ± 0.45
**HX3L**	6	0	1-hexen-3-ol	0.03 ± 0.28	0.05 ± 0.10	0.04 ± 0.21
**FENT**	4	0	(1R)-(-)-fenchone	0.01 ± 0.38	-0.21 ± 0.41	0.04 ± 0.42
**BOLM**	5	0	benzyl alcohol	-0.00 ± 0.40	0.39 ± 0.17	-0.34 ± 0.34
**LINT**	5	0	linalool	0.00 ± 0.39	0.20 ± 0.37	-0.14 ± 0.37
**PENS**	5	0	pentanoic acid	-0.00 ± 0.27	0.42 ± 0.52	0.09 ± 0.27
**DECA**	5	0	decanal	0.00 ± 0.16	0.00 ± 0.19	0.00 ± 0.20
**OC3L**	5	0	3-octanol	0.00 ± 0.00	-0.00 ± 0.71	-0.11 ± 0.53
**EUGM**	5	0	eugenol	0.00 ± 0.33	-0.17 ± 0.28	0.10 ± 0.20
**DECL**	6	0	1-decanol	-0.10 ± 0.27	-0.03 ± 0.22	-0.09 ± 0.20
**GVAL**	6	0	γ-valerolactone	-0.12 ± 0.09	-0.00 ± 0.10	-0.00 ± 0.18
**MCHL**	5	0	4-methylcyclohexanol (rac)	-0.13 ± 0.48	-0.21 ± 0.26	-0.00 ± 0.11
**DMBM**	5	0	4-allyl-1,2-dimethoxybenzene	-0.14 ± 0.14	0.26 ± 0.38	-0.14 ± 0.43
**NONN**	6	0	2-nonanone	-0.15 ± 0.37	-0.16 ± 0.39	-0.16 ± 0.22
**GEST**	4	0	geranyl acetate	-0.15 ± 0.45	-0.41 ± 0.82	-0.24 ± 0.35
**BNIM**	5	0	benzonitrile	-0.18 ± 0.61	-0.13 ± 0.48	0.00 ± 0.27
**HXAE**	5	0	hexyl acetate	-0.18 ± 0.50	-0.00 ± 0.38	-0.24 ± 0.14
**CAST**	5	0	(S)-(+)-carvone	-0.22 ± 0.43	-0.28 ± 0.20	0.24 ± 0.11
**HPAE**	6	0	heptyl acetate	-0.23 ± 0.24	-0.01 ± 0.40	-0.10 ± 0.30
**CART**	6	0	(R)-(-)-carvone	-0.23 ± 0.43	-0.23 ± 0.20	-0.02 ± 0.38
**OCAE**	5	0	octyl acetate	-0.24 ± 0.01	0.00 ± 0.13	0.36 ± 0.06
**MEBM**	5	0	methoxybenzene	-0.25 ± 0.40	0.00 ± 0.08	-0.23 ± 0.18

**THUT**	6	0	(–)-α-thujone	-0.28 ± 0.27	-0.17 ± 0.37	-0.28 ± 0.48
**2PPM**	6	0	2-propylphenol	-0.31 ± 0.11	0.20 ± 0.15	0.11 ± 0.26
**HXBE**	6	0	hexyl butanoate	-0.33 ± 0.43	-0.22 ± 0.39	0.02 ± 0.21
**OCTA**	6	0	octanal	-0.34 ± 0.10	0.61 ± 0.37	-0.30 ± 0.18
**HEXS**	5	0	hexanoic acid	-0.36 ± 0.51	-0.31 ± 0.10	0.34 ± 0.42
**NONK**	6	0	n-nonane	-0.37 ± 0.21	-0.28 ± 0.58	0.03 ± 0.47
**IPBM**	6	0	4-isopropylbenzaldehyde	-0.40 ± 0.17	0.08 ± 0.30	-0.05 ± 0.39
**PINT**	6	0	(+)-α-pinene	-0.40 ± 0.27	0.00 ± 0.33	0.26 ± 0.27
**EHAE**	6	0	E2-hexenyl acetate	-0.42 ± 0.22	-0.50 ± 0.43	-0.31 ± 0.08
**CINT**	6	0	1,8-cineole	-0.52 ± 0.26	-0.06 ± 0.23	0.14 ± 0.37
**PROA**	6	0	propanal	-0.52 ± 0.90	0.42 ± 0.39	0.07 ± 0.43
**BEAM**	6	0	benzaldehyde	-0.52 ± 0.52	-0.14 ± 0.40	0.47 ± 0.28
**BJOT**	5	1	β-ionone	-0.59 ± 0.29	-0.63 ± 0.17	0.15 ± 0.45
**E3HE**	5	0	ethyl 3-hydroxyhexanoate	-0.59 ± 0.43	-0.10 ± 0.43	0.33 ± 0.11
**EMBE**	5	0	ethyl 2-methylbutanoate	-0.67 ± 0.19	-0.45 ± 0.65	-0.09 ± 0.16
**MSAM**	4	0	methylsalicylate	-0.78 ± 0.67	-0.21 ± 0.63	0.15 ± 0.49
**HEPA**	6	0	heptanal	-0.92 ± 0.18	-0.29 ± 0.61	0.26 ± 0.28
**OCTN**	6	1	2-octanone	-1.29 ± 0.38	-0.29 ± 0.51	-0.20 ± 0.22
**M3HE**	5	1	methyl 3-hydroxyhexanoate	-1.46 ± 0.23	-0.54 ± 0.76	0.27 ± 0.40
**ISOE**	6	1	isoamyl acetate	-1.62 ± 0.43	0.21 ± 0.78	0.16 ± 0.44
**MBAE**	4	0	2-methylbutyl acetate	-1.80 ± 0.90	0.17 ± 0.42	-0.21 ± 0.30
**BDOL**	4	0	2,3-butanediol (rac)	-2.30 ± 0.32	-0.17 ± 0.19	0.33 ± 0.24
**EM2E**	4	1	ethyl tiglate	-2.68 ± 0.35	0.17 ± 0.20	0.15 ± 0.18
**HP2L**	6	2	2-heptanol	-3.40 ± 0.20	-1.62 ± 0.33	-0.00 ± 0.12
**HEPN**	5	2	2-heptanone	-3.78 ± 0.15	-1.05 ± 0.16	-0.15 ± 0.27
**BACE**	6	1	butyl acetate	-3.81 ± 0.33	0.11 ± 0.50	-0.26 ± 0.19
