# Supplementary material for: Olfactory Receptor Responses to Pure Odorants in Drosophila melanogaster
Source: Eur J Neurosci. 2025 Mar 10;61(5):e70036. doi: 10.1111/ejn.70036 (PMC11891828; doi:10.1111/ejn.70036)
Supplement: Supplementary file 7 — Appendix Table 2 Or47a. [file EJN-61-0-s008.pdf]

Appendix_Table2_Or47a

odor code	num values	category no.	Odorant	response -2	response -4	response -6
**PACE**	11	3	pentyl acetate	68.98 ± 10.29	33.63 ± 3.35	7.05 ± 1.53
**MEHE**	6	2	methyl hexanoate	53.17 ± 11.66	16.24 ± 3.31	0.61 ± 0.19
**PRAE**	10	2	propyl acetate	50.92 ± 6.85	6.40 ± 1.08	0.17 ± 0.17
**ZHAE**	8	2	Z3-hexenyl acetate	47.91 ± 9.37	11.57 ± 1.83	0.31 ± 0.46
**BACE**	10	2	butyl acetate	41.91 ± 5.05	10.09 ± 1.44	-0.12 ± 0.27
**IBAE**	7	2	isobutyl acetate	41.16 ± 12.22	7.88 ± 2.07	0.23 ± 0.11
**M3HE**	7	2	methyl 3-hydroxyhexanoate	32.09 ± 13.11	6.38 ± 3.36	-0.18 ± 0.18
**EMSE**	7	2	ethyl 3-methylsulfanylpropanoate	29.50 ± 7.49	4.51 ± 1.16	0.37 ± 0.49
**HXAE**	9	2	hexyl acetate	26.33 ± 9.91	6.79 ± 2.80	0.00 ± 0.28
**MBAE**	8	2	2-methylbutyl acetate	25.58 ± 6.11	1.60 ± 0.64	0.12 ± 0.15
**HEPN**	9	2	2-heptanone	25.32 ± 7.39	4.03 ± 2.42	-0.03 ± 0.49
**HEXN**	8	1	2-hexanone	22.21 ± 3.53	0.44 ± 0.57	0.24 ± 0.18
**ISOE**	11	2	isoamyl acetate	17.07 ± 4.07	1.22 ± 0.43	0.11 ± 0.30
**OCTN**	10	1	2-octanone	15.44 ± 1.72	0.46 ± 0.12	-0.10 ± 0.30
**HX2L**	9	1	(±)-2-hexanol (rac)	14.42 ± 4.06	0.36 ± 0.35	0.02 ± 0.26
**HX3L**	11	1	1-hexen-3-ol	13.45 ± 1.75	0.11 ± 0.37	0.23 ± 0.16
**OC3L**	36,10	1	3-octanol	13.43 ± 6.32	0.58 ± 0.34	-0.22 ± 0.13
**3HXN**	6	1	3-hexanone	12.82 ± 1.38	0.16 ± 0.20	0.15 ± 0.18
**E2BE**	6	1	ethyl trans-2-butenoate	11.89 ± 1.03	-0.31 ± 0.13	-0.07 ± 0.28
**H3XL**	11	1	3-hexanol	11.13 ± 3.37	0.18 ± 0.24	0.06 ± 0.12
**EHAE**	8	1	E2-hexenyl acetate	10.66 ± 2.33	0.22 ± 0.46	0.32 ± 0.12
**E3HE**	7	1	ethyl 3-hydroxyhexanoate	9.76 ± 3.76	-0.16 ± 0.55	0.33 ± 0.25
**MTPL**	6	1	3-(methylthio)-1-propanol	9.20 ± 6.66	-0.35 ± 0.83	0.15 ± 0.47
**EACE**	9	1	ethyl acetate	8.64 ± 3.62	0.20 ± 0.18	0.04 ± 0.35
**O13L**	9	1	1-octen-3-ol	7.70 ± 1.47	0.00 ± 0.38	0.14 ± 0.42
**HP2L**	8	1	2-heptanol	7.29 ± 2.24	0.21 ± 0.33	-0.09 ± 0.22
**HPAE**	8	1	heptyl acetate	7.21 ± 1.38	-0.30 ± 0.35	-0.20 ± 0.19
**ESHE**	10	1	ethyl (S)-(+)-3-hydroxybutyrate	6.70 ± 3.51	0.21 ± 0.25	-0.05 ± 0.35
**H21L**	7	1	trans-2-hexen-1-ol	6.49 ± 3.31	-0.23 ± 0.23	0.14 ± 0.29
**NONN**	11	1	2-nonanone	6.08 ± 2.65	0.25 ± 0.25	0.07 ± 0.42
**ET3E**	11	1	ethyl propionate	4.18 ± 1.88	0.25 ± 0.23	0.24 ± 0.17
**Z3HL**	7	0	Z3-hexen-1-ol	2.57 ± 1.68	0.00 ± 0.08	0.29 ± 0.36
**HEPA**	11	1	heptanal	2.47 ± 1.28	0.28 ± 0.43	0.18 ± 0.25
**BNIM**	7	0	benzonitrile	1.96 ± 0.90	-0.09 ± 0.63	-0.23 ± 0.40
**PE3L**	6	0	1-penten-3-ol	1.89 ± 1.47	0.39 ± 0.16	0.10 ± 0.28
**HEXL**	9	0	1-hexanol	1.31 ± 1.52	-0.18 ± 0.34	0.17 ± 0.42
**EMBE**	10	0	ethyl 2-methylbutanoate	0.87 ± 0.89	0.01 ± 0.19	-0.09 ± 0.21
**BUTN**	9	0	2-butanone	0.86 ± 0.17	-0.00 ± 0.46	0.22 ± 0.21
**DESE**	6	1	diethyl succinate	0.80 ± 3.46	-1.29 ± 1.21	-0.11 ± 0.63
**HEXA**	9	0	hexanal	0.75 ± 0.49	-0.20 ± 0.73	0.11 ± 0.29

**PRBL**	10	0	γ-propyl-γ-butyrolactone	0.51 ± 0.40	0.19 ± 0.09	0.04 ± 0.20
**HXHE**	7	0	hexyl hexanoate	0.37 ± 0.47	-0.30 ± 0.52	-0.19 ± 0.20
**ALOT**	11	0	α-ionone	0.30 ± 0.28	0.07 ± 0.38	0.00 ± 0.35
**2EPM**	8	0	2-ethylphenol	0.28 ± 0.21	0.40 ± 0.20	0.04 ± 0.25
**OCTA**	11	0	octanal	0.24 ± 0.79	0.14 ± 0.25	0.00 ± 0.39
**2EBM**	9	0	ethyl benzoate	0.19 ± 0.22	0.00 ± 0.34	0.33 ± 0.23
**BDOL**	8	0	2,3-butanediol (rac)	0.19 ± 0.21	0.19 ± 0.19	0.22 ± 0.37
**HEPK**	10	0	heptane	0.18 ± 0.10	-0.20 ± 0.16	0.25 ± 0.16
**CAST**	8	0	(S)-(+)-carvone	0.18 ± 0.40	-0.06 ± 0.23	0.21 ± 0.19
**CILT**	10	0	β-citronellol	0.16 ± 0.36	0.08 ± 0.13	-0.09 ± 0.27
**BBTL**	10	0	β-butyrolactone	0.14 ± 0.15	0.08 ± 0.25	-0.09 ± 0.25
**2PPM**	11	0	2-propylphenol	0.14 ± 0.38	0.23 ± 0.24	0.18 ± 0.07
**EUGM**	7	0	eugenol	0.12 ± 0.58	0.29 ± 0.13	-0.17 ± 0.32
**NONK**	10	0	n-nonane	0.12 ± 0.58	-0.15 ± 0.15	0.00 ± 0.14
**LIMT**	11	0	(R)-(+)-limonene	0.10 ± 0.43	0.17 ± 0.22	0.03 ± 0.35
**HEXS**	8	0	hexanoic acid	0.09 ± 0.26	0.22 ± 0.19	0.28 ± 0.30
**THUT**	11	0	(–)-α-thujone	0.09 ± 0.51	0.00 ± 0.21	-0.02 ± 0.24
**DECL**	11	0	1-decanol	0.09 ± 0.40	0.08 ± 0.29	0.30 ± 0.25
**PANM**	11	0	trans-p-propenylanisol	0.05 ± 0.36	0.02 ± 0.21	0.15 ± 0.19
**PENS**	8	0	pentanoic acid	0.03 ± 0.25	0.27 ± 0.37	-0.02 ± 0.22
**EM2E**	8	0	ethyl tiglate	0.00 ± 0.00	0.12 ± 0.20	-0.13 ± 0.43
**PINT**	11	0	(+)-α-pinene	0.00 ± 0.00	0.14 ± 0.34	-0.20 ± 0.14
**DDEL**	6	0	δ-decalactone	0.00 ± 0.00	0.00 ± 0.00	0.00 ± 0.00
**DMBM**	9	0	4-allyl-1,2-dimethoxybenzene	0.00 ± 0.26	0.00 ± 0.16	-0.30 ± 0.13
**OCAE**	7	0	octyl acetate	0.00 ± 0.95	0.20 ± 0.26	0.18 ± 0.14
**MCHL**	9	0	4-methylcyclohexanol (rac)	0.00 ± 0.00	0.23 ± 0.23	0.46 ± 0.20
**LIOL2**	7	0	linalool oxide (peak2)	0.00 ± 0.18	0.06 ± 0.06	-0.26 ± 0.41
**HXBE**	11	0	hexyl butanoate	-0.07 ± 0.26	0.21 ± 0.55	-0.12 ± 0.27
**4MPM**	10	0	4-methylphenol	-0.08 ± 0.35	0.11 ± 0.31	0.23 ± 0.31
**IPBM**	8	0	4-isopropylbenzaldehyde	-0.09 ± 0.13	0.38 ± 0.14	0.02 ± 0.26
**CART**	10	0	(R)-(-)-carvone	-0.11 ± 0.30	0.00 ± 0.21	-0.09 ± 0.19
**3CAT**	7	0	3-carene	-0.14 ± 0.31	0.18 ± 0.40	0.19 ± 0.13
**OCTK**	8	0	n-octane	-0.14 ± 0.28	0.13 ± 0.33	0.05 ± 0.20
**GEST**	8	0	geranyl acetate	-0.16 ± 0.25	0.14 ± 0.24	0.07 ± 0.07
**PROS**	7	0	propanoic acid	-0.17 ± 0.28	0.26 ± 0.15	0.00 ± 0.17
**BEAM**	11	0	benzaldehyde	-0.25 ± 0.43	0.24 ± 0.43	0.11 ± 0.30
**MEBM**	7	0	methoxybenzene	-0.27 ± 0.20	0.42 ± 0.42	0.70 ± 0.61
**BOLM**	8	0	benzyl alcohol	-0.28 ± 0.34	0.00 ± 0.11	-0.22 ± 0.31
**2MPM**	9	0	2-methylphenol	-0.35 ± 0.35	-0.15 ± 0.32	0.23 ± 0.08
**PROA**	10	0	propanal	-0.36 ± 0.38	0.20 ± 0.11	0.07 ± 0.27
**CINT**	10	0	1,8-cineole	-0.43 ± 0.17	0.27 ± 0.13	0.06 ± 0.14
**PENA**	9	0	pentanal	-0.52 ± 0.39	-0.24 ± 0.24	0.20 ± 0.17

**DECA**	9	0	decanal	-0.54 ± 0.36	-0.23 ± 0.47	0.00 ± 0.54
**GVAL**	8	0	γ-valerolactone	-0.58 ± 0.39	0.34 ± 0.19	0.08 ± 0.30
**LINT**	9	0	linalool	-0.60 ± 0.27	-0.17 ± 0.21	0.46 ± 0.55
**LIOL1**	7	0	linalool oxide (peak1)	-0.65 ± 0.61	-0.36 ± 0.17	-0.53 ± 0.26
**FENT**	8	0	(1R)-(-)-fenchone	-0.69 ± 0.09	-0.45 ± 0.14	0.05 ± 0.26
**MSAM**	8	0	methylsalicylate	-0.71 ± 0.36	-0.22 ± 0.23	0.25 ± 0.16
**TERT**	9	1	a-terpineole	-1.86 ± 0.61	-0.19 ± 0.19	0.17 ± 0.51
