# Supplementary material for: Olfactory Receptor Responses to Pure Odorants in Drosophila melanogaster
Source: Eur J Neurosci. 2025 Mar 10;61(5):e70036. doi: 10.1111/ejn.70036 (PMC11891828; doi:10.1111/ejn.70036)
Supplement: Supplementary file 8 — Appendix Table 2 Or56a. [file EJN-61-0-s005.pdf]

Appendix_Table2_Or56a

odor code	num values	category no.	Odorant	response -2	response -4	response -6
**2MNL**	14	3	(±)-geosmin	23.26 ± 10.04	17.37 ± 3.17	1.93 ± 0.48
**TERT**	4	0	a-terpineole	1.35 ± 0.35	0.08 ± 0.27	0.08 ± 0.29
**ZHAE**	3	0	Z3-hexenyl acetate	1.15 ± 0.93	-0.49 ± 1.05	0.79 ± 0.09
**E3HE**	3	0	ethyl 3-hydroxyhexanoate	0.83 ± 0.16	0.56 ± 0.11	-0.20 ± 0.90
**HXAE**	5	0	hexyl acetate	0.75 ± 0.43	-0.53 ± 0.69	-0.00 ± 0.86
**OCTN**	6	0	2-octanone	0.68 ± 0.28	-0.28 ± 0.42	-0.15 ± 0.09
**ET3E**	4	0	ethyl propionate	0.62 ± 0.06	0.70 ± 0.42	-0.08 ± 0.49
**OCAE**	3	0	octyl acetate	0.62 ± 0.14	-0.43 ± 0.23	-0.38 ± 1.15
**PINT**	4	0	(+)-α-pinene	0.58 ± 0.19	0.22 ± 0.64	-0.07 ± 0.64
**2EPM**	6	0	2-ethylphenol	0.52 ± 0.16	0.06 ± 0.47	-0.77 ± 1.11
**FENT**	3	0	(1R)-(-)-fenchone	0.51 ± 0.34	1.02 ± 0.37	0.48 ± 0.09
**BDOL**	3	0	2,3-butanediol (rac)	0.50 ± 0.36	0.70 ± 0.23	0.90 ± 0.17
**HEPK**	3	0	heptane	0.47 ± 0.08	-0.13 ± 0.65	-0.15 ± 0.66
**HEXS**	6	0	hexanoic acid	0.46 ± 0.05	-0.30 ± 0.63	0.35 ± 0.64
**PROS**	3	0	propanoic acid	0.39 ± 0.87	0.39 ± 0.06	-1.12 ± 0.38
**GEST**	3	0	geranyl acetate	0.36 ± 0.14	-0.79 ± 0.02	-0.00 ± 0.63
**DMBM**	5	0	4-allyl-1,2-dimethoxybenzene	0.35 ± 0.50	1.01 ± 0.17	-0.73 ± 0.52
**BUTN**	4	0	2-butanone	0.30 ± 0.06	0.18 ± 0.08	-0.02 ± 0.21
**Z3HL**	3	0	Z3-hexen-1-ol	0.28 ± 0.62	-0.47 ± 0.83	0.53 ± 0.38
**EACE**	4	0	ethyl acetate	0.27 ± 0.10	0.20 ± 0.25	-0.12 ± 0.27
**PENA**	4	0	pentanal	0.26 ± 0.07	-0.21 ± 0.48	0.63 ± 0.20
**PRBL**	3	0	γ-propyl-γ-butyrolactone	0.25 ± 0.49	-0.61 ± 0.13	-0.50 ± 0.06
**EHAE**	6	0	E2-hexenyl acetate	0.23 ± 0.32	-0.08 ± 0.30	-0.04 ± 0.47
**BEAM**	5	0	benzaldehyde	0.22 ± 0.17	0.77 ± 0.21	0.37 ± 0.37
**EUGM**	3	0	eugenol	0.22 ± 0.18	0.57 ± 0.14	1.00 ± 0.94
**BOLM**	6	0	benzyl alcohol	0.21 ± 0.74	-0.21 ± 0.87	0.19 ± 0.54
**LIMT**	5	0	(R)-(+)-limonene	0.21 ± 0.10	-0.56 ± 0.06	0.19 ± 0.29
**H3XL**	4	0	3-hexanol	0.21 ± 0.45	-0.57 ± 0.31	-0.42 ± 0.25
**MCHL**	5	0	4-methylcyclohexanol (rac)	0.20 ± 0.52	0.13 ± 0.29	-0.00 ± 0.33
**OCTA**	4	0	octanal	0.13 ± 0.37	-0.49 ± 0.31	0.32 ± 0.48
**MSAM**	3	0	methylsalicylate	0.11 ± 0.18	0.16 ± 0.35	0.25 ± 0.30
**HX2L**	5	0	(±)-2-hexanol (rac)	0.11 ± 0.92	0.24 ± 0.69	-0.75 ± 0.18
**2MPM**	4	0	2-methylphenol	0.11 ± 0.11	0.22 ± 0.24	-0.14 ± 0.43
**PELM**	5	0	2-phenylethanol	0.11 ± 0.14	-0.00 ± 0.35	0.35 ± 0.23
**OCTK**	6	0	n-octane	0.10 ± 0.40	-0.11 ± 0.64	-0.08 ± 0.67
**BNIM**	3	0	benzonitrile	0.08 ± 0.46	0.62 ± 0.39	-0.41 ± 0.96
**HEXA**	4	0	hexanal	0.07 ± 0.08	0.36 ± 0.22	-0.37 ± 0.18
**CART**	6	0	(R)-(-)-carvone	0.05 ± 0.62	0.61 ± 0.23	0.38 ± 0.43
**THUT**	5	0	(–)-α-thujone	0.04 ± 0.14	-0.34 ± 0.07	-0.44 ± 0.59

**PRAE**	4	0	propyl acetate	0.03 ± 0.35	0.06 ± 0.22	-0.24 ± 0.19
**O13L**	4	0	1-octen-3-ol	0.02 ± 0.28	-0.06 ± 0.32	-0.00 ± 0.19
**HPAE**	6	0	heptyl acetate	-0.04 ± 0.36	-0.57 ± 0.63	0.12 ± 0.45
**2EBM**	4	0	ethyl benzoate	-0.04 ± 0.32	-0.17 ± 0.31	-0.35 ± 0.50
**HP2L**	6	0	2-heptanol	-0.04 ± 0.15	-0.17 ± 0.34	-0.26 ± 0.38
**NONK**	6	0	n-nonane	-0.05 ± 0.34	0.12 ± 0.40	0.20 ± 0.43
**GVAL**	6	0	γ-valerolactone	-0.08 ± 0.45	-0.04 ± 0.31	0.45 ± 0.69
**2PPM**	5	0	2-propylphenol	-0.08 ± 0.20	0.59 ± 0.49	0.19 ± 0.38
**PROA**	6	0	propanal	-0.08 ± 0.32	-0.06 ± 0.19	-0.41 ± 0.28
**HEXN**	6	0	2-hexanone	-0.11 ± 0.13	0.46 ± 0.14	0.15 ± 0.33
**IPBM**	6	0	4-isopropylbenzaldehyde	-0.14 ± 0.09	-0.33 ± 0.17	0.27 ± 0.29
**CINT**	6	0	1,8-cineole	-0.14 ± 0.19	-0.05 ± 0.52	0.59 ± 0.21
**ESHE**	3	0	ethyl (S)-(+)-3-hydroxybutyrate	-0.16 ± 0.19	0.29 ± 0.54	0.35 ± 0.39
**HEXL**	4	0	1-hexanol	-0.16 ± 0.29	0.19 ± 0.42	0.04 ± 0.37
**HX3L**	5	0	1-hexen-3-ol	-0.17 ± 0.50	0.37 ± 0.05	-0.22 ± 0.11
**PENS**	6	0	pentanoic acid	-0.19 ± 0.51	0.56 ± 0.15	-0.34 ± 0.97
**ISOE**	4	0	isoamyl acetate	-0.21 ± 0.30	1.15 ± 0.27	-0.01 ± 0.72
**PENM**	4	0	1-phenylethanone	-0.21 ± 0.20	0.08 ± 0.15	-0.35 ± 0.04
**HXBE**	4	0	hexyl butanoate	-0.22 ± 0.50	0.31 ± 0.20	0.60 ± 0.27
**HEPN**	5	0	2-heptanone	-0.23 ± 0.48	-0.60 ± 0.76	-0.29 ± 0.81
**DECA**	5	0	decanal	-0.26 ± 0.60	-0.08 ± 0.30	0.51 ± 0.64
**BACE**	6	0	butyl acetate	-0.27 ± 0.13	0.47 ± 0.29	0.30 ± 0.17
**PACE**	4	0	pentyl acetate	-0.29 ± 0.20	-0.11 ± 0.32	-0.01 ± 0.43
**NONN**	4	0	2-nonanone	-0.29 ± 0.29	0.09 ± 0.54	0.35 ± 0.45
**M3HE**	3	0	methyl 3-hydroxyhexanoate	-0.30 ± 0.19	0.31 ± 0.34	-0.27 ± 1.37
**LINT**	5	0	linalool	-0.33 ± 0.37	0.48 ± 0.80	1.03 ± 0.52
**DECL**	5	0	1-decanol	-0.33 ± 0.07	-0.06 ± 0.57	0.46 ± 0.18
**OC3L**	3	0	3-octanol	-0.34 ± 0.20	-0.33 ± 0.86	-0.74 ± 0.03
**BEDN**	5	0	2,3-butanedione	-0.34 ± 0.47	0.35 ± 0.23	-0.67 ± 0.66
**CAST**	6	0	(S)-(+)-carvone	-0.35 ± 0.57	-0.17 ± 0.87	0.39 ± 1.00
**HEPA**	5	0	heptanal	-0.44 ± 0.23	-0.45 ± 0.09	-0.07 ± 0.15
**ETBE**	5	0	ethyl butyrate	-0.46 ± 0.10	-0.00 ± 0.14	-0.43 ± 0.49
**ALOT**	5	0	α-ionone	-0.47 ± 0.23	0.23 ± 0.43	-0.00 ± 0.33
**MEBM**	3	0	methoxybenzene	-0.50 ± 0.02	0.09 ± 0.86	-1.03 ± 0.24
**CILT**	3	0	β-citronellol	-0.52 ± 0.40	-0.64 ± 0.32	-0.53 ± 0.12
**4MPM**	3	0	4-methylphenol	-0.60 ± 0.16	-0.63 ± 0.56	-0.00 ± 0.45
**EMBE**	3	0	ethyl 2-methylbutanoate	-0.60 ± 0.34	0.03 ± 0.17	-0.44 ± 0.10
**BBTL**	3	0	β-butyrolactone	-0.66 ± 0.01	0.33 ± 0.48	-0.16 ± 0.55
**PANM**	4	0	trans-p-propenylanisol	-0.67 ± 0.11	-0.17 ± 0.43	-0.05 ± 0.74
**MBAE**	3	0	2-methylbutyl acetate	-0.78 ± 0.40	-0.25 ± 0.87	0.89 ± 1.60
**EM2E**	3	0	ethyl tiglate	-0.83 ± 0.03	-0.39 ± 0.05	0.62 ± 1.15
