# Supplementary material for: Olfactory Receptor Responses to Pure Odorants in Drosophila melanogaster
Source: Eur J Neurosci. 2025 Mar 10;61(5):e70036. doi: 10.1111/ejn.70036 (PMC11891828; doi:10.1111/ejn.70036)
Supplement: Supplementary file 9 — Appendix Table 2 Or92a. [file EJN-61-0-s004.pdf]

Appendix_Table2_Or92a

odor code	num values	category no.	Odorant	response -2	response -4	response -6
**BEDN**	8	3	2,3-butanedione	42.92 ± 14.07	21.72 ± 6.40	4.94 ± 1.45
**BDOL**	5	2	2,3-butanediol (rac)	25.54 ± 4.59	10.84 ± 1.57	-0.01 ± 1.14
**HX2L**	24,8	1	(±)-2-hexanol (rac)	9.46 ± 2.67	0.31 ± 0.12	-0.12 ± 0.23
**BUTN**	10	1	2-butanone	8.52 ± 2.22	0.35 ± 0.70	0.18 ± 0.41
**3HXN**	10	1	3-hexanone	8.39 ± 2.56	0.04 ± 1.31	0.06 ± 0.80
**ET3E**	6	1	ethyl propionate	8.03 ± 2.93	-0.32 ± 0.59	-0.26 ± 0.44
**PE3L**	10	0	1-penten-3-ol	7.25 ± 5.96	0.73 ± 0.66	0.45 ± 0.48
**ESHE**	12	1	ethyl (S)-(+)-3-hydroxybutyrate	5.88 ± 3.21	-0.30 ± 0.57	-0.14 ± 0.65
**ISOE**	6	0	isoamyl acetate	2.32 ± 2.76	0.42 ± 0.47	-0.14 ± 0.37
**OCTA**	6	0	octanal	1.64 ± 1.91	-0.69 ± 0.37	-0.19 ± 0.42
**MBAE**	9	0	2-methylbutyl acetate	1.52 ± 1.65	0.35 ± 0.64	-0.00 ± 0.91
**FENT**	9	0	(1R)-(-)-fenchone	1.33 ± 0.58	-0.62 ± 0.75	-0.00 ± 1.17
**MEHE**	10	1	methyl hexanoate	1.19 ± 1.43	0.00 ± 1.58	1.02 ± 0.65
**EM2E**	9	0	ethyl tiglate	1.13 ± 2.64	0.46 ± 1.24	-0.27 ± 0.61
**MTPL**	10	0	3-(methylthio)-1-propanol	1.12 ± 2.30	0.05 ± 0.79	-0.34 ± 0.63
**ETBE**	8	0	ethyl butyrate	1.09 ± 2.25	0.50 ± 0.92	-0.09 ± 0.56
**LIOL2**	10	0	linalool oxide (peak2)	0.76 ± 0.53	0.21 ± 0.90	-0.23 ± 0.49
**BBTL**	12	1	β-butyrolactone	0.76 ± 0.75	0.04 ± 0.32	0.47 ± 0.45
**HXBE**	6	0	hexyl butanoate	0.66 ± 1.77	-0.50 ± 0.41	0.80 ± 0.59
**PELM**	8	0	2-phenylethanol	0.65 ± 0.51	0.08 ± 0.55	0.24 ± 0.80
**PANM**	6	0	trans-p-propenylanisol	0.53 ± 0.52	0.16 ± 0.60	-0.49 ± 0.49
**ETHS**	9	0	2-ethylhexanoic acid	0.50 ± 0.48	-0.33 ± 0.37	-0.27 ± 0.98
**MCHL**	3	0	4-methylcyclohexanol (rac)	0.49 ± 0.00	-0.07 ± 0.25	0.38 ± 0.47
**MEBM**	10	0	methoxybenzene	0.49 ± 0.83	0.11 ± 0.55	-0.34 ± 0.69
**HX3L**	11	0	1-hexen-3-ol	0.47 ± 0.60	0.15 ± 0.27	-0.30 ± 0.58
**E3HE**	10	0	ethyl 3-hydroxyhexanoate	0.47 ± 0.46	0.08 ± 0.65	-0.07 ± 0.64
**LIOL1**	10	0	linalool oxide (peak1)	0.44 ± 1.39	0.00 ± 0.42	0.06 ± 0.64
**APNT**	9	0	α-pinene	0.41 ± 0.51	-0.97 ± 0.51	0.38 ± 0.62
**HEPK**	12	0	heptane	0.39 ± 0.86	-0.03 ± 0.45	-0.28 ± 0.53
**LIMT**	11	0	(R)-(+)-limonene	0.37 ± 0.43	-0.28 ± 0.48	-0.58 ± 0.39
**OC3L**	12	1	3-octanol	0.35 ± 1.20	0.41 ± 0.89	0.52 ± 0.52
**PACE**	10	0	pentyl acetate	0.34 ± 1.04	-0.23 ± 0.24	-0.58 ± 0.42
**ETAS**	9	0	ethanoic acid	0.24 ± 0.97	-0.44 ± 0.30	-0.00 ± 0.65
**HXAE**	8	0	hexyl acetate	0.20 ± 0.46	0.04 ± 0.54	-0.26 ± 0.29
**DECA**	8	0	decanal	0.15 ± 0.38	0.25 ± 0.38	0.05 ± 0.32
**MCHL1**	5	0	4-methylcyclohexanol (peak1)	0.12 ± 0.35	0.41 ± 0.36	-0.30 ± 0.29
**EUGM**	10	0	eugenol	0.01 ± 0.44	0.60 ± 0.19	0.59 ± 0.19
**NONN**	6	0	2-nonanone	0.01 ± 0.52	0.25 ± 0.46	0.09 ± 0.35
**THUT**	11	0	(–)-α-thujone	-0.00 ± 0.58	0.40 ± 0.45	-0.57 ± 0.43

**ZHAE**	9	0	Z3-hexenyl acetate	0.00 ± 0.97	-0.36 ± 0.62	0.60 ± 0.99
**HEPN**	8	0	2-heptanone	0.00 ± 0.39	-0.78 ± 0.33	0.16 ± 0.18
**HEPA**	11	0	heptanal	-0.00 ± 0.77	-0.20 ± 0.40	0.25 ± 0.48
**ACEA**	9	0	acetaldehyde	0.00 ± 1.01	0.70 ± 0.20	0.36 ± 0.36
**ALOT**	11	0	α-ionone	-0.00 ± 0.37	-0.50 ± 0.58	0.56 ± 0.48
**LINT**	8	0	linalool	-0.06 ± 0.44	-0.21 ± 0.30	0.18 ± 0.24
**2PPM**	11	0	2-propylphenol	-0.11 ± 0.64	-1.02 ± 0.89	-0.28 ± 0.78
**TERT**	10	0	a-terpineole	-0.15 ± 0.57	-0.19 ± 0.37	-0.15 ± 0.47
**DMBM**	8	0	4-allyl-1,2-dimethoxybenzene	-0.16 ± 0.23	0.08 ± 0.29	-0.16 ± 0.36
**NERL**	9	0	nerol	-0.17 ± 0.65	0.22 ± 0.22	0.26 ± 1.15
**OCAE**	10	0	octyl acetate	-0.21 ± 0.40	0.41 ± 0.56	-0.34 ± 0.68
**PINT**	6	0	(+)-α-pinene	-0.23 ± 0.88	0.54 ± 0.46	-0.21 ± 0.49
**CILT**	12	0	β-citronellol	-0.25 ± 0.40	-0.14 ± 0.60	0.12 ± 0.55
**MCHL2**	5	0	4-methylcyclohexanol (peak2)	-0.37 ± 0.23	-0.00 ± 0.56	0.23 ± 0.14
**BNIM**	10	0	benzonitrile	-0.37 ± 0.67	-0.36 ± 0.51	-0.00 ± 0.74
**Z3HL**	10	0	Z3-hexen-1-ol	-0.42 ± 0.68	-0.41 ± 0.45	0.38 ± 0.81
**4MPM**	12	0	4-methylphenol	-0.43 ± 0.71	0.00 ± 0.43	-0.51 ± 0.45
**IPES**	9	0	isopentanoic acid	-0.45 ± 0.81	-0.00 ± 0.63	0.66 ± 0.66
**HEXA**	10	0	hexanal	-0.47 ± 0.97	0.03 ± 0.53	0.03 ± 0.50
**BEAM**	11	0	benzaldehyde	-0.48 ± 1.09	-0.00 ± 0.60	0.26 ± 0.74
**DECL**	11	0	1-decanol	-0.51 ± 0.51	-0.00 ± 0.06	0.00 ± 0.59
**PROS**	10	0	propanoic acid	-0.54 ± 1.01	-0.07 ± 0.57	-0.04 ± 0.58
**EMBE**	12	0	ethyl 2-methylbutanoate	-0.55 ± 1.25	0.08 ± 1.11	-0.03 ± 0.66
**M3HE**	10	1	methyl 3-hydroxyhexanoate	-0.55 ± 0.79	0.49 ± 0.49	-0.35 ± 0.79
**DESE**	10	0	diethyl succinate	-0.70 ± 0.99	0.27 ± 0.64	-0.07 ± 0.59
**PRBL**	12	0	γ-propyl-γ-butyrolactone	-0.70 ± 0.39	0.24 ± 0.71	-0.17 ± 0.44
**E2BE**	10	0	ethyl trans-2-butenoate	-0.85 ± 1.03	-0.01 ± 1.11	-0.74 ± 0.69
**PENM**	10	0	1-phenylethanone	-0.92 ± 0.50	-0.00 ± 0.71	-0.10 ± 0.46
**H3XL**	6	0	3-hexanol	-0.97 ± 1.73	0.07 ± 0.53	-0.10 ± 0.61
**GEST**	9	0	geranyl acetate	-0.99 ± 0.77	-0.55 ± 0.64	0.51 ± 0.51
**MSAM**	9	0	methylsalicylate	-1.31 ± 2.26	-1.65 ± 0.49	0.22 ± 0.64
**PRAE**	10	1	propyl acetate	-3.43 ± 1.21	0.45 ± 0.32	-0.15 ± 0.59
