# Supplementary figures and images for: Olfactory Receptor Responses to Pure Odorants in Drosophila melanogaster
Source: Eur J Neurosci. 2025 Mar 10;61(5):e70036. doi: 10.1111/ejn.70036 (PMC11891828; doi:10.1111/ejn.70036)

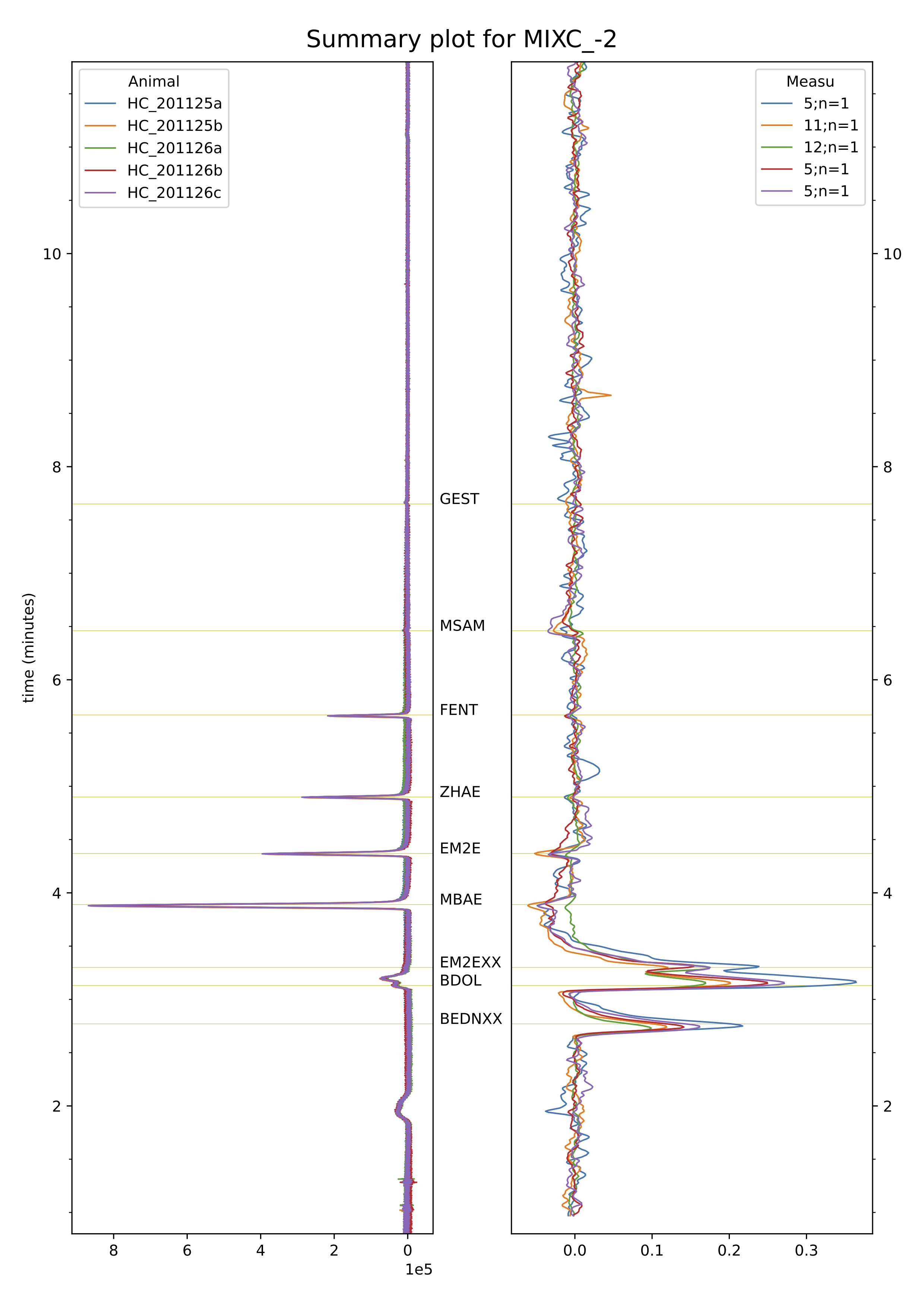

Supplement: Supplementary file 10 — Fig. S1 The summary plot for Or92a shows the time traces of the GC‐FID and of the calcium recording to a mixture of odorants (here: MixC at a dilution of 10−2). In this experiment, the nonpolar column was used (for comparison: the same MixC, separated by the polar column is shown in Figure S2). Time (minutes) is shown from bottom to top, the FID trace is shown on the left side and the aligned calcium traces on the right side. Each trace is the calcium recording of a single fly (here n = 5, fly names top right). The odorants added to MixC are shown in the middle (BDOL, MBAE, EM2E, ZHAE, FENT, MSAM, GEST) and for all odorants an FID signal is visible. Note that two strong calcium responses, without a corresponding FID signal, or known odorant application were observed here and marked as BEDNXX and EM2EXX (these contaminations were also observed in the single odorant measurements of BEDN and EM2E, hence the code). BDOL elicited the overlapping FID peaks (the first peak comes from the chiral pair SBDL and RBDL, and the second peak from the meso‐isomer MBDL). Since the peaks could not be separated for analysis, we stuck to the name of BDOL for the first peak and neglected the second (unlike for the polar column, see Figure S2). [file EJN-61-0-s003.tiff]

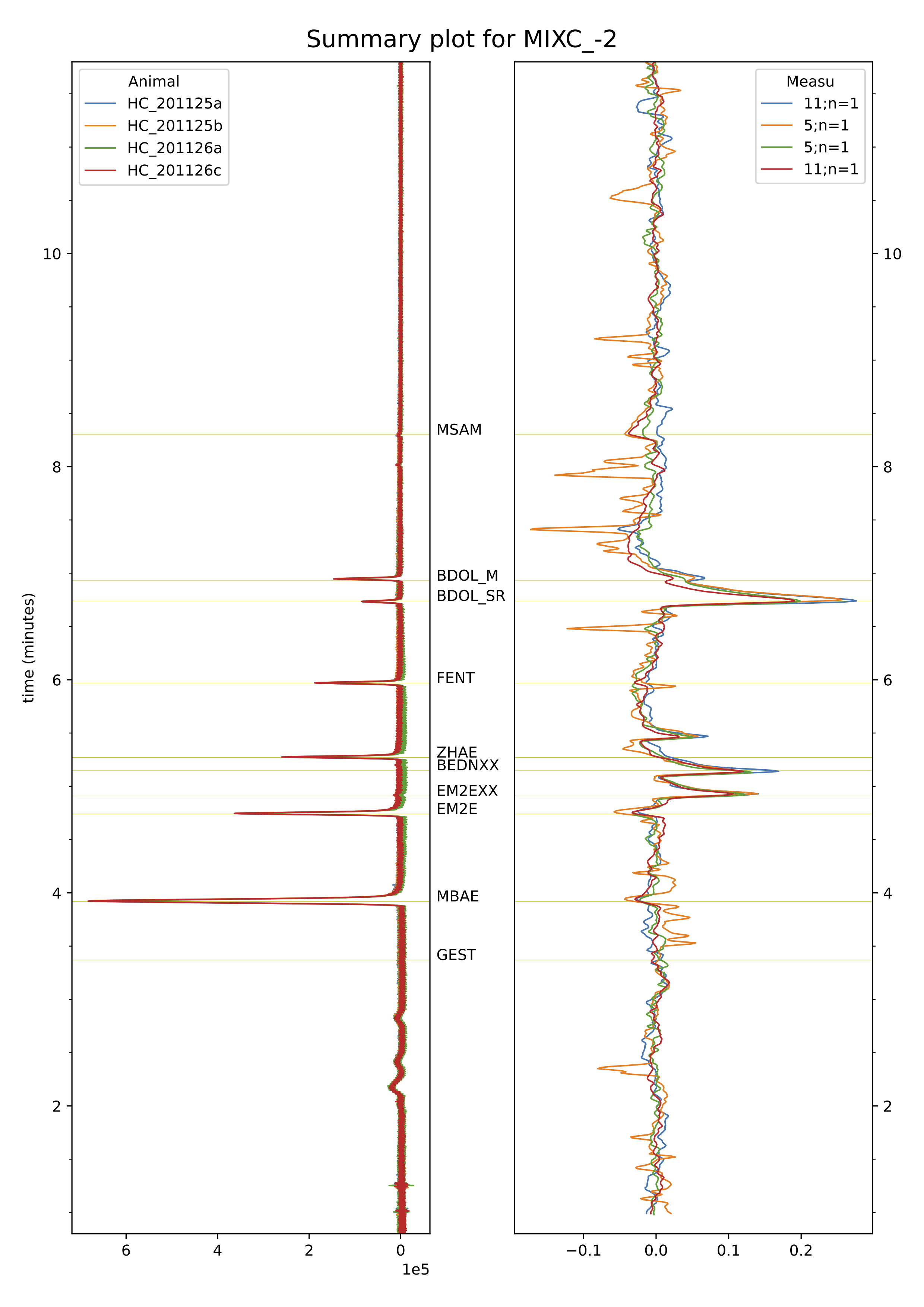

Supplement: Supplementary file 11 — Fig. S2 The summary plot for Or92a shows the time traces of the GC‐FID and of the calcium recording to MixC at a dilution of 10−2. In this experiment, the polar column was used (for comparison: MixC, separated by the nonpolar column, is shown in Figure S1). Time (minutes) is shown from bottom to top, the FID trace is shown on the left side and the aligned calcium traces on the right side. Each trace is the calcium recording of a single fly (here n = 4). The odorants added to MixC are shown in the middle (GEST, MBAE, EM2E, ZHAE, FENT, BDOL_SR, BDOL_M, MSAM). Note, that the order of the eluting odorants and the elution times are different from the nonpolar column (Figure S1). The two strong calcium responses to contaminations, named BEDNXX and EM2EXX, which appear without a corresponding FID signal or known odorant application in the mix, were also observed here and marked (these contaminations were also observed in the single odorant measurements of BEDN and EM2E). The racemic mixture BDOL elicited two clearly separated FID peaks on the polar column, corresponding to the elution times of SBDL/RBDL (first peak) and MBDL (second peak). Since the peaks could be separated for analysis, we labeled the two BDOL peaks as BDOL_SR and BDOL_M, respectively. [file EJN-61-0-s011.tiff]

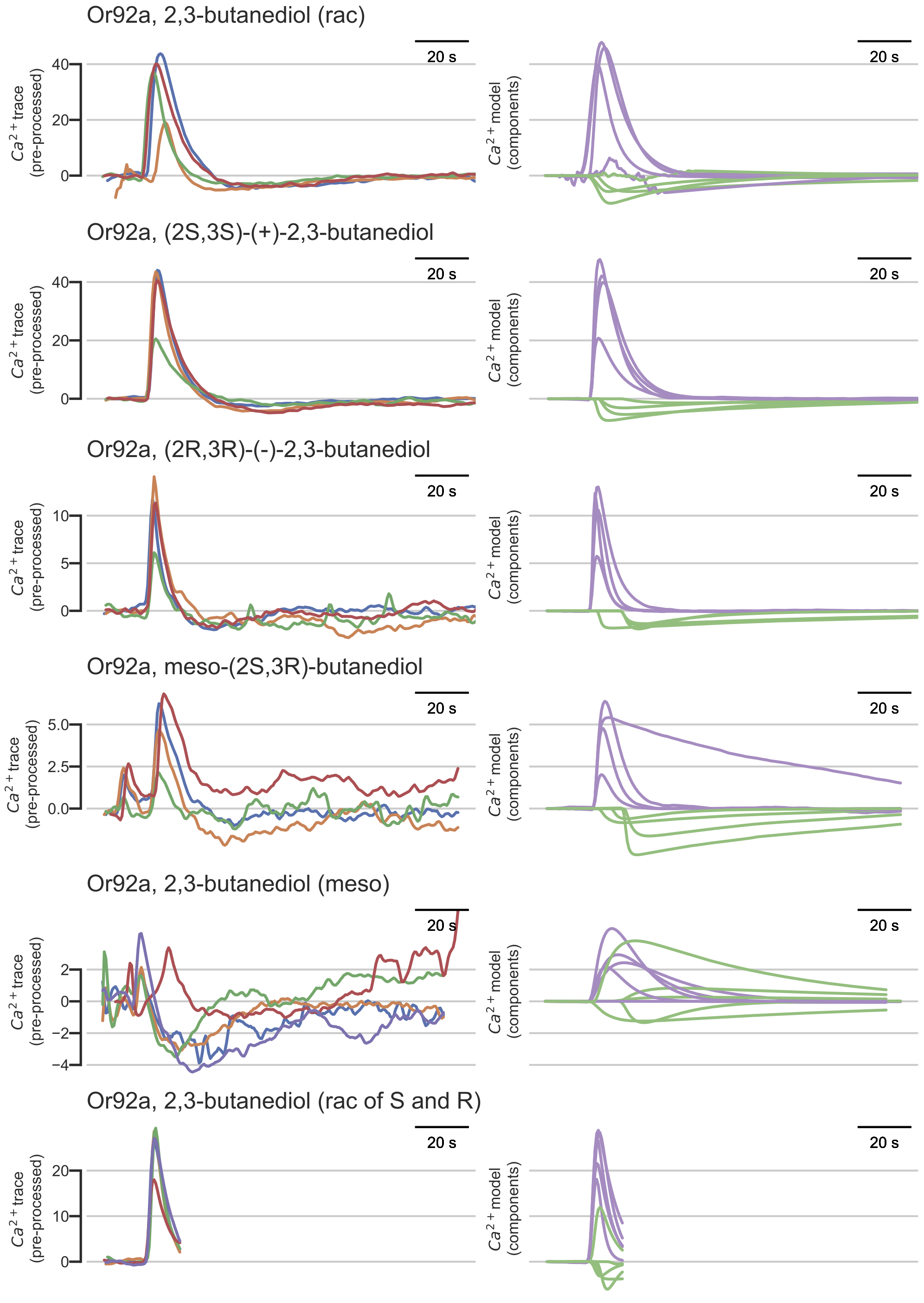

Supplement: Supplementary file 12 — Fig. S3 Responses to different chiral forms of 2,3‐butanediol in Or92a (left column), and corresponding linear‐non‐linear modeling (right column). All responses were biphasic: first positive, and then with an undershoot below baseline. Weaker responses (meso form) look graphically more noisy traces due to their scaling. The bottom trace is shorter because the following response to another substance must not be included in the modeling. [file EJN-61-0-s009.tiff]

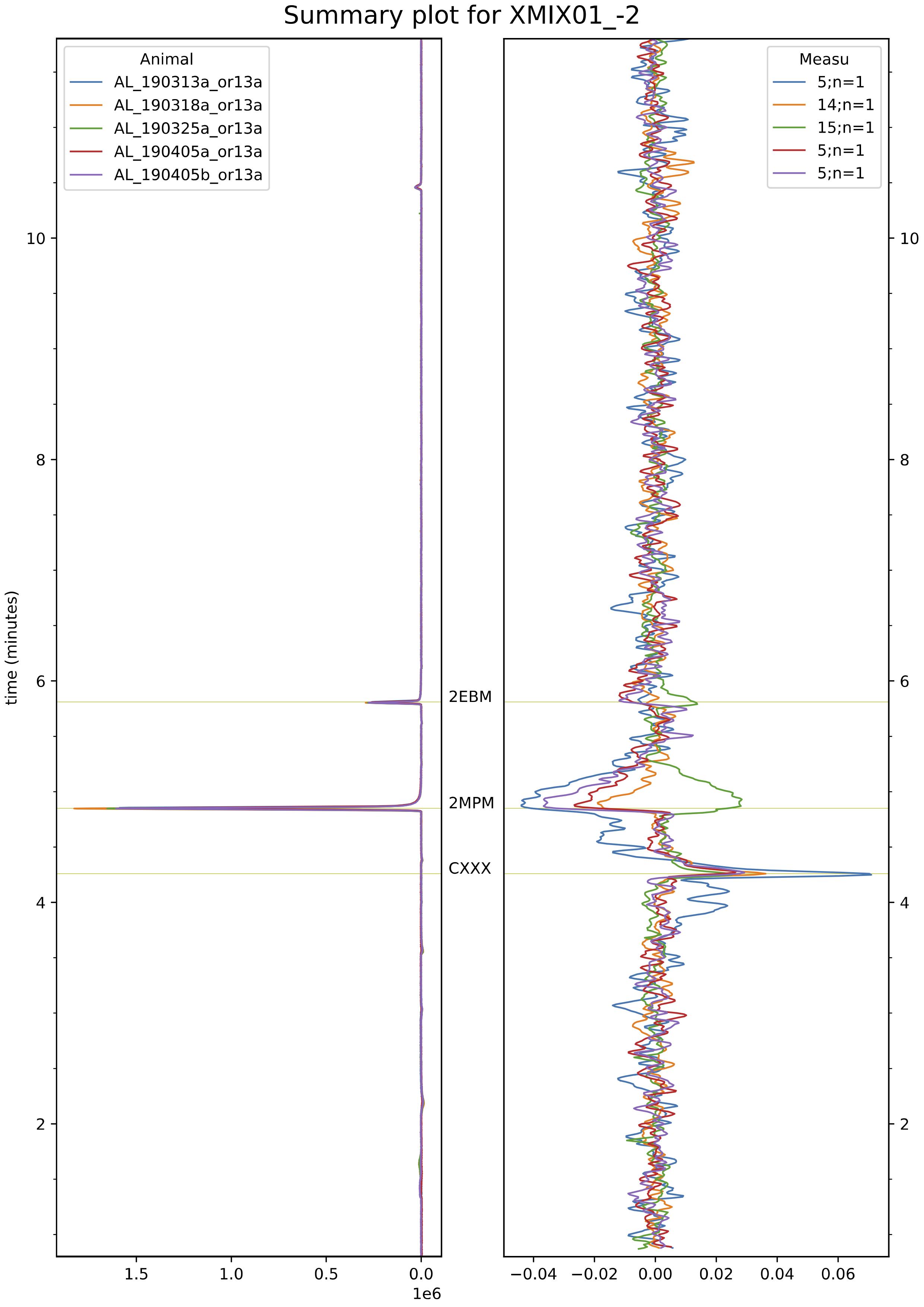

Supplement: Supplementary file 13 — Fig. S4 The summary plot of Or13a shows the only observed negative response in this Or line to the odorant 2‐methylphenol (2MPM). The negative response was dose dependent (not shown). Time (minutes) is shown from bottom to top, the FID trace is shown on the left side and the aligned calcium traces on the right side. Each trace is the calcium recording of a single fly (here n = 5). Interestingly, in four flies the response was clearly negative, while one fly gave a positive response. This behavior correlated with the weaker (and non‐significant) responses to 4MPM approx. one minute later. CXXX is a contamination with 1‐octen‐3‐ol, the best‐known ligand for Or13a. [file EJN-61-0-s002.tiff]

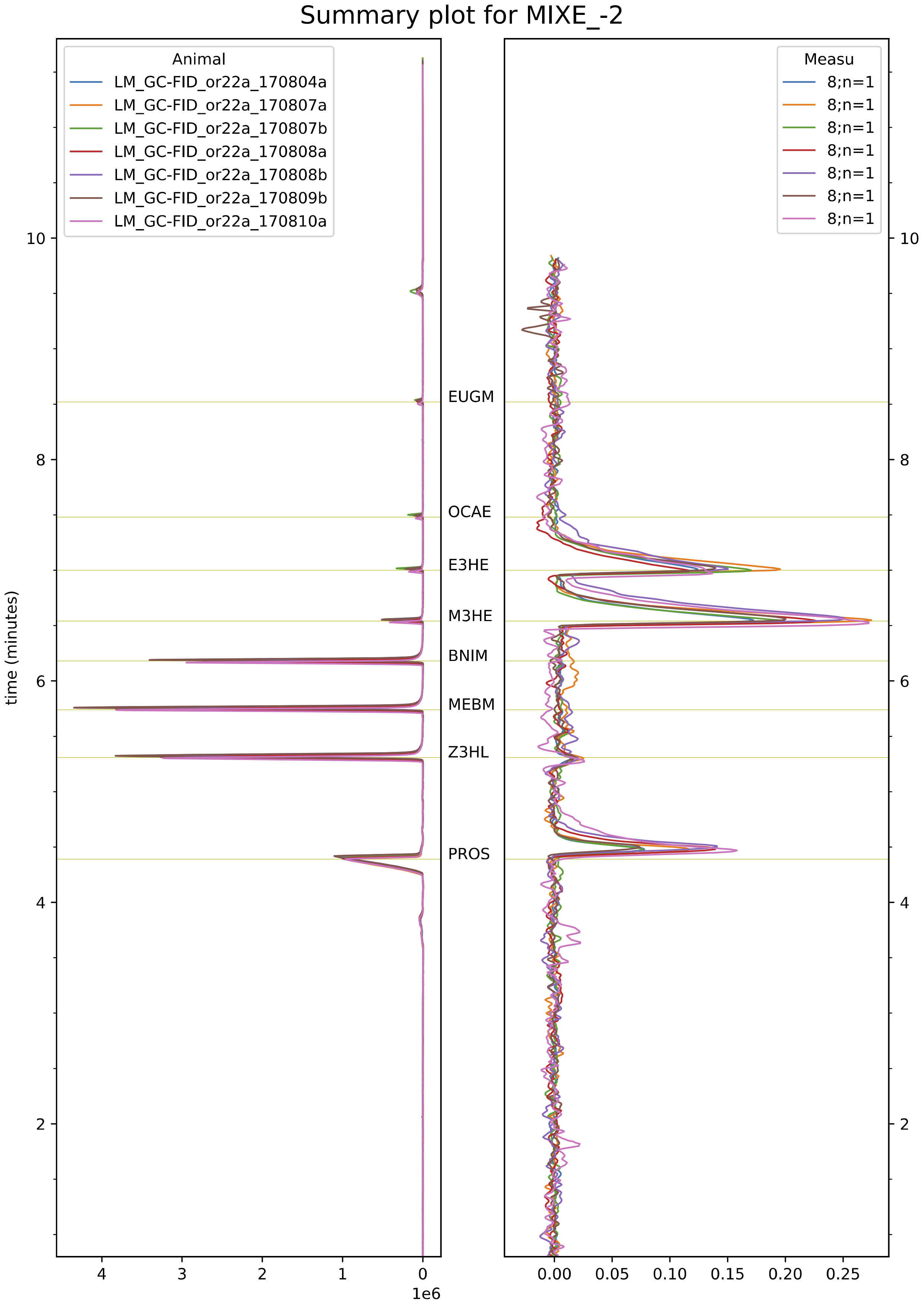

Supplement: Supplementary file 14 — Fig. S5 The summary plot of Or22a shows the calcium response to propanoic acid (PROS) in MixE (dilution 10−2). PROS consistently elicited an off response, i.e. a positive calcium response after stimulus offset, without a previous inhibitory “on”‐response during odor stimulation. This was the only stimulus to show this behavior in our dataset. [file EJN-61-0-s010.tiff]

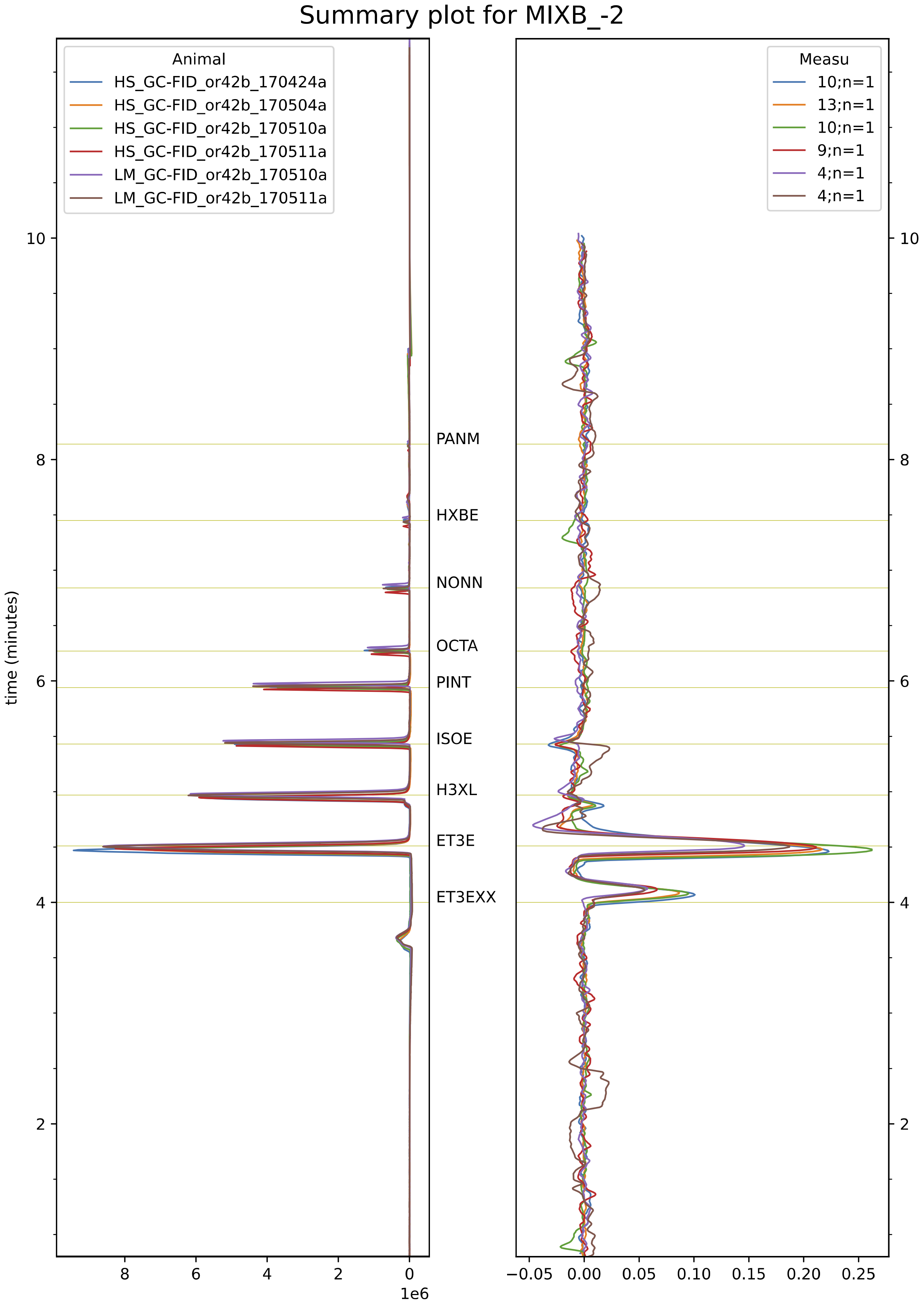

Supplement: Supplementary file 15 — Fig. S6 The summary plot of Or42b reveals a consistently strong calcium response to a contamination, occurring before ethyl propionate (ET3E). While ET3E eluted at 4:51 min (from the nonpolar column), the contamination (named ET3EXX) eluted at 4:11 min. The response to the contamination was about half the response to ET3E, but totally invisible in the FID trace, indicating that at this location a very strong ligand had eluted. Future experiments will be needed to identify this substance. [file EJN-61-0-s013.tiff]
